# Cusp Universality for Random Matrices I: Local Law and the Complex Hermitian Case

**DOI:** 10.1007/s00220-019-03657-4

**Published:** 2020-04-28

**Authors:** László Erdős, Torben Krüger, Dominik Schröder

**Affiliations:** 1 IST Austria, Am Campus 1, 3400 Klosterneuburg, Austria; 2 University of Bonn, Endenicher Allee 60, 53115 Bonn, Germany; 3 Present Address: Institute for Theoretical Studies, ETH Zurich, Clausiusstr. 47, 8092 Zurich, Switzerland

## Abstract

For complex Wigner-type matrices, i.e. Hermitian random matrices with independent, not necessarily identically distributed entries above the diagonal, we show that at any cusp singularity of the limiting eigenvalue distribution the local eigenvalue statistics are universal and form a Pearcey process. Since the density of states typically exhibits only square root or cubic root cusp singularities, our work complements previous results on the bulk and edge universality and it thus completes the resolution of the Wigner–Dyson–Mehta universality conjecture for the last remaining universality type in the complex Hermitian class. Our analysis holds not only for exact cusps, but approximate cusps as well, where an extended Pearcey process emerges. As a main technical ingredient we prove an optimal local law at the cusp for both symmetry classes. This result is also the key input in the companion paper (Cipolloni et al. in Pure Appl Anal, 2018. arXiv:1811.04055) where the cusp universality for real symmetric Wigner-type matrices is proven. The novel cusp fluctuation mechanism is also essential for the recent results on the spectral radius of non-Hermitian random matrices (Alt et al. in Spectral radius of random matrices with independent entries, 2019. arXiv:1907.13631), and the non-Hermitian edge universality (Cipolloni et al. in Edge universality for non-Hermitian random matrices, 2019. arXiv:1908.00969).

## Introduction

The celebrated Wigner–Dyson–Mehta (WDM) conjecture asserts that local eigenvalue statistics of large random matrices are universal: they only depend on the symmetry type of the matrix and are otherwise independent of the details of the distribution of the matrix ensemble. This remarkable spectral robustness was first observed by Wigner in the bulk of the spectrum. The correlation functions are determinantal and they were computed in terms the *sine kernel* via explicit Gaussian calculations by Dyson, Gaudin and Mehta [59]. Wigner’s vision continues to hold at the spectral edges, where the correct statistics was identified by Tracy and Widom for both symmetry types in terms of the *Airy kernel* [70, 71]. These universality results have been originally formulated and proven [17, 35, 36, 67–69] for traditional *Wigner matrices*, i.e. Hermitian random matrices with independent, identically distributed (i.i.d.) entries and their diagonal [55, 57] and non-diagonal [51] deformations. More recently they have been extended to *Wigner-type ensembles*, where the identical distribution is not required, and even to a large class of matrices with general correlated entries [7, 8, 11]. In different directions of generalization, sparse matrices [1, 32, 47, 56], adjacency matrices of regular graphs [14] and band matrices [19, 20, 66] have also been considered. In parallel developments bulk and edge universal statistics have been proven for invariant $\beta$-ensembles [12, 15, 17, 18, 29, 30, 52, 61, 62, 64, 65, 73] and even for their discrete analogues [13, 16, 41, 48] but often with very different methods.

A precondition for the Tracy-Widom distribution in all these generalizations of Wigner’s original ensemble is that the density of states vanishes as a square root near the spectral edges. The recent classification of the singularities of the solution to the underlying Dyson equation indeed revealed that at the edges only square root singularities appear [6, 10]. The density of states may also form a cusp-like singularity in the interior of the asymptotic spectrum, i.e. single points of vanishing density with a cubic root growth behaviour on either side. Under very general conditions, no other type of singularity may occur. At the cusp a new local eigenvalue process emerges: the correlation functions are still determinantal but the *Pearcey kernel* replaces the sine- or the Airy kernel.

The Pearcey process was first established by Brézin and Hikami for the eigenvalues close to a cusp singularity of a deformed complex Gaussian Wigner (GUE) matrix. They considered the model of a GUE matrix plus a deterministic matrix (“external source”) having eigenvalues $\pm 1$ with equal multiplicity [21, 22]. The name *Pearcey kernel* and the corresponding *Pearcey process* have been coined by [72] in reference to related functions introduced by Pearcey in the context of electromagnetic fields [63]. Similarly to the universal sine and Airy processes, it has later been observed that also the Pearcey process universality extends beyond the realm of random matrices. Pearcey statistics have been established for non-intersecting Brownian bridges [3] and in skew plane partitions [60], always at criticality. We remark, however, that critical cusp-like singularity does not always induce a Pearcey kernel, see e.g. [31].

In random matrix theory there are still only a handful of rather specific models for which the emergence of the Pearcey process has been proven. This has been achieved for deformed GUE matrices [2, 4, 23] and for Gaussian sample covariance matrices [42–44] by a contour integration method based upon the Brézin–Hikami formula. Beyond linear deformations, the Riemann-Hilbert method has been used for proving Pearcey statistics for a certain *two-matrix model* with a special quartic potential with appropriately tuned coefficients [40]. All these previous results concern only specific ensembles with a matrix integral representation. In particular, Wigner-type matrices are out of the scope of this approach.

The main result of the current paper is the proof of the Pearcey universality at the cusps for complex Hermitian Wigner-type matrices under very general conditions. Since the classification theorem excludes any other singularity, this is the third and last universal statistics that emerges from natural generalizations of Wigner’s ensemble.

This third universality class has received somewhat less attention than the other two, presumably because cusps are not present in the classical Wigner ensemble. We also note that the most common invariant $\beta$-ensembles do not exhibit the Pearcey statistics as their densities do not feature cubic root cusps but are instead 1/2-Hölder continuous for somewhat regular potentials [28]. The density vanishes either as 2*k*th or $(2k +\frac{1}{2})$th power with their own local statistics (see [26] also for the persistence of these statistics under small additive GUE perturbations before the critical time). Cusp singularities, hence Pearcey statistics, however, naturally arise within any one-parameter family of Wigner-type ensembles whenever two spectral bands merge as the parameter varies. The classification theorem implies that cusp formation is the only possible way for bands to merge, so in that sense Pearcey universality is ubiquitous as well.

The bulk and edge universality is characterized by the symmetry type alone: up to a natural shift and rescaling there is only one bulk and one edge statistic. In contrast, the cusp universality has a much richer structure: it is naturally embedded in a one-parameter family of universal statistics within each symmetry class. In the complex Hermitian case these are given by the one-parameter family of (extended) Pearcey kernels, see (2.5) later. Thinking in terms of fine-tuning a single parameter in the space of Wigner-type ensembles, the density of states already exhibits a universal local shape right before and right after the cusp formation; it features a tiny gap or a tiny nonzero local minimum, respectively [5, 10]. When the local lengthscale $\ell$ of these *almost cusp* shapes is comparable with the local eigenvalue spacing $\delta$, then the general Pearcey statistics is expected to emerge whose parameter is determined by the ratio $\ell /\delta$. Thus the full Pearcey universality typically appears in a *double scaling limit*.

Our proof follows the *three step strategy* that is the backbone of the recent approach to the WDM universality, see [38] for a pedagogical exposé and for detailed history of the method. The first step in this strategy is a *local law* that identifies, with very high probability, the empirical eigenvalue distribution on a scale slightly above the typical eigenvalue spacing. The second step is to prove universality for ensembles with a tiny Gaussian component. Finally, in the third step this Gaussian component is removed by perturbation theory. The local law is used for precise apriori bounds in the second and third steps.

The main novelty of the current paper is the proof of the local law at optimal scale near the cusp. To put the precision in proper context, we normalize the $N\times N$ real symmetric or complex Hermitian Wigner-type matrix *H* to have norm of order one. As customary, the local law is formulated in terms of the Green function $G(z):=(H-z)^{-1}$ with spectral parameter *z* in the upper half plane. The local law then asserts that *G*(*z*) becomes deterministic in the large *N* limit as long as $\eta :=\mathfrak {I}z$ is much larger than the local eigenvalue spacing around $\mathfrak {R}z$. The deterministic approximant *M*(*z*) can be computed as the unique solution of the corresponding Dyson equation (see (2.2) and (3.1) later). Near the cusp the typical eigenvalue spacing is of order $N^{-3/4}$; compare this with the $N^{-1}$ spacing in the bulk and $N^{-2/3}$ spacing near the edges. We remark that a local law at the cusp on the non-optimal scale $N^{-3/5}$ has already been proven in [8]. In the current paper we improve this result to the optimal scale $N^{-3/4}$ and this is essential for our universality proof at the cusp.

The main ingredient behind this improvement is an optimal estimate of the error term *D* (see (3.4) later) in the approximate Dyson equation that *G*(*z*) satisfies. The difference $M-G$ is then roughly estimated by ${{\mathcal {B}}}^{-1} (MD)$, where ${{\mathcal {B}}}$ is the linear stability operator of the Dyson equation. Previous estimates on *D* (in averaged sense) were of order $\rho /N\eta$, where $\rho$ is the local density; roughly speaking $\rho \sim 1$ in the bulk, $\rho \sim N^{-1/3}$ at the edge and $\rho \sim N^{-1/4}$ near the cusp. While this estimate cannot be improved in general, our main observation is that, to leading order, we need only the projection of *MD* in the single unstable direction of ${{\mathcal {B}}}$. We found that this projection carries an extra hidden cancellation due to a special local symmetry at the cusp and thus the estimate on *D* effectively improves to $\rho ^2/N\eta$. Customary power counting is not sufficient, we need to compute this error term explicitly at least to leading order. We call this subtle mechanism *cusp fluctuation averaging* since it combines the well established fluctuation averaging procedure with the additional cancellation at the cusp. Similar estimates extend to the vicinity of the exact cusps. We identify a key quantity, denoted by $\sigma (z)$ (in (3.5b) later), that measures the distance from the cusp in a canonical way: $\sigma (z)=0$ characterizes an exact cusp, while $\left| \sigma (z)\right| \ll 1$ indicates that *z* is near an almost cusp. Our final estimate on *D* is of order $(\rho +\left| \sigma \right| )\rho /N\eta$. Since the error term *D* is random and we need to control it in high moment sense, we need to lift this idea to a high moment calculation, meticulously extracting the improvement from every single term. This is performed in the technically most involved Sect. 4 where we use a Feynman diagrammatic formalism to bookkeep the contributions of all terms. Originally we have developed this language in [34] to handle random matrices with slow correlation decay, based on the revival of the cumulant expansion technique in [45] after [50]. In the current paper we incorporate the cusp into this analysis. We identify a finite set of Feynman subdiagrams, called $\sigma$-*cells* (Definition 4.10) with value $\sigma$ that embody the cancellation effect at the cusp. To exploit the full strength of the cusp fluctuation averaging mechanism, we need to trace the fate of the $\sigma$-cells along the high moment expansion. The key point is that $\sigma$-cells are local objects in the Feynman graphs thus their cancellation effects act simultaneously and the corresponding gains are multiplicative.

Formulated in the jargon of diagrammatic field theory, extracting the deterministic Dyson equation for *M* from the resolvent equation $(H-z)G(z)=1$ corresponds to a consistent self-energy renormalization of *G*. One way or another, such procedure is behind every proof of the optimal local law with high probability. Our $\sigma$-cells conceptually correspond to a next order resummation of certain Feynman diagrams carrying a special cancellation.

We remark that we prove the optimal local law only for Wigner-type matrices and not yet for general correlated matrices unlike in [11, 34]. In fact we use the simpler setup only for the estimate on *D* (Theorem 3.7) the rest of the proof is already formulated for the general case. This simpler setup allows us to present the cusp fluctuation averaging mechanism with the least amount of technicalities. The extension to the correlated case is based on the same mechanism but it requires considerably more involved diagrammatic manipulations which is better to develop in a separate work to contain the length of this paper.

Our cusp fluctuation averaging mechanism has further applications. It is used in [9] to prove an optimal cusp local law for the Hermitization of non-Hermitian random matrices with a variance profile, demonstrating that the technique is also applicable in settings where the flatness assumption is violated. The cusp of the Hermitization corresponds to the edge of the non-Hermitian model via Girko’s formula, thus the optimal cusp local law leads to an optimal bound on the spectral radius [9] and ultimately also to edge universality [25] for non-Hermitian random matrices.

Armed with the optimal local law we then perform the other two steps of the three step analysis. The third step, relying on the *Green function comparison theorem*, is fairly standard and previous proofs used in the bulk and at the edge need only minor adjustments. The second step, extracting universality from an ensemble with a tiny Gaussian component can be done in two ways: (i) Brézin–Hikami formula with contour integration or (ii) Dyson Brownian Motion (DBM). Both methods require the local law as an input. In the current work we follow (i) mainly because this approach directly yields the Pearcey kernel, at least for the complex Hermitian symmetry class. In the companion work [24] we perform the DBM analysis adapting methods of [37, 53, 54] to the cusp. The main novelty in the current work and in [24] is the rigidity at the cusp on the optimal scale provided below. Once this key input is given, the proof of the edge universality from [53] is modified in [24] to the cusp setting, proving universality for the real symmetric case as well. We remark, however, that, to our best knowledge, the analogue of the Pearcey kernel for the real symmetric case has not yet been explicitly identified.

We now explain some novelty in the contour integration method. We first note that a similar approach was initiated in the fundamental work of Johansson on the bulk universality for Wigner matrices with a large Gaussian component in [49]. This method was generalised later to Wigner matrices with a small Gaussian component in [35] as well as it inspired the proof of bulk universality via the moment matching idea [68] once the necessary local law became available. The double scaling regime has also been studied, where the density is very small but the Gaussian component compensates for it [27]. More recently, the same approach was extended to the cusp for deformed GUE matrices [23, Theorem 1.3] and for sample covariance matrices but only for large Gaussian component [42–44]. For our cusp universality, we need to perform a similar analysis but with a small Gaussian component. We represent our matrix *H* as ${\widehat{H}} + \sqrt{t} U$, where *U* is GUE and ${\widehat{H}}$ is an independent Wigner-type matrix. The contour integration analysis (Sect. 5.1) requires a Gaussian component of size at least $t\gg N^{-1/2}$.

The input of the analysis in Sect. 5.1 for the correlation kernel of *H* is a very precise description of the eigenvalues of ${\widehat{H}}$ just above $N^{-3/4}$, the scale of the typical spacing between eigenvalues—this information is provided by our optimal local law. While in the bulk and in the regime of the regular edge finding an appropriate $\widehat{H}$ is a relatively simple matter, in the vicinity of a cusp point the issue is very delicate. The main reason is that the cusp, unlike the bulk or the regular edge, is unstable under small perturbations; in fact it typically disappears and turns into a small positive local minimum if a small GUE component is added. Conversely, a cusp emerges if a small GUE component is added to an ensemble that has a density with a small gap. In particular, even if the density function $\rho (\tau )$ of *H* exhibits an exact cusp, the density $\widehat{\rho }(\tau )$ of $\widehat{H}$ will have a small gap: in fact $\rho$ is given by the evolution of the semicircular flow up to time *t* with initial data $\widehat{\rho }$. Unlike in the bulk and edge cases, here one cannot match the density of *H* and $\widehat{H}$ by a simple shift and rescaling. Curiously, the contour integral analysis for the local statistics of *H* at the cusp relies on an optimal local law of $\widehat{H}$ with a small gap far away from the cusp.

Thus we need an additional ingredient: the precise analysis of the semicircular flow $\rho _s:=\widehat{\rho } \boxplus \rho _{\mathrm {sc}}^{(s)}$ near the cusp up to a relatively long times $s\lesssim N^{-1/2+\epsilon }$; note that $\rho _t=\rho$ is the original density with the cusp. Here $\rho _{\mathrm {sc}}^{(s)}$ is the semicircular density with variance *s* and $\boxplus$ indicates the free convolution. In Sects. 5.2–5.3 we will see that the edges of the support of the density $\rho _s$ typically move linearly in the time *s* while the gap closes at a much slower rate. Already $s\gg N^{-3/4}$ is beyond the simple perturbative regime of the cusp whose natural lengthscale is $N^{-3/4}$. Thus we need a very careful tuning of the parameters: the analysis of a cusp for *H* requires constructing a matrix $\widehat{H}$ that is far from having a cusp but that after a relatively long time $t=N^{-1/2+\epsilon }$ will develop a cusp exactly at the right location. In the estimates we heavily rely on various properties of the solution to the Dyson equation established in the recent paper [10]. These results go well beyond the precision of the previous work [5] and they apply to a very general class of Dyson equations, including a non-commutative von-Neumann algebraic setup.

**Notations.** We now introduce some custom notations we use throughout the paper. For non-negative functions *f*(*A*, *B*), *g*(*A*, *B*) we use the notation $f \le _A g$ if there exist constants *C*(*A*) such that $f(A,B)\le C(A) g(A,B)$ for all *A*, *B*. Similarly, we write $f\sim _A g$ if $f\le _A g$ and $g\le _A f$. We do not indicate the dependence of constants on basic parameters that will be called model parameters later. If the implied constants are universal, we instead write $f\lesssim g$ and $f\sim g$. Similarly we write $f \ll g$ if $f\le c g$ for some tiny absolute constant $c>0$.

We denote vectors by bold-faced lower case Roman letters $\mathbf {x},\mathbf {y}\in \mathbb {C}^N$, and matrices by upper case Roman letters $A,B\in \mathbb {C}^{N\times N}$ with entries $A=(a_{ij})_{i,j=1}^N$. The standard scalar product and Euclidean norm on $\mathbb {C}^N$ will be denoted by $\left\langle \mathbf {x},\mathbf {y}\right\rangle :=N^{-1}\sum _{i\in [N]}\overline{x_i}y_i$ and $\Vert \mathbf {x}\Vert$, while we also write $\left\langle A,B\right\rangle :=N^{-1}{{\,\mathrm{Tr}\,}}A^*B$ for the scalar product of matrices, and $\left\langle A\right\rangle :=N^{-1}{{\,\mathrm{Tr}\,}}A$, $\left\langle \mathbf {x}\right\rangle :=N^{-1}\sum _{a\in [N]}x_a$. We write ${{\,\mathrm{diag}\,}}R$, ${{\,\mathrm{diag}\,}}{\mathbf {r}}$ for the diagonal vector of a matrix *R* and the diagonal matrix obtained from a vector ${\mathbf {r}}$, and $S\odot R$ for the entrywise (Hadamard) product of matrices *R*, *S*. The usual operator norm induced by the vector norm $\Vert \cdot \Vert$ will be denoted by $\Vert A\Vert$, while the Hilbert-Schmidt (or Frobenius) norm will be denoted by $\Vert A\Vert _\text {hs}:=\sqrt{\left\langle A,A\right\rangle }$. For integers *n* we define $[n]:=\{1,\ldots ,n\}$.

## Main Results

### The Dyson equation

Let $W=W^* \in \mathbb {C}^{N \times N}$ be a self-adjoint random matrix and $A={{\,\mathrm{diag}\,}}({\varvec{a}})$ be a deterministic diagonal matrix with entries ${\varvec{a}}=(a_i)_{i=1}^N \in \mathbb {R}^N$. We say that *W* is of *Wigner-type* [8] if its entries $w_{ij}$ for $i \le j$ are centred, ${{\,\mathrm{\mathbf {E}}\,}}w_{ij} =0$, independent random variables. We define the *variance matrix* or *self-energy matrix* $S=(s_{ij})_{i,j=1}^N$ by

2.1 $$\begin{aligned} s_{ij}:={{\,\mathrm{\mathbf {E}}\,}}\left| w_{ij}\right| ^2. \end{aligned}$$

This matrix is symmetric with non-negative entries. In [8] it was shown that as *N* tends to infinity, the resolvent $G(z):=(H-z)^{-1}$ of the *deformed Wigner-type matrix* $H=A+W$ entrywise approaches a diagonal matrix

$$\begin{aligned} M(z):={{\,\mathrm{diag}\,}}({\mathbf {m}}(z)). \end{aligned}$$

The entries ${\mathbf {m}}=(m_1, \ldots , m_N):\mathbb {H}\rightarrow \mathbb {H}^N$ of *M* have positive imaginary parts and solve the *Dyson equation*

2.2 $$\begin{aligned} -\frac{1}{m_i(z)}= z-a_i +\sum _{j=1}^Ns_{ij}m_j(z),\qquad z \in \mathbb {H}:=\{z\in \mathbb {C}|\mathfrak {I}z>0\}, \quad i\in [N]. \end{aligned}$$

We call *M* or ${\mathbf {m}}$ the *self-consistent Green’s function*. The normalised trace of *M* is the Stieltjes transform of a unique probability measure on $\mathbb {R}$ that approximates the empirical eigenvalue distribution of $A+W$ increasingly well as $N \rightarrow \infty$, motivating the following definition.

#### Definition 2.1

*(Self-consistent density of states).* The unique probability measure $\rho$ on $\mathbb {R}$, defined through

$$\begin{aligned} \left\langle M(z)\right\rangle =\frac{1}{N}{{\,\mathrm{Tr}\,}}M(z)= \int \frac{\rho (\mathrm {d}\tau )}{\tau -z},\qquad z \in \mathbb {H}, \end{aligned}$$

is called the self-consistent density of states (scDOS). Accordingly, its support ${{\,\mathrm{supp}\,}}\rho$ is called self-consistent spectrum.

### Cusp universality

We make the following assumptions:

#### Assumption (A)

*(Bounded moments).* The entries of the Wigner-type matrix $\sqrt{N}W$ have bounded moments and the expectation *A* is bounded, i.e. there are positive $C_k$ such that

$$\begin{aligned} \left| a_i\right| \le C_0, \qquad {{\,\mathrm{\mathbf {E}}\,}}\left| w_{ij}\right| ^k \le C_kN^{-k/2}, \qquad k \in \mathbb {N}. \end{aligned}$$

#### Assumption (B)

*(Fullness).* If the matrix $W = W^* \in \mathbb {C}^{N \times N}$ belongs to the complex hermitian symmetry class, then we assume

2.3 $$\begin{aligned} \begin{aligned} \left( \begin{array}{cc} {{\,\mathrm{\mathbf {E}}\,}}(\mathfrak {R}w_{ij})^2&{} {{\,\mathrm{\mathbf {E}}\,}}(\mathfrak {R}w_{ij})(\mathfrak {I}w_{ij}) \\ {{\,\mathrm{\mathbf {E}}\,}}(\mathfrak {R}w_{ij})(\mathfrak {I}w_{ij}) &{} {{\,\mathrm{\mathbf {E}}\,}}(\mathfrak {I}w_{ij})^2 \end{array} \right) \ge \frac{c}{N} \mathbb {1}_{2 \times 2}, \end{aligned} \end{aligned}$$

as quadratic forms, for some positive constant $c>0$. If $W = W^T \in \mathbb {R}^{N \times N}$ belongs to the real symmetric symmetry class, then we assume ${{\,\mathrm{\mathbf {E}}\,}}w_{ij}^2 \ge \frac{c}{N}$.

#### Assumption (C)

*(Bounded self-consistent Green’s function).* In a neighbourhood of some fixed spectral parameter $\tau \in \mathbb {R}$ the self-consistent Green’s function is bounded, i.e. for positive $C,\kappa$ we have

$$\begin{aligned} \left| m_{i}(z)\right| \le C, \qquad z \in \tau +(-\kappa ,\kappa )+ \mathrm {i}\mathbb {R}^+. \end{aligned}$$

We call the constants appearing in Assumptions (A)–(C)*model parameters*. All generic constants *C* in this paper may implicitly depend on these model parameters. Dependence on further parameters however will be indicated.

#### Remark 2.2

The boundedness of ${\mathbf {m}}$ in Assumption (C) can be ensured by assuming some regularity of the variance matrix *S*. For more details we refer to [5, Chapter 6].

From the extensive analysis in [10] we know that the self-consistent density $\rho$ is described by explicit *shape functions* in the vicinity of local minima with small value of $\rho$ and around small gaps in the support of $\rho$. The density in such *almost cusp regimes* is given by precisely one of the following three asymptotics:

(i) *Exact cusp*. There is a cusp point $\mathfrak {c}\in \mathbb {R}$ in the sense that $\rho (\mathfrak {c})=0$ and $\rho (\mathfrak {c}\pm \delta )>0$ for $0\ne \delta \ll 1$. In this case the self-consistent density is locally around $\mathfrak {c}$ given by
  2.4a $$\begin{aligned} \rho (\mathfrak {c}\pm x) = \frac{\sqrt{3}\gamma ^{4/3}}{2\pi } x^{1/3} \Big [1+{\mathcal {O}}\,\left( x^{1/3}\right) \Big ],\qquad x\ge 0 \end{aligned}$$
  for some $$\gamma >0$$.
(ii) *Small gap.* There is a maximal interval $[\mathfrak {e}_-,\mathfrak {e}_+]$ of size $0<\Delta :=\mathfrak {e}_+-\mathfrak {e}_-\ll 1$ such that $\rho |_{[\mathfrak {e}_-,\mathfrak {e}_+]}\equiv 0$. In this case the density around $\mathfrak {e}_\pm$ is, for some $\gamma >0$, locally given by
  2.4b $$\begin{aligned} \rho (\mathfrak {e}_\pm \pm x)=\frac{\sqrt{3}(2\gamma )^{4/3}\Delta ^{1/3}}{2\pi }\Psi _{\mathrm {edge}}(x/\Delta )\left[ 1+{\mathcal {O}}\,\left( \Delta ^{1/3}\Psi _{\mathrm {edge}}(x/\Delta )\right) \right] ,\qquad x\ge 0\nonumber \\ \end{aligned}$$
  where the shape function around the edge is given by
  2.4c $$\begin{aligned} \Psi _{\mathrm {edge}}(\lambda ):=\frac{\sqrt{\lambda (1+\lambda )}}{(1+2\lambda +2\sqrt{\lambda (1+\lambda )})^{2/3}+(1+2\lambda -2\sqrt{\lambda (1+\lambda )})^{2/3}+1},\quad \lambda \ge 0.\nonumber \\ \end{aligned}$$
(iii) *Non-zero local minimum.* There is a local minimum at $\mathfrak {m}\in \mathbb {R}$ of $\rho$ such that $0<\rho (\mathfrak {m})\ll 1$. In this case there exists some $\gamma >0$ such that
  2.4d $$\begin{aligned} \rho (\mathfrak {m}+ x) = \rho (\mathfrak {m}) + \rho (\mathfrak {m}) \Psi _{\mathrm {min}}\left( \frac{3\sqrt{3} \gamma ^4 x}{2(\pi \rho (\mathfrak {m}))^3 }\right) \left[ 1+{\mathcal {O}}\,\left( \rho (\mathfrak {m})^{1/2}+ \frac{\left| x\right| }{\rho (\mathfrak {m})^3}\right) \right] ,\quad x\in \mathbb {R}\nonumber \\ \end{aligned}$$
  where the shape function around the local minimum is given by
  2.4e $$\begin{aligned} \Psi _{\mathrm {min}}(\lambda ) :=\frac{\sqrt{1+\lambda ^2}}{(\sqrt{1+\lambda ^2}+\lambda )^{2/3}+(\sqrt{1+\lambda ^2}-\lambda )^{2/3}-1}-1,\qquad \lambda \in \mathbb {R}.\quad \end{aligned}$$

We note that the parameter $\gamma$ in (2.4a) is chosen in a way which is convenient for the universality statement. We also note that the choices for $\gamma$ in (2.4b)–(2.4d) are consistent with (2.4a) in the sense that in the regimes $\Delta \ll x\ll 1$ and $\rho (\mathfrak {m})^3\ll \left| x\right| \ll 1$ the respective formulae asymptotically agree. Depending on the three cases (i)–(iii), we define the *almost cusp point* $\mathfrak {b}$ as the cusp $\mathfrak {c}$ in case (i), the midpoint $(\mathfrak {e}_-+\mathfrak {e}_+)/2$ in case (ii), and the minimum $\mathfrak {m}$ in case (iii). When the local length scale of the almost cusp shape starts to match the eigenvalue spacing, i.e. if $\Delta \lesssim N^{-3/4}$ or $\rho (\mathfrak {m})\lesssim N^{-1/4}$, then we call the local shape a *physical cusp*. This terminology reflects the fact that the shape becomes indistinguishable from the exact cusp with $\rho (\mathfrak {c})=0$ when resolved with a precision above the eigenvalue spacing. In this case we call $\mathfrak {b}$ a *physical cusp point*.

The extended Pearcey kernel with a real parameter $\alpha$ (often denoted by $\tau$ in the literature) is given by

2.5 $$\begin{aligned} K_\alpha (x,y) = \frac{1}{(2\pi \mathrm {i})^2} \int _\Xi \mathrm{d}z \int _\Phi \mathrm{d}w \frac{\exp (-w^4/4 + \alpha w^2/2-yw + z^4/4-\alpha z^2/2 + xz)}{w-z},\nonumber \\ \end{aligned}$$

where $\Xi$ is a contour consisting of rays from $\pm \infty e^{\mathrm {i}\pi /4}$ to 0 and rays from 0 to $\pm \infty e^{-\mathrm {i}\pi /4}$, and $\Phi$ is the ray from $-\mathrm {i}\infty$ to $\mathrm {i}\infty$. The simple Pearcey kernel with parameter $\alpha =0$ has been first observed in the context of random matrix theory by [21, 22]. We note that (2.5) is a special case of a more general extended Pearcey kernel defined in [72, Eq. (1.1)].

It is natural to express universality in terms of a rescaled *k*-point function $p_k^{(N)}$ which we define implicitly by

$$\begin{aligned} \left( {\begin{array}{c}N\\ k\end{array}}\right) ^{-1} \sum _{\{i_1,\ldots ,i_k\}\subset [N]} {{\,\mathrm{\mathbf {E}}\,}}f(\lambda _{i_1},\ldots ,\lambda _{i_k}) = \int _{\mathbb {R}^k} f(x_1,\ldots ,x_k)p_k^{(N)}(x_1,\ldots ,x_k)\mathrm{d}x_1\ldots \mathrm{d}x_k \end{aligned}$$

for test functions *f*, where the summation is over all subsets of *k* distinct integers from [*N*].

#### Theorem 2.3

Let *H* be a complex Hermitian Wigner matrix satisfying Assumptions (A)–(C). Assume that the self-consistent density $\rho$ within $[\tau -\kappa ,\tau +\kappa ]$ from Assumption (C) has a physical cusp, i.e. that $\rho$ is locally given by (2.4) for some $\gamma >0$ and $\rho$ either (i) has a cusp point $\mathfrak {c}$, or (ii) a small gap $[\mathfrak {e}_-,\mathfrak {e}_+]$ of size $\Delta :=\mathfrak {e}_+-\mathfrak {e}_-\lesssim N^{-3/4}$, or (iii) a local minimum at $\mathfrak {m}$ of size $\rho (\mathfrak {m})\lesssim N^{-1/4}$. Then it follows that for any smooth compactly supported test function $F:\mathbb {R}^k\rightarrow \mathbb {R}$ it holds that

$$\begin{aligned} \left| \int _{\mathbb {R}^k} F({\varvec{x}})\left[ \frac{N^{k/4}}{\gamma ^k} p_k^{(N)}\left( \mathfrak {b}+ \frac{{\varvec{x}}}{\gamma N^{3/4}}\right) - \det (K_\alpha (x_i,x_j))_{i,j=1}^k\right] \mathrm{d}{\varvec{x}}\right| = {\mathcal {O}}\,\left( N^{-c(k)}\right) , \end{aligned}$$

where

2.6 $$\begin{aligned} \mathfrak {b}:={\left\{ \begin{array}{ll} \mathfrak {c}&{} \text {in case (i)},\\ (\mathfrak {e}_++\mathfrak {e}_-)/2 &{} \text {in case (ii)},\\ \mathfrak {m}&{} \text {in case (iii)}, \end{array}\right. }\qquad \alpha :={\left\{ \begin{array}{ll} 0 &{} \text {in case (i)}\\ 3 \left( \gamma \Delta /4\right) ^{2/3} N^{1/2} &{} \text {in case (ii)},\\ -\left( \pi \rho (\mathfrak {m})/\gamma \right) ^2 N^{1/2} &{} \text {in case (iii)}, \end{array}\right. }\nonumber \\ \end{aligned}$$

${\varvec{x}}=(x_1,\ldots ,x_k)$, $\mathrm{d}{\varvec{x}}=\mathrm{d}x_1\ldots \mathrm{d}x_k$, and $c(k)>0$ is a small constant only depending on *k*.

### Local law

We emphasise that the proof of Theorem 2.3 requires a very precise a priori control on the fluctuation of the eigenvalues even at singular points of the scDOS. This control is expressed in the form of a *local law* with an optimal convergence rate down to the typical eigenvalue spacing. We now define the scale on which the eigenvalues are predicted to fluctuate around the spectral parameter $\tau$.

#### Definition 2.4

*(Fluctuation scale).* We define the self-consistent fluctuation scale $\eta _{\mathrm {f}}=\eta _{\mathrm {f}}(\tau )$ through

$$\begin{aligned} \int _{-\eta _{\mathrm {f}}}^{ \eta _{\mathrm {f}}} \rho (\tau +\omega ) \mathrm {d}\omega = \frac{1}{N}, \end{aligned}$$

if $\tau \in {{\,\mathrm{supp}\,}}\rho$. If $\tau \not \in {{\,\mathrm{supp}\,}}\rho$, then $\eta _{\mathrm {f}}$ is defined as the fluctuation scale at a nearby edge. More precisely, let *I* be the largest (open) interval with $\tau \in I \subseteq \mathbb {R}{\setminus } {{\,\mathrm{supp}\,}}\rho$ and set $\Delta :=\min \{\left| I\right| ,1\}$. Then we define

2.7 $$\begin{aligned} \begin{aligned} \eta _{\mathrm {f}}:={\left\{ \begin{array}{ll} \Delta ^{1/9}/N^{2/3}, &{} \Delta > 1/N^{3/4}, \\ 1/N^{3/4}, &{} \Delta \le 1/N^{3/4}. \end{array}\right. } \end{aligned} \end{aligned}$$

We will see later (cf. (A.8b)) that (2.7) is the fluctuation of the edge eigenvalue adjacent to a spectral gap of length $\Delta$ as predicted by the local behaviour of the scDOS. The control on the fluctuation of eigenvalues is expressed in terms of the following local law.

#### Theorem 2.5

(Local law). Let *H* be a deformed Wigner-type matrix of the real symmetric or complex Hermitian symmetry class. Fix any $\tau \in \mathbb {R}$. Assuming (A)–(C) for any $\epsilon ,\zeta >0$ and $\nu \in \mathbb {N}$ the local law holds uniformly for all $z=\tau + \mathrm {i}\eta$ with ${{\,\mathrm{dist}\,}}(z,{{\,\mathrm{supp}\,}}\rho ) \in [N^\zeta \eta _{\mathrm {f}}(\tau ),N^{100}]$ in the form

2.8a $$\begin{aligned} \mathbf {P}\Bigg [\left| \left\langle {\mathbf {u}}, (G(z)-M(z))\mathbf {v}\right\rangle \right| \ge N^\epsilon \sqrt{\frac{\rho (z)}{N \eta }} \Vert {\mathbf {u}}\Vert \Vert \mathbf {v}\Vert \Bigg ]\le \frac{C}{N^\nu }, \end{aligned}$$

for any ${\mathbf {u}},\mathbf {v}\in \mathbb {C}^{N}$ and

2.8b $$\begin{aligned} \mathbf {P}\Bigg [ \left| \left\langle B (G(z)-M(z)\right\rangle \right| \ge \frac{N^\epsilon \Vert {B}\Vert }{N {{\,\mathrm{dist}\,}}(z,{{\,\mathrm{supp}\,}}\rho )}\Bigg ]\le \frac{C}{N^\nu }, \end{aligned}$$

for any $B \in \mathbb {C}^{N \times N}$. Here $\rho (z):=\left\langle \mathfrak {I}M(z)\right\rangle /\pi$ denotes the harmonic extension of the scDOS to the complex upper half plane. The constants $C>0$ in (2.8) only depends on $\epsilon ,\zeta ,\nu$ and the model parameters.

We remark that later we will prove the local law also in a form which is uniform in $\tau \in [-N^{100},N^{100}]$ and $\eta \in [ N^{-1+\zeta }, N^{100}]$, albeit with a more complicated error term, see Proposition 3.11. The local law Theorem 2.5 implies a large deviation result for the fluctuation of eigenvalues on the optimal scale uniformly for all singularity types.

#### Corollary 2.6

(Uniform rigidity). Let *H* be a deformed Wigner-type matrix of the real symmetric or complex Hermitian symmetry class satisfying Assumptions (A)–(C) for $\tau \in {{\,\mathrm{int}\,}}({{\,\mathrm{supp}\,}}\rho )$. Then

$$\begin{aligned} \mathbf {P}\big [ \left| \lambda _{k(\tau )}-\tau \right| \ge N^\epsilon \eta _{\mathrm {f}}(\tau ) \big ] \le \frac{C}{N^\nu } \end{aligned}$$

for any $\epsilon >0$ and $\nu \in \mathbb {N}$ and some $C=C(\epsilon ,\nu )$, where we defined the (self-consistent) eigenvalue index $k(\tau ):=\lceil N\rho ((-\infty , \tau ))\rceil$, and where $\lceil x\rceil =\min \{k\in \mathbb {Z}|k\ge x\}$.

In particular, the fluctuation of the eigenvalue whose expected position is closest to the cusp location does not exceed $N^{-3/4+\epsilon }$ for any $\epsilon >0$ with very high probability. The following corollary specialises Corollary 2.6 to the neighbourhood of a cusp.

#### Corollary 2.7

(Cusp rigidity). Let *H* be a deformed Wigner-type matrix of the real symmetric or complex Hermitian symmetry class satisfying Assumptions (A)–(C) and $\tau =\mathfrak {c}$ the location of an *exact cusp*. Then $N\rho ((-\infty , \mathfrak {c})) = k_\mathfrak {c}$ for some $k_\mathfrak {c}\in [N]$, that we call the cusp eigenvalue index. For any $\epsilon >0$, $\nu \in \mathbb {N}$ and $k \in [N]$ with $\left| k-k_\mathfrak {c}\right| \le c N$ we have

$$\begin{aligned} \mathbf {P}\bigg [ \left| \lambda _k-\gamma _k\right| \ge \frac{N^\epsilon }{(1+\left| k-k_\mathfrak {c}\right| )^{1/4}N^{3/4}}\bigg ]\le \frac{C}{N^\nu }, \end{aligned}$$

where $C=C(\epsilon ,\nu )$ and $\gamma _k$ are the self-consistent eigenvalue locations, defined through $N\rho ((-\infty , \gamma _k)) = k$.

We remark that a variant of Corollary 2.7 holds more generally for almost cusp points. It is another consequence of Corollary 2.6 that with high probability there are no eigenvalues much further than the fluctuation scale $\eta _{\mathrm {f}}$ away from the spectrum. We note that the following corollary generalises [11, Corollary 2.3] by also covering internal gaps of size $\ll 1$.

#### Corollary 2.8

(No eigenvalues outside the support of the self-consistent density). Let $\tau \not \in {{\,\mathrm{supp}\,}}\rho$. Under the assumptions of Theorem 2.5 we have

$$\begin{aligned} \mathbf {P}\Big [\exists \lambda \in {{\,\mathrm{Spec}\,}}H\cap [\tau -c,\tau +c], {{\,\mathrm{dist}\,}}(\lambda ,{{\,\mathrm{supp}\,}}\rho )\ge N^{\epsilon }\eta _{\mathrm {f}}(\tau )\Big ]\le C N^{-\nu }, \end{aligned}$$

for any $\epsilon ,\nu >0$, where *c* and *C* are positive constants, depending on model parameters. The latter also depends on $\epsilon$ and $\nu$.

#### Remark 2.9

Theorem 2.5 and its consequences, Corollaries 2.6, 2.7 and 2.8 also hold for both symmetry classes if Assumption (B) is replaced by the condition that there exists an $L \in \mathbb {N}$ and $c>0$ such that $\min _{i,j}(S^L)_{ij} \ge c/N$. A variance profile S satisfying this condition is called uniformly primitive (cf. [6, Eq. (2.5)] and [5, Eq. (2.11)]). Note that uniform primitivity is weaker than condition (B) on two accounts. First, it involves only the variance matrix ${{\,\mathrm{\mathbf {E}}\,}}\left| w_{ij}\right| ^2$ unlike (2.3) in the complex Hermitian case that also involves ${{\,\mathrm{\mathbf {E}}\,}}w_{ij}^2$. Second, uniform primitivity allows certain matrix elements of *W* to vanish. The proof under these more general assumptions follows the same strategy but requires minor modifications within the stability analysis.^1^

## Local Law

In order to directly appeal to recent results on the shape of solution to Matrix Dyson Equation (MDE) from [10] and the flexible diagrammatic cumulant expansion from [34], we first reformulate the Dyson equation (2.2) for *N*-vectors ${\mathbf {m}}$ into a matrix equation that will approximately be satisfied by the resolvent *G*. This viewpoint also allows us to treat diagonal and off-diagonal elements of *G* on the same footing. In fact, (2.2) is a special case of

3.1 $$\begin{aligned} 1+(z-A+{\mathcal {S}}[M])M=0, \end{aligned}$$

for a matrix $M=M(z) \in \mathbb {C}^{N \times N}$ with positive definite imaginary part, $\mathfrak {I}M =(M-M^*)/2\mathrm {i}>0$. The uniqueness of the solution *M* with $\mathfrak {I}M>0$ was shown in [46]. Here the linear (*self-energy*) operator ${\mathcal {S}}:\mathbb {C}^{N \times N} \rightarrow \mathbb {C}^{N \times N}$ is defined as ${\mathcal {S}}[R]:={{\,\mathrm{\mathbf {E}}\,}}WRW$ and it preserves the cone of positive definite matrices. Definition 2.1 of the scDOS and its harmonic extension $\rho (z)$ (cf. Theorem 2.5) directly generalises to the solution to (3.1), see [10, Definition 2.2].

In the special case of Wigner-type matrices the self-energy operator is given by

3.2 $$\begin{aligned} \mathcal {S}[R] = {{\,\mathrm{diag}\,}}\big (S{\mathbf {r}}\big )+T \odot R^t, \end{aligned}$$

where ${\mathbf {r}}:=(r_{ii})_{i=1}^N$, *S* was defined in (2.1), $T = (t_{ij})_{i,j=1}^N \in \mathbb {C}^{N \times N}$ with $t_{ij}={{\,\mathrm{\mathbf {E}}\,}}w_{ij}^2 \mathbb {1}(i \ne j)$ and $\odot$ denotes the entrywise Hadamard product. The solution to (3.1) is then given by $M={{\,\mathrm{diag}\,}}({\mathbf {m}})$, where ${\mathbf {m}}$ solves (2.2). Note that the action of ${\mathcal {S}}$ on diagonal matrices is independent of *T*, hence the Dyson equation (2.2) for Wigner-type matrices is solely determined by the matrix *S*, the matrix *T* plays no role. However, *T* plays a role in analyzing the error matrix *D*, see (3.4) below.

The proof of the local law consists of three largely separate arguments. The first part concerns the analysis of the stability operator

3.3 $$\begin{aligned} \mathcal {B}:=1-M\mathcal {S}[\cdot ]M \end{aligned}$$

and shape analysis of the solution *M* to (3.1). The second part is proving that the resolvent *G* is indeed an approximate solution to (3.1) in the sense that the error matrix

3.4 $$\begin{aligned} D:=1+(z-A+\mathcal {S}[G])G = WG+\mathcal {S}[G]G \end{aligned}$$

is small. In previous works [8, 11, 34] it was sufficient to establish smallness of *D* in an isotropic form $\left\langle \mathbf {x},D\mathbf {y}\right\rangle$ and averaged form $\left\langle BD\right\rangle$ with general bounded vectors/matrices $\mathbf {x},\mathbf {y},B$. In the vicinity of a cusp, however, it becomes necessary to establish an additional cancellation when *D* is averaged against the unstable direction of the stability operator $\mathcal {B}$. We call this new effect *cusp fluctuation averaging*. Finally, the third part of the proof consists of a bootstrap argument starting far away from the real axis and iteratively lowering the imaginary part $\eta =\mathfrak {I}z$ of the spectral parameter while maintaining the desired bound on $G-M$.

### Remark 3.1

We remark that the proofs of Theorem 2.5, and Corollaries 2.6 and 2.8 use the independence assumption on the entries of *W* only very locally. In fact, only the proof of a specific bound on *D* (see (3.15) later), which follows directly from the main result of the diagrammatic cumulant expansion, Theorem 3.7, uses the vector structure and the specific form of $\mathcal {S}$ in (3.2) at all. Therefore, assuming (3.15) as an input, our proof of Theorem 2.5 remains valid also in the correlated setting of [11, 34], as long as $\mathcal {S}$ is flat (see (3.6) below), and Assumption (C) is replaced by the corresponding assumption on the boundedness of $\Vert M\Vert$.

For brevity we will carry out the proof of Theorem 2.5 only in the vicinity of almost cusps as the local law in all other regimes was already proven in [8, 11] to optimality. Therefore, within this section we will always assume that $z = \tau +\mathrm {i}\eta =\tau _0+\omega +\mathrm {i}\eta \in \mathbb {H}$ lies inside a small neighbourhood

$$\begin{aligned} \mathbb {D}_{\mathrm {cusp}}:=\{z \in \mathbb {H}| \left| z-\tau _0\right| \le c\}, \end{aligned}$$

of the location $\tau _0$ of a local minimum of the scDOS within the self-consistent spectrum ${{\,\mathrm{supp}\,}}\rho$. Here *c* is a sufficiently small constant depending only on the model parameters. We will further assume that either (i) $\rho (\tau _0)\ge 0$ is sufficiently small and $\tau _0$ is the location of a cusp or internal minimum, or (ii) $\rho (\tau _0)=0$ and $\tau _0$ is an edge adjacent to a sufficiently small gap of length $\Delta >0$. The results from [10] guarantee that these are the only possibilities for the shape of $\rho$, see (2.4). In other words, we assume that $\tau _0 \in {{\,\mathrm{supp}\,}}\rho$ is a local minimum of $\rho$ with a shape close to a cusp (cf. (2.4)). For concreteness we will also assume that if $\tau _0$ is an edge, then it is a right edge (with a gap of length $\Delta >0$ to the right) and $\omega \in (-c, \frac{\Delta }{2}]$. The case when $\tau _0$ is a left edge has the same proof.

We now introduce a quantity that will play an important role in the cusp fluctuation averaging mechanism. We define 3.5awhere $\mathfrak {R}M:=(M+M^*)/2$ is the real part of $M=M(z)$. It was proven in [10, Lemma 5.5] that $\sigma (z)$ extends to the real line as a 1/3-Hölder continuous function wherever the scDOS $\rho$ is smaller than some threshold $c\sim 1$, i.e. $\rho \le c$. In the specific case of $\mathcal {S}$ as in (3.2) the definition simplifies to

3.5b $$\begin{aligned} \sigma (z):=\left\langle {\mathbf {p}}{\mathbf {f}}^3\right\rangle = \frac{1}{N}\sum _{i=1}^N \frac{(\mathfrak {I}m_i(z))^3{{\,\mathrm{sgn}\,}}\mathfrak {R}m_i(z)}{\rho (z)^3\left| m_i(z)\right| ^3},\qquad {\mathbf {f}}:=\frac{\mathfrak {I}{\mathbf {m}}}{\rho \left| {\mathbf {m}}\right| },\qquad {\mathbf {p}}:={{\,\mathrm{sgn}\,}}\mathfrak {R}{\mathbf {m}},\nonumber \\ \end{aligned}$$

since $M={{\,\mathrm{diag}\,}}({\mathbf {m}})$ is diagonal, where multiplication and division of vectors are understood entrywise. When evaluated at the location $\tau _0$ the scalar $\sigma (\tau _0)$ provides a measure of how far the shape of the singularity at $\tau _0$ is from an exact cusp. In fact, if $\sigma (\tau _0)=0$ and $\rho (\tau _0)=0$, then $\tau _0$ is a cusp location. To see the relationship between the emergence of a cusp and the limit $\sigma (\tau _0) \rightarrow 0$, we refer to [10, Theorem 7.7 and Lemma 6.3]. The analogues of the quantities ${\mathbf {f}},{\mathbf {p}}$ and $\sigma$ in (3.5b) are denoted by $f_u,s$ and $\sigma$ in [10], respectively. The significance of $\sigma$ for the classification of singularity types in Wigner-type ensembles was first realised in [5]. Although in this paper we will use only [10] and will not rely on [5], we remark that the definition of $\sigma$ in [5, Eq. (8.11)] differs slightly from the definition (3.5b). However, both definitions equally fulfil the purpose of classifying singularity types, since the ensuing scalar quantities $\sigma$ are comparable inside the self-consistent spectrum. For the interested reader, we briefly relate our notations to the respective conventions in [10] and [5]. The quantity denoted by *f* in both [10] and [5] is the normalized eigendirection of the *saturated self-energy operator* *F* in the respective settings and is related to ${\mathbf {f}}$ from (3.5b) via $f={\mathbf {f}}/ \Vert {\mathbf {f}}\Vert +{\mathcal {O}}\,\left( \eta /\rho \right)$. Moreover, $\sigma$ in [5] is defined as $\left\langle f^3 {{\,\mathrm{sgn}\,}}\mathfrak {R}{\mathbf {m}}\right\rangle$, justifying the comparability to $\sigma$ from (3.5b).

### Stability and shape analysis

From (3.1) and (3.4) we obtain the quadratic *stability equation*

$$\begin{aligned} {\mathcal {B}}[G-M]= -MD+M{\mathcal {S}}[G-M](G-M), \end{aligned}$$

for the difference $G-M$. In order to apply the results of [10] to the stability operator $\mathcal {B}$, we first have to check that the flatness condition [10, Eq. (3.10)] is satisfied for the self-energy operator $\mathcal {S}$. We claim that $\mathcal {S}$ is flat, i.e.

3.6 $$\begin{aligned} {\mathcal {S}}[R] \sim \left\langle R\right\rangle 1= \frac{1}{N} ({{\,\mathrm{Tr}\,}}R) 1, \end{aligned}$$

as quadratic forms for any positive semidefinite $R \in \mathbb {C}^{N \times N}$. We remark that in the earlier paper [8] in the Wigner-type case only the upper bound $s_{ij}\le C/N$ defined the concept of flatness. Here with the definition (3.6) we follow the convention of the more recent works [10, 11, 34] which is more conceptual. We also warn the reader, that in the complex Hermitian Wigner-type case the condition $c/N\le s_{ij}\le C/N$ implies (3.6) only if $t_{ij}$ is bounded away from $-s_{ij}$.

However, the flatness (3.6) is an immediate consequence of the fullness Assumption (B). Indeed, (B) is equivalent to the condition that the covariance operator $\Sigma$ of all entries above and on the diagonal, defined as $\Sigma _{ab,cd}:={{\,\mathrm{\mathbf {E}}\,}}w_{ab} w_{cd}$, is uniformly strictly positive definite. This implies that $\Sigma \ge c \Sigma _{\mathrm {G}}$ for some constant $c\sim 1$, where $\Sigma _{\mathrm {G}}$ is the covariance operator of a GUE or GOE matrix, depending on the symmetry class we consider. This means that ${\mathcal {S}}$ can be split into ${\mathcal {S}}={\mathcal {S}}_0+c {\mathcal {S}}_{\mathrm {G}}$, where ${\mathcal {S}}_\mathrm {G}$ and $\mathcal {S}_0$ are the self-energy operators corresponding to $\Sigma _\mathrm {G}$ and $\Sigma -c\Sigma _\mathrm {G}$, respectively. It is now an easy exercise to check that ${\mathcal {S}}_{\mathrm {G}}$ and thus ${\mathcal {S}}$ is flat.

In particular, [10, Proposition 3.5 and Lemma 4.8] are applicable implying that [10, Assumption 4.5] is satisfied. Thus, according to [10, Lemma 5.1] for spectral parameters *z* in a neighbourhood of $\tau _0$ the operator ${\mathcal {B}}$ has a unique isolated eigenvalue $\beta$ of smallest modulus and associated right $\mathcal {B}[V_\mathrm {r}]=\beta V_\mathrm {r}$ and left ${\mathcal {B}}^*[V_\mathrm {l}]= \overline{\beta } V_{\mathrm {l}}$ eigendirections normalised such that $\Vert V_\mathrm {r}\Vert _{\mathrm {hs}} =\langle {V_\mathrm {l}} \,, {V_\mathrm {r}}\rangle =1$. We denote the spectral projections to $V_\mathrm {r}$ and to its complement by $\mathcal {P}:=\left\langle V_\mathrm {l},\cdot \right\rangle V_\mathrm {r}$ and $\mathcal {Q}:=1-\mathcal {P}$. For convenience of the reader we now collect some important quantitative information about the stability operator and its unstable direction from [10].

#### Proposition 3.2

(Properties of the MDE and its solution). The following statements hold true uniformly in $z=\tau _0+\omega +\mathrm {i}\eta \in \mathbb {D}_\mathrm {cusp}$ assuming flatness as in (3.6) and the uniform boundedness of $\Vert M\Vert$ for $z\in \tau _0+(-\kappa ,\kappa )+\mathrm {i}\mathbb {R}_+$,

(i) The eigendirections $V_\mathrm {l},V_\mathrm {r}$ are norm-bounded and the operator $\mathcal {B}^{-1}$ is bounded on the complement to its unstable direction, i.e.
  3.7a $$\begin{aligned} \Vert \mathcal {B}^{-1}\mathcal {Q}\Vert _{\mathrm {hs}\rightarrow \mathrm {hs}}+\Vert V_\mathrm {r}\Vert +\Vert V_\mathrm {l}\Vert \lesssim 1.\end{aligned}$$
(ii) The density $\rho$ is comparable with the explicit function $\rho (\tau _0+\omega +\mathrm {i}\eta )\sim \widetilde{\rho }(\tau _0+\omega +\mathrm {i}\eta )$ given by
  3.7b $$\begin{aligned} \widetilde{\rho } :={\left\{ \begin{array}{ll} \rho (\tau _0)+(\left| \omega \right| +\eta )^{1/3},&{}\text {in cases (i),(iii) if }\tau _0=\mathfrak {m},\mathfrak {c},\\ (\left| \omega \right| +\eta )^{1/2}(\Delta +\left| \omega \right| +\eta )^{-1/6},&{}\text {in case (ii) if }\tau _0=\mathfrak {e}_-,\; \omega \in [-c,0]\\ \eta (\Delta +\left| \omega \right| +\eta )^{-1/6}(\left| \omega \right| +\eta )^{-1/2},&{}\text {in case (ii) if }\tau _0=\mathfrak {e}_-,\; \omega \in [0,\Delta /2].\\ \end{array}\right. }\nonumber \\ \end{aligned}$$
(iii) The eigenvalue $\beta$ of smallest modulus satisfies
  3.7c $$\begin{aligned} \left| \beta \right| \sim \frac{\eta }{\rho } + \rho (\rho +\left| \sigma \right| ), \end{aligned}$$
  and we have the comparison relations
  3.7d $$\begin{aligned} \begin{aligned}&\left| \left\langle V_\mathrm {l}, M \mathcal {S}[V_\mathrm {r}]V_\mathrm {r}\right\rangle \right| \sim \rho +\left| \sigma \right| , \\&\left| \left\langle V_\mathrm {l},M\mathcal {S}[V_\mathrm {r}]\mathcal {B}^{-1}\mathcal {Q}[M\mathcal {S}[V_\mathrm {r}]V_\mathrm {r}]+M\mathcal {S}\mathcal {B}^{-1}\mathcal {Q}[M\mathcal {S}[V_\mathrm {r}]V_\mathrm {r}]V_\mathrm {r}\right\rangle \right| \sim 1. \end{aligned} \end{aligned}$$
(iv) The quantities $\eta /\rho +\rho (\rho +\left| \sigma \right| )$ and $\rho +\left| \sigma \right|$ in (3.7c)–(3.7d) can be replaced by the following more explicit auxiliary quantities
  3.7e $$\begin{aligned} \begin{aligned} \widetilde{\xi }_1(\tau _0+\omega +\mathrm {i}\eta )&:={\left\{ \begin{array}{ll} (\left| \omega \right| +\eta )^{1/2} (\left| \omega \right| +\eta +\Delta )^{1/6},\\ (\rho (\tau _0)+(\left| \omega \right| +\eta )^{1/3})^2, \end{array}\right. }\\ \widetilde{\xi }_2(\tau _0+\omega +\mathrm {i}\eta )&:={\left\{ \begin{array}{ll} (\left| \omega \right| +\eta +\Delta )^{1/3}, &{}\text {if } \tau _0=\mathfrak {e}_-,\\ \rho (\tau _0)+(\left| \omega \right| +\eta )^{1/3}, &{}\text {if }\tau _0=\mathfrak {m},\mathfrak {c}. \end{array}\right. } \end{aligned} \end{aligned}$$
  which are monotonically increasing in $\eta$. More precisely, it holds that $\eta /\rho + \rho (\rho +\left| \sigma \right| ) \sim \widetilde{\xi }_1$ and, in the case where $\tau _0=\mathfrak {c},\mathfrak {m}$ is a cusp or a non-zero local minimum, we also have that $\rho +\left| \sigma \right| \sim \widetilde{\xi }_2$. For the case when $\tau _0=\mathfrak {e}_-$ is a right edge next to a gap of size $\Delta$ there exists a constant $c_*$ such that $\rho +\left| \sigma \right| \sim \widetilde{\xi }_2$ in the regime $\omega \in [-c,c_*\Delta ]$ and $\rho +\left| \sigma \right| \lesssim \widetilde{\xi }_2$ in the regime $\omega \in [c_*\Delta ,\Delta /2]$.

#### Proof

We first explain how to translate the notations from the present paper to the notations in [10]: The operators $\mathcal {S},\mathcal {B},\mathcal {Q}$ are simply denoted by *S*, *B*, *Q* in [10]; the matrices $V_l,V_r$ here are denoted by $l/\langle {l} \,, {b}\rangle ,b$ there. The bound on $\mathcal {B}^{-1}\mathcal {Q}$ in (3.7a) follows directly from [10, Eq. (5.15)]. The bounds on $V_\mathrm {l},V_\mathrm {r}$ in (3.7a) follow from the definition of the stability operator (3.3) together with the fact that $\Vert M\Vert \lesssim 1$ (by Assumption (C)) and $\Vert {\mathcal {S}}\Vert _{\mathrm {hs} \rightarrow \Vert \cdot \Vert } \lesssim 1$, following from the upper bound in flatness (3.6). The asymptotic expansion of $\rho$ in (3.7b) follows from [10, Remark 7.3] and [5, Corollary A.1]. The claims in (iii) follow directly from [10, Proposition 6.1]. Finally, the claims in (iv) follow directly from [10, Remark 10.4]. $\square$

The following lemma establishes simplified lower bounds on $\widetilde{\xi }_1,\widetilde{\xi }_2$ whenever $\eta$ is much larger than the fluctuation scale $\eta _\mathrm {f}$. We defer the proof of the technical lemma which differentiates various regimes to the Appendix.

#### Lemma 3.3

Under the assumptions of Proposition 3.2 we have uniformly in $z=\tau _0+\omega +\mathrm {i}\eta \in \mathbb {D}_\mathrm {cusp}$ with $\eta \ge \eta _\mathrm {f}$ that

$$\begin{aligned} \widetilde{\xi }_2\gtrsim \frac{1}{N\eta }+\Bigl (\frac{\rho }{N\eta }\Bigr )^{1/2}, \qquad \widetilde{\xi }_1\gtrsim \widetilde{\xi }_2\Bigl (\rho +\frac{1}{N\eta }\Bigr ). \end{aligned}$$

We now define an appropriate matrix norm in which we will measure the distance between *G* and *M*. The $\Vert \cdot \Vert _*$-norm is defined exactly as in [11] and similar to the one first introduced in [34]. It is a norm comparing matrix elements on a large but finite set of vectors with a hierarchical structure. To define this set we introduce some notations. For second order cumulants of matrix elements $\kappa (w_{ab},w_{cd}):={{\,\mathrm{\mathbf {E}}\,}}w_{ab}w_{cd}$ we use the short-hand notation $\kappa (ab,cd)$. We also use the short-hand notation $\kappa (\mathbf {x}b,cd)$ for the $\mathbf {x}=(x_a)_{a\in [N]}$-weighted linear combination $\sum _a x_a \kappa (ab,cd)$ of such cumulants. We use the notation that replacing an index in a scalar quantity by a dot ($\cdot$) refers to the corresponding vector, e.g. $A_{a\cdot }$ is a short-hand notation for the vector $(A_{ab})_{b\in [N]}$. Matrices $R_{\mathbf {x}\mathbf {y}}$ with vector subscripts $\mathbf {x},\mathbf {y}$ are understood as short-hand notations for $\left\langle \mathbf {x},R\mathbf {y}\right\rangle$, and matrices $R_{\mathbf {x}a}$ with mixed vector and index subscripts are understood as $\left\langle \mathbf {x},R e_a\right\rangle$ with $e_a$ being the *a*th normalized $\Vert e_a\Vert =1$ standard basis vector. We fix two vectors $\mathbf {x},\mathbf {y}$ and some large integer *K* and define the sets of vectors

$$\begin{aligned} \begin{aligned} I_0&:=\{\mathbf {x},\mathbf {y}\} \cup \{\delta _{a\cdot }, (V_\mathrm {l}^*)_{a\cdot }| a \in [N]\}, \\ I_{k+1}&:=I_k\cup \{M{{\mathbf {u}}}|{{\mathbf {u}}}\in I_k\}\cup \{\kappa _\mathrm {c}((M{{\mathbf {u}}})a,b\cdot ),\kappa _\mathrm {d}((M{{\mathbf {u}}})a,\cdot b)|{{\mathbf {u}}}\in I_k,a,b\in [N]\}. \end{aligned} \end{aligned}$$

Here the cross and the direct part $\kappa _\mathrm {c},\kappa _\mathrm {d}$ of the 2-cumulants $\kappa (\cdot ,\cdot )$ refer to the natural splitting dictated by the Hermitian symmetry. In the specific case of (3.2) we simply have $\kappa _\mathrm {c}(ab,cd)=\delta _{ad}\delta _{bc}s_{ab}$ and $\kappa _\mathrm {d}(ab,cd)=\delta _{ac}\delta _{bd}t_{ab}$. Then the $\Vert \cdot \Vert _*$-norm is given by

$$\begin{aligned} \Vert R\Vert _*= \Vert R\Vert _*^{K,{\mathbf {x}},{\mathbf {y}}} :=\sum _{0\le k< K}N^{-k/2K} \Vert R\Vert _{I_k} + N^{-1/2} \max _{{{\mathbf {u}}}\in I_K}\frac{\Vert R_{\cdot {{\mathbf {u}}}}\Vert }{\Vert {{\mathbf {u}}}\Vert },\qquad \Vert R\Vert _I :=\max _{{{\mathbf {u}}},{\mathbf {v}}\in I} \frac{\left| R_{{{\mathbf {u}}}{\mathbf {v}}}\right| }{\Vert {{\mathbf {u}}}\Vert \Vert {\mathbf {v}}\Vert }. \end{aligned}$$

We remark that the set $I_k$ hence also $\Vert \cdot \Vert _*$ depend on *z* via $M=M(z)$. We omit this dependence from the notation as it plays no role in the estimates.

In terms of this norm we obtain the following estimate on $G-M$ in terms of its projection $\Theta =\left\langle V_\mathrm {l},G-M\right\rangle$ onto the unstable direction of the stability operator $\mathcal {B}$. It is a direct consequence of a general expansion of approximate quadratic matrix equations whose linear stability operators have a single eigenvalue close to 0, as given in Lemma A.1.

#### Proposition 3.4

(Cubic equation for $\Theta$). Fix $K\in \mathbb {N}$, $\mathbf {x},\mathbf {y}\in \mathbb {C}^N$ and use $\Vert \cdot \Vert _*=\Vert \cdot \Vert _*^{K,\mathbf {x},\mathbf {y}}$. For fixed $z \in \mathbb {D}_{\mathrm {cusp}}$ and on the event that $\Vert G-M\Vert _*+\Vert D\Vert _*\lesssim N^{-10/K}$ the difference $G-M$ admits the expansion

3.8a $$\begin{aligned} \begin{aligned} G-M&= \Theta V_\mathrm {r} -{\mathcal {B}}^{-1}{\mathcal {Q}}[MD]+\Theta ^2{\mathcal {B}}^{-1}{\mathcal {Q}}[M{\mathcal {S}}[V_\mathrm {r}]V_\mathrm {r}] + E,\\ \Vert E\Vert _*&\lesssim N^{5/K}(\left| \Theta \right| ^3 +\left| \Theta \right| \Vert D\Vert _*+ \Vert D\Vert _*^2), \end{aligned} \end{aligned}$$

with an error matrix *E* and the scalar $\Theta :=\left\langle V_\mathrm {l}, G-M\right\rangle$ that satisfies the approximate cubic equation

3.8b $$\begin{aligned} \Theta ^3+ \xi _2 \Theta ^2 +\xi _1 \Theta = \epsilon _*. \end{aligned}$$

Here, the error $\epsilon _*$ satisfies the upper bound

3.8c $$\begin{aligned}&\left| \epsilon _*\right| \lesssim N^{20/K}(\Vert D\Vert _*^3+\left| \left\langle R, D\right\rangle \right| ^{3/2})+ \left| \left\langle V_\mathrm {l}, MD\right\rangle \right| \nonumber \\&\quad + \left| \left\langle V_\mathrm {l},M({\mathcal {S}}{\mathcal {B}}^{-1}{\mathcal {Q}}[MD])({\mathcal {B}}^{-1}{\mathcal {Q}}[MD])\right\rangle \right| , \end{aligned}$$

where *R* is a deterministic matrix with $\Vert R\Vert \lesssim 1$ and the coefficients of the cubic equation satisfy the comparison relations

3.8d $$\begin{aligned} \left| \xi _1\right| \sim \frac{\eta }{\rho }+\rho (\rho +\left| \sigma \right| ),\qquad \left| \xi _2\right| \sim \rho +\left| \sigma \right| . \end{aligned}$$

#### Proof

We first establish some important bounds involving the $\Vert \cdot \Vert _*$-norm. We claim that for any matrices $R,R_1,R_2$

3.9 $$\begin{aligned} \begin{aligned}&\Vert M{\mathcal {S}}[R_1]R_2\Vert _*\lesssim N^{1/2K}\Vert R_1\Vert _*\Vert R_2\Vert _*,\quad \Vert MR\Vert _*\lesssim N^{1/2K}\Vert R\Vert _*,\\&\Vert {\mathcal {Q}}\Vert _{*\rightarrow *}\lesssim 1,\quad \Vert {\mathcal {B}}^{-1}{\mathcal {Q}}\Vert _{*\rightarrow *}\lesssim 1,\quad \left| \left\langle V_\mathrm {l}, R\right\rangle \right| \lesssim \Vert R\Vert _*. \end{aligned} \end{aligned}$$

The proof of (3.9) follows verbatim as in [11, Lemma 3.4] with (3.7a) as an input. Moreover, the bound on $\left\langle V_\mathrm {l}, \cdot \right\rangle$ follows directly from the bound on ${\mathcal {Q}}$. Obviously, we also have $\Vert \cdot \Vert _*\le 2 \Vert \cdot \Vert$.

Next, we apply Lemma A.1 from the Appendix with the choices

$$\begin{aligned} {\mathcal {A}}[R_1,R_2]:=M{\mathcal {S}}[R_1]R_2,\qquad {X}:=MD, \qquad Y:=G-M. \end{aligned}$$

The operator ${\mathcal {B}}$ in Lemma A.1 is chosen as the stability operator (3.3). Then (A.1) is satisfied with $\lambda :=N^{1/2K}$ according to (3.9) and (3.7a). With $\delta :=N^{-25/4K}$ we verify (3.8a) directly from (A.5), where $\Theta = \left\langle V_\mathrm {l}, G-M\right\rangle$ satisfies

3.10 $$\begin{aligned} \mu _3 \Theta ^3+\mu _2 \Theta ^2 -\beta \Theta = -\mu _0 + \left\langle R, D\right\rangle \Theta +{\mathcal {O}}\,\left( N^{-1/4K} \left| \Theta \right| ^3 + N^{20/K}\Vert D\Vert _*^3\right) .\nonumber \\ \end{aligned}$$

Here we used $\left| \Theta \right| \le \Vert G-M\Vert _*\lesssim N^{-10/K}$ and $\Vert MD\Vert _*\lesssim N^{1/2K}\Vert D\Vert _*$. The coefficients $\mu _0, \mu _2, \mu _3$ are defined through (A.4) and *R* is given by

$$\begin{aligned} R :=M^*({\mathcal {B}}^{-1}{\mathcal {Q}})^*[{\mathcal {S}}[M^*V_\mathrm {l} V_\mathrm {r}^*]+{\mathcal {S}}[V_\mathrm {r}^*]M^*V_\mathrm {l}]. \end{aligned}$$

Now we bound $\left| \left\langle R, D\right\rangle \Theta \right| \le N^{-1/4K} \left| \Theta \right| ^3 + N^{1/8K} \left| \left\langle R, D\right\rangle \right| ^{3/2}$ by Young’s inequality, absorb the error terms bounded by  into the cubic term, $\mu _3 \Theta ^3 + \mathcal {O}(N^{-1/4K} \left| \Theta \right| ^3) = \widetilde{\mu }_3 \Theta ^3$, by introducing a modified coefficient $\widetilde{\mu }_3$ and use that $\left| \mu _3\right| \sim \left| \widetilde{\mu }_3\right| \sim 1$ for any $z \in \mathbb {D}_{\mathrm {cusp}}$. Finally, we safely divide (3.10) by $\widetilde{\mu }_3$ to verify (3.8b) with $\xi _1:=-\beta / \widetilde{\mu }_3$ and $\xi _2 :=\mu _2 / \widetilde{\mu }_3$. For the fact $\left| \mu _3\right| \sim 1$ on $\mathbb {D}_{\mathrm {cusp}}$ and the comparison relations (3.8d) we refer to (3.7c)–(3.7d). $\square$

### Probabilistic bound

We now collect bounds on the error matrix *D* from [34, Theorem 4.1] and Sect. 4. We first introduce the notion of *stochastic domination*.

#### Definition 3.5

*(Stochastic domination).* Let $X=X^{(N)}, Y=Y^{(N)}$ be sequences of non-negative random variables. We say that *X* is stochastically dominated by *Y* (and use the notation $X \prec Y$) if

$$\begin{aligned} \mathbf {P}\big [X > N^\epsilon Y\big ]\le C(\epsilon ,\nu )N^{-\nu },\qquad N \in \mathbb {N}, \end{aligned}$$

for any $\epsilon >0, \nu \in \mathbb {N}$ and some family of positive constants $C(\epsilon ,\nu )$ that is uniform in *N* and other underlying parameters (e.g. the spectral parameter *z* in the domain under consideration).

It can be checked (see [33, Lemma 4.4]) that $\prec$ satisfies the usual arithmetic properties, e.g. if $X_1\prec Y_1$ and $X_2\prec Y_2$, then also $X_1+X_2\prec Y_1 +Y_2$ and $X_1X_2\prec Y_1 Y_2$. Furthermore, to formulate bounds on a random matrix *R* compactly, we introduce the notationsfor random matrices *R* and a deterministic control parameter $\Lambda =\Lambda (z)$. We also introduce high moment norms

$$\begin{aligned} \Vert X\Vert _p:=\Big ({{\,\mathrm{\mathbf {E}}\,}}\left| X\right| ^p\Big )^{1/p},\qquad \Vert R\Vert _p :=\sup _{\mathbf {x},\mathbf {y}} \frac{\Vert \left\langle \mathbf {x}, R\mathbf {y}\right\rangle \Vert _p}{\Vert \mathbf {x}\Vert \Vert \mathbf {y}\Vert } \end{aligned}$$

for $p\ge 1$, scalar valued random variables *X* and random matrices *R*. To translate high moment bounds into high probability bounds and vice versa we have the following easy lemma [11, Lemma 3.7].

#### Lemma 3.6

Let *R* be a random matrix, $\Phi$ a deterministic control parameter such that $\Phi \ge N^{-C}$ and $\Vert R\Vert \le N^C$ for some $C>0$, and let $K\in \mathbb {N}$ be a fixed integer. Then we have the equivalences

$$\begin{aligned} \Vert R\Vert _*^{K,\mathbf {x},\mathbf {y}}&\prec \Phi \text { uniformly in }\mathbf {x},\mathbf {y}\quad \Longleftrightarrow \quad \left| R\right| \prec \Phi \quad \Longleftrightarrow \quad \Vert R\Vert _p\le _{p,\epsilon }\\&\quad N^{\epsilon }\Phi \text { for all }\epsilon >0,\,p\ge 1. \end{aligned}$$

Expressed in terms of the $\Vert \cdot \Vert _p$-norm we have the following high-moment bounds on the error matrix *D*. The bounds (3.11a)–(3.11b) have already been established in [34, Theorem 4.1]; we just list them for completeness. The bounds (3.11c)–(3.11d), however, are new and they capture the additional cancellation at the cusp and are the core novelty of the present paper. The additional smallness comes from averaging against specific weights ${\mathbf {p}},{\mathbf {f}}$ from (3.5b).

#### Theorem 3.7

(High moment bound on *D* with cusp fluctuation averaging). Under the assumptions of Theorem 2.5 for any compact set $\mathbb {D}\subset \{z\in \mathbb {C}|\mathfrak {I}z\ge N^{-1}\}$ there exists a constant *C* such that for any $p\ge 1,\epsilon >0$, $z\in \mathbb {D}$ and matrices/vectors $B,\mathbf {x},\mathbf {y}$ it holds that

3.11a $$\begin{aligned}&\Vert \left\langle \mathbf {x},D\mathbf {y}\right\rangle \Vert _p \le _{\epsilon ,p} \Vert \mathbf {x}\Vert \Vert \mathbf {y}\Vert N^{\epsilon } \psi _q'\Big (1+\Vert G\Vert _q \Big )^C \bigg (1+\frac{\Vert G\Vert _q}{\sqrt{N}}\bigg )^{Cp}, \end{aligned}$$

3.11b $$\begin{aligned}&\Vert \left\langle BD\right\rangle \Vert _p \le _{\epsilon ,p} \Vert B\Vert N^{\epsilon } \Big [\psi _q'\Big ]^2\Big (1+\Vert G\Vert _q \Big )^C \bigg (1+\frac{\Vert G\Vert _q}{\sqrt{N}}\bigg )^{Cp}. \end{aligned}$$

Moreover, for the specific weight matrix $B={{\,\mathrm{diag}\,}}({\mathbf {p}}{\mathbf {f}})$ we have the improved bound

3.11c $$\begin{aligned} \Vert \left\langle {{\,\mathrm{diag}\,}}({\mathbf {p}}{\mathbf {f}})D\right\rangle \Vert _p&\le _{\epsilon ,p}N^{\epsilon } \sigma _q \Big [ \psi +\psi '_q \Big ]^2 \Big (1+\Vert G\Vert _q \Big )^C \bigg (1+\frac{\Vert G\Vert _q}{\sqrt{N}}\bigg )^{Cp}, \end{aligned}$$

and the improved bound on the off-diagonal component

3.11d $$\begin{aligned} \Vert \left\langle {{\,\mathrm{diag}\,}}({\mathbf {p}}{\mathbf {f}}) [T\odot G^t]G\right\rangle \Vert _p&\le _{\epsilon ,p} N^{\epsilon }\sigma _q \Big [ \psi +\psi '_q \Big ]^2 \Big (1+\Vert G\Vert _q\Big )^C\bigg (1+\frac{\Vert G\Vert _q}{\sqrt{N}}\bigg )^{Cp},\nonumber \\ \end{aligned}$$

where we defined the following *z*-dependent quantities

$$\begin{aligned} \psi :=\sqrt{\frac{\rho }{N\eta }},\quad \psi '_q :=\sqrt{\frac{\Vert \mathfrak {I}G\Vert _q}{N\eta }},\quad \psi ''_q :=\Vert G-M\Vert _q,\quad \sigma _q:=\left| \sigma \right| +\rho +\psi +\sqrt{\eta /\rho }+\psi _q'+\psi _q'' \end{aligned}$$

and $$q=Cp^3/\epsilon $$.

Theorem 3.7 will be proved in Sect. 4. We now translate the high moment bounds of Theorem 3.7 into high probability bounds via Lemma 3.6 and use those to establish bounds on $G-M$ and the error in the cubic equation for $\Theta$. To simplify the expressions we formulate the bounds in the domain

3.12 $$\begin{aligned} \mathbb {D}_\zeta :=\{z \in \mathbb {D}_\mathrm {cusp}| \mathfrak {I}z \ge N^{-1+\zeta }\}. \end{aligned}$$

#### Lemma 3.8

(High probability error bounds). Fix $\zeta ,c>0$ sufficiently small and suppose that $\left| G-M\right| \prec \Lambda$, $\left| \mathfrak {I}(G-M)\right| \prec \Xi$ and $\left| \Theta \right| \prec \theta$ hold at fixed $z \in \mathbb {D}_\zeta$, and assume that the deterministic control parameters $\Lambda , \Xi ,\theta$ satisfy $\Lambda +\Xi +\theta \lesssim N^{-c}$. Then for any sufficiently small $\epsilon >0$ it holds that

3.13a $$\begin{aligned} \left| \Theta ^3+ \xi _2 \Theta ^2 +\xi _1 \Theta \right| \prec N^{2\epsilon }\bigg (\rho + \left| \sigma \right| +\frac{\eta ^{1/2}}{\rho ^{1/2}}+\bigg (\frac{\rho +\Xi }{N \eta }\bigg )^{1/2}\bigg ) \frac{\rho +\Xi }{N \eta } +N^{-\epsilon }\theta ^3,\nonumber \\ \end{aligned}$$

as well as

3.13b $$\begin{aligned} \left| G-M\right| \prec \theta + \sqrt{\frac{\rho +\Xi }{N \eta }},\qquad \left| G-M\right| _{\mathrm {av}}\prec \theta + {\frac{\rho +\Xi }{N \eta }}, \end{aligned}$$

where the coefficients $\xi _1,\xi _2$ are those from Proposition 3.4, and we recall that $\Theta =\left\langle V_l,G-M\right\rangle$.

#### Proof

We translate the high moment bounds (3.11a)–(3.11b) into high probability bounds using Lemma 3.6 and $\left| G\right| \prec \Vert M\Vert + \Lambda \lesssim 1$ to find

3.14 $$\begin{aligned} \left| D\right| \prec \sqrt{\frac{\rho +\Xi }{N \eta }},\qquad \left| D\right| _{\mathrm {av}}\prec {\frac{\rho +\Xi }{N \eta }}. \end{aligned}$$

In particular, these bounds together with the assumed bounds on $G-M$ guarantee the applicability of Proposition 3.4. Now we use (3.14) and (3.9) in (3.8a) to get (3.13b). Here we used (3.9), translated $\Vert \cdot \Vert _p$-bounds into $\prec$-bounds on $\Vert \cdot \Vert _*$ and vice versa via Lemma 3.6, and absorbed the $N^{1/K}$ factors into $\prec$ by using that *K* can be chosen arbitrarily large. It remains to verify (3.13a). In order to do so, we first claim that

3.15 $$\begin{aligned} \begin{aligned}&\left| \left\langle V_\mathrm {l}, MD\right\rangle \right| + \left| \left\langle V_\mathrm {l},M({\mathcal {S}}{\mathcal {B}}^{-1}{\mathcal {Q}}[MD])({\mathcal {B}}^{-1}{\mathcal {Q}}[MD])\right\rangle \right| \\&\prec N^\epsilon \bigg (\left| \sigma \right| +\rho +{\frac{\eta ^{1/2}}{\rho ^{1/2}}}+ \Lambda +\bigg (\frac{\rho +\Xi }{N \eta }\bigg )^{1/2}\bigg )\frac{\rho +\Xi }{N \eta } +\theta ^2\bigg (N^{-\epsilon }\Lambda +\bigg (\frac{\rho +\Xi }{N \eta }\bigg )^{1/2}\bigg ) \end{aligned}\nonumber \\ \end{aligned}$$

for any sufficiently small $\epsilon >0$.

#### Proof of (3.15)

We first collect two additional ingredients from [10] specific to the vector case.

- The imaginary part $\mathfrak {I}{\mathbf {m}}$ of the solution ${\mathbf {m}}$ is comparable $\mathfrak {I}{\mathbf {m}}\sim \left\langle \mathfrak {I}{\mathbf {m}}\right\rangle =\pi \rho$ to its average in the sense $c \left\langle \mathfrak {I}{\mathbf {m}}\right\rangle \le \mathfrak {I}m_i\le C \left\langle \mathfrak {I}{\mathbf {m}}\right\rangle$ for all $i$ and some $c,C>0$, and, in particular, ${\mathbf {m}}=\mathfrak {R}{\mathbf {m}}+{\mathcal {O}}\,\left( \rho \right)$.
- The eigendirections $V_\mathrm {l},V_\mathrm {r}$ are diagonal and are approximately given by
  3.16 $$\begin{aligned} V_\mathrm {l} = c{{\,\mathrm{diag}\,}}({\mathbf {f}}/\left| {\mathbf {m}}\right| ) + {\mathcal {O}}\,\left( \rho +\eta /\rho \right) ,\qquad V_\mathrm {r}=c' {{\,\mathrm{diag}\,}}({\mathbf {f}}\left| {\mathbf {m}}\right| )+ {\mathcal {O}}\,\left( \rho +\eta /\rho \right) \nonumber \\ \end{aligned}$$
  for some constants $c,c'\sim 1$.

Indeed, (a) follows directly from [10, Proposition 3.5] and the approximations in (3.16) follow directly from [10, Corollary 5.2]. The fact that $V_\mathrm {l},V_\mathrm {r}$ are diagonal follows from simplicity of the eigendirections in the matrix case, and the fact that $M={{\,\mathrm{diag}\,}}({\mathbf {m}})$ is diagonal and that ${\mathcal {B}}$ preserves the space of diagonal matrices as well as the space of off-diagonal matrices. On the latter ${\mathcal {B}}$ acts stably as $1+\mathcal {O}_{\mathrm {hs}\rightarrow \mathrm {hs}}(N^{-1})$. Thus the unstable directions lie inside the space of diagonal matrices.

We now turn to the proof of (3.15) and first note that, according to (a) and (b) we have

3.17 $$\begin{aligned} M= {{\,\mathrm{diag}\,}}({\mathbf {p}}\left| {\mathbf {m}}\right| )+{\mathcal {O}}\,\left( \rho \right) , \qquad V_\mathrm {l} = c{{\,\mathrm{diag}\,}}({\mathbf {f}}/\left| {\mathbf {m}}\right| )+{\mathcal {O}}\,\left( \rho +\eta /\rho \right) \end{aligned}$$

with errors in $\Vert \cdot \Vert$-norm-sense, for some constant $c \sim 1$ to see

$$\begin{aligned} \left\langle V_\mathrm {l}, MD\right\rangle = c\left\langle {{\,\mathrm{diag}\,}}({\mathbf {p}}{\mathbf {f}})D\right\rangle + {\mathcal {O}}\,\left( \rho +\eta /\rho \right) \left\langle {{\,\mathrm{diag}\,}}({\mathbf {w}}_1)D\right\rangle , \end{aligned}$$

where ${\mathbf {w}}_1\in \mathbb {C}^N$ is a deterministic vector with uniformly bounded entries. Since $\left| \left\langle {{\,\mathrm{diag}\,}}({\mathbf {w}}_1)D\right\rangle \right| \prec (\rho +\Xi )/N\eta$ by (3.14), the bound on the first term in (3.15) follows together with (3.11c) via Lemma 3.6. Now we consider the second term in (3.15). We split $D = D_\mathrm {d} + D_\mathrm {o}$ into its diagonal and off-diagonal components. Since ${\mathcal {B}}$ and ${\mathcal {S}}$ preserve the space of diagonal and the space of off-diagonal matrices we find

3.18 $$\begin{aligned} \begin{aligned}&\left\langle V_\mathrm {l},M({\mathcal {S}}{\mathcal {B}}^{-1}{\mathcal {Q}}[MD])({\mathcal {B}}^{-1}{\mathcal {Q}}[MD])\right\rangle \\&\quad = \frac{1}{N^2}\sum _{i,j} u_{ij} d_{ii} d_{jj} +\left\langle V_\mathrm {l},M({\mathcal {S}}{\mathcal {B}}^{-1}{\mathcal {Q}}[MD_\mathrm {o}])({\mathcal {B}}^{-1}{\mathcal {Q}}[MD_\mathrm {o}])\right\rangle , \end{aligned} \end{aligned}$$

with an appropriate deterministic matrix $u_{ij}$ having bounded entries. In particular, the cross terms vanish and the first term is bounded by3.19according to (3.14). By taking the off-diagonal part of (3.8a) and using the fact that *M* and $V_\mathrm {r}$ and therefore also $\mathcal {B}^{-1}\mathcal {Q}[M\mathcal {S}[V_\mathrm {r}]V_\mathrm {r}]$ are diagonal (cf. (b) above) we have

$$\begin{aligned} \left| {\mathcal {B}}^{-1}{\mathcal {Q}}[MD_\mathrm {o}]+G_\mathrm {o}\right| \prec \theta ^3+\theta \Bigl (\frac{\rho +\Xi }{N \eta }\Bigr )^{1/2}+\frac{\rho +\Xi }{N \eta }\lesssim N^{-\epsilon }\theta ^2+N^{\epsilon }\frac{\rho +\Xi }{N \eta } \end{aligned}$$

for any $\epsilon$ such that $\theta \lesssim N^{-\epsilon }$ by Young’s inequality in the last step. Together with (3.17), (3.14) and the assumption that $\left| G_\mathrm {o}\right| =\left| (G-M)_\mathrm {o}\right| \prec \Lambda$ we then compute

$$\begin{aligned} \begin{aligned}&\left\langle V_\mathrm {l},M({\mathcal {S}}{\mathcal {B}}^{-1}{\mathcal {Q}}[MD_\mathrm {o}])({\mathcal {B}}^{-1}{\mathcal {Q}}[MD_\mathrm {o}])\right\rangle \\&\quad = c \left\langle {{\,\mathrm{diag}\,}}({\mathbf {p}}{\mathbf {f}}) ({\mathcal {S}}{\mathcal {B}}^{-1}{\mathcal {Q}}[MD_\mathrm {o}])({\mathcal {B}}^{-1}{\mathcal {Q}}[MD_\mathrm {o}])\right\rangle + {\mathcal {O}}\,\left( \Bigl (\rho +\frac{\eta }{\rho }\Bigr )\frac{\rho +\Xi }{N\eta } \right) \\&\quad = c\left\langle {{\,\mathrm{diag}\,}}({\mathbf {p}}{\mathbf {f}}){\mathcal {S}}[G_\mathrm {o}]G_\mathrm {o}\right\rangle + {\mathcal {O}}\,\left( \Bigl (\rho +\frac{\eta }{\rho }\Bigr )\frac{\rho +\Xi }{N\eta } + \Bigl (\Bigl (\frac{\rho +\Xi }{N\eta }\Bigr )^{1/2}+\Lambda \Bigr )\Bigl [N^{-\epsilon }\theta ^2+N^{\epsilon }\frac{\rho +\Xi }{N \eta }\Bigr ]\right) . \end{aligned} \end{aligned}$$

Thus the bound on the second term on the lhs. in (3.15) follows together with (3.18)–(3.19) by ${\mathcal {S}}[G_\mathrm {o}] = T \odot G^t$ and (3.11d) via Lemma 3.6. This completes the proof of (3.15).$\square$

With (3.14) and (3.15) the upper bound (3.8c) on the error $\epsilon _*$ of the cubic equation (3.8b) takes the same form as the rhs. of (3.15) if *K* is sufficiently large depending on $\epsilon$. By the first estimate in (3.13b) we can redefine the control parameter $\Lambda$ on $\left| G-M\right|$ as $\Lambda :=\theta +((\rho +\Xi )/N \eta )^{1/2}$ and the claim (3.13a) follows directly with (3.15), thus completing the proof of Lemma 3.8. $\square$

### Bootstrapping

Now we will show that the difference $G-M$ converges to zero uniformly for all spectral parameters $z \in \mathbb {D}_\zeta$ as defined in (3.12). For convenience we refer to existing bounds on $G-M$ far away from the real line to establish a rough bound on $G-M$ in, say, $\mathbb {D}_1$. We then iteratively lower the threshold on $\eta$ by appealing to Proposition 3.4 and Lemma 3.8 until we establish the rough bound in all of $\mathbb {D}_\zeta$. As a second step we then improve the rough bound iteratively until we obtain Theorem 2.5.

#### Lemma 3.9

(Rough bound). For any $\zeta >0$ there exists a constant $c>0$ such that on the domain $\mathbb {D}_\zeta$ we have the rough bound

3.20 $$\begin{aligned} \left| G-M\right| \prec N^{-c}. \end{aligned}$$

#### Proof

The rough bound (3.20) in a neighbourhood of a cusp has first been established for Wigner-type random matrices in [8]. For the convenience of the reader we present a streamlined proof that is adapted to the current setting. The lemma is an immediate consequence of the following statement. Let $\zeta _\mathrm {s}>0$ be a sufficiently small *step size*, depending on $\zeta$. Then for any $\mathbb {N}_0\ni k\le 1/\zeta _\mathrm {s}$ on the domain $\mathbb {D}_{\max \{1-k\zeta _\mathrm {s}, \zeta \}}$ we have

3.21 $$\begin{aligned} \left| G-M\right| \prec N^{-4^{-k}\zeta }. \end{aligned}$$

We prove (3.21) by induction over *k*. For sufficiently small $\zeta$ the induction start $k=0$ holds due to the local law away from the self-consistent spectrum, e.g. [34, Theorem 2.1].

Now as induction hypothesis suppose that (3.21) holds on $\widetilde{\mathbb {D}}_{k} :=\mathbb {D}_{\max \{1-k\zeta _\mathrm {s}, \zeta \}}$, and in particular, $\left| G\right| \prec 1$, $\Vert G\Vert _p\le _{\epsilon ,p}N^{\epsilon }$ for any $\epsilon ,p$ according to Lemma 3.6. The monotonicity of the function $\eta \mapsto \eta \Vert G(\tau +\mathrm {i}\eta )\Vert _p$ (see e.g. [34, proof of Prop. 5.5]) implies $\Vert G\Vert _p\le _{\epsilon ,p} N^{\epsilon +\zeta _\mathrm {s}}\le N^{2 \zeta _\mathrm {s}}$ and therefore, according to Lemma 3.6, that $\left| G\right| \prec N^{2\zeta _\mathrm {s}}$ on $\widetilde{\mathbb {D}}_{k+1}$. This, in turn, implies $\left| D\right| \prec N^{-\zeta /3}$ on $\widetilde{\mathbb {D}}_{k+1}$ by (3.11a) and Lemma 3.6, provided $\zeta _\mathrm {s}$ is chosen small enough. We now fix $\mathbf {x},\mathbf {y}$ and a large integer *K* as the parameters of $\Vert \cdot \Vert _*=\Vert \cdot \Vert _*^{\mathbf {x},\mathbf {y},K}$ for the rest of the proof and omit them from the notation but we stress that all estimates will be uniform in $\mathbf {x},\mathbf {y}$. We find $\sup _{z \in {\widetilde{\mathbb {D}}}_{k+1}}\Vert D(z)\Vert _*\prec N^{-\zeta /3}$, by using a simple union bound and $\Vert \partial _z D\Vert \le N^C$ for some $C>0$. Thus, for *K* large enough, we can use (3.8a), (3.8b), (3.8c) and (3.9) to infer

3.22 $$\begin{aligned} \begin{aligned} \left| \Theta ^3+ \xi _2 \Theta ^2 +\xi _1 \Theta \right|&\lesssim N^{1/2K}\Vert D\Vert _*\prec N^{1/2K-\zeta /3},\\ \Vert G-M\Vert _*&\lesssim \left| \Theta \right| + N^{1/K}\Vert D\Vert _*\prec \left| \Theta \right| +N^{1/K-\zeta /3}, \end{aligned} \end{aligned}$$

on the event $\Vert G-M\Vert _*+\Vert D\Vert _*\lesssim N^{-10/K}$, and on $\widetilde{\mathbb {D}}_{k+1}$. Now we use the following lemma [10, Lemma 10.3] to translate the first estimate in (3.22) into a bound on $\left| \Theta \right|$. For the rest of the proof we keep $\tau =\mathfrak {R}z$ fixed and consider the coefficients $\xi _1,\xi _2$ and $\Theta$ as functions of $\eta$.

#### Lemma 3.10

(Bootstrapping cubic inequality). For $0<\eta _*<\eta ^*<\infty$ let $\xi _1,\xi _2:[\eta _*,\eta ^*] \rightarrow \mathbb {C}$ be complex valued functions and $\widetilde{\xi }_1,\widetilde{\xi }_2, d:[\eta _*,\eta ^*] \rightarrow \mathbb {R}^+$ be continuous functions such that at least one of the following holds true:

(i) $\left| {\xi }_1\right| \sim \widetilde{\xi }_1$, $\left| {\xi }_2\right| \sim \widetilde{\xi }_2$, and $\widetilde{\xi }_2^3/d,\widetilde{\xi }_1^3/d^2,\widetilde{\xi }_1^2/d\widetilde{\xi }_2$ are monotonically increasing, and $d^2/\widetilde{\xi }_1^3+d\widetilde{\xi }_2/\widetilde{\xi }_1^2\ll 1$ at $\eta ^*$,
(ii) $\left| {\xi }_1\right| \sim \widetilde{\xi }_1$, $\left| {\xi }_2\right| \lesssim \widetilde{\xi }_1^{1/2}$, and $\widetilde{\xi }_1^3/d^2$ is monotonically increasing.

Then any continuous function $\Theta :[\eta _*,\eta ^*] \rightarrow \mathbb {C}$ that satisfies the cubic inequality  on $[\eta _*,\eta ^*]$, has the property

3.23 $$\begin{aligned} \text {If} \quad \left| \Theta \right| \lesssim \min \bigg \{d^{1/3}, \frac{d^{1/2}}{{\widetilde{\xi }_2^{1/2}}},\frac{d}{\widetilde{\xi }_1}\bigg \} \text { at } \eta ^*, \quad \text {then} \quad \left| \Theta \right| \lesssim \min \bigg \{d^{1/3}, \frac{d^{1/2}}{{\widetilde{\xi }_2^{1/2}}},\frac{d}{\widetilde{\xi }_1}\bigg \} \text { on } [\eta _*,\eta ^*].\nonumber \\ \end{aligned}$$

With direct arithmetics we can now verify that the coefficients $\xi _1,\xi _2$ in (3.8b) and the auxiliary coefficients $\widetilde{\xi }_1,\widetilde{\xi }_2$ defined in (3.7e) satisfy the assumptions in Lemma 3.10 with the choice of the constant function $d=N^{-4^{-k}\zeta +\delta }$ for any $\delta >0$, by using only the information on $\xi _1,\xi _2$ given by the comparison relations (3.8d). As an example, in the regime where $\tau _0$ is a right edge and $\omega \sim \Delta$, we have $\widetilde{\xi }_1 \sim (\eta +\Delta )^{2/3}$ and $\widetilde{\xi }_2 \sim (\eta +\Delta )^{1/3}$ and both functions are monotonically increasing in $\eta$. Then Assumption (ii) of Lemma 3.10 is satisfied. All other regimes are handled similarly.

We now set $\eta ^*:=N^{-k\zeta _\mathrm {s}}$ and

$$\begin{aligned} \eta _*:=\inf \left\{ \eta \in [N^{-(k+1) \zeta _\mathrm {s}},\eta ^*]\Bigg |\sup _{\eta '\ge \eta }\Vert G(\tau +\mathrm {i}\eta ')-M(\tau +\mathrm {i}\eta ')\Vert _*\le N^{-10/K}/2\right\} . \end{aligned}$$

By the induction hypothesis we have $\left| \Theta (\eta ^*)\right| \lesssim d \lesssim \min \{ d^{1/3}, d^{1/2}\widetilde{\xi }_2^{-1/2},d \widetilde{\xi }_1^{-1}\}$ with overwhelming probability, so that the condition in (3.23) holds, and conclude $\left| \Theta (\eta )\right| \prec d^{1/3}=N^{-(4^{-k}\zeta -\delta )/3}$ for $\eta \in [\eta _*,\eta ^*]$. For small enough $\delta >0$ the second bound in (3.22) implies $\Vert G-M\Vert _*\prec N^{-4^{k+1}\zeta }$. By continuity and the definition of $\eta _*$ we conclude $\eta _*=N^{-(k+1) \zeta _\mathrm {s}}$, finishing the proof of (3.21). $\square$

#### Proof of Theorem 2.5

The bounds within the proof hold true uniformly for $z\in \mathbb {D}_\zeta$, unless explicitly specified otherwise. We therefore suppress this qualifier in the following statements. First we apply Lemma 3.8 with the choice $\Xi =\Lambda$, i.e. we do not treat the imaginary part of the resolvent separately. With this choice the first inequality in (3.13b) becomes self-improving and after iteration shows that

3.24 $$\begin{aligned} \left| G-M\right| \prec \theta + \sqrt{\frac{\rho }{N \eta }}+ \frac{1}{N \eta }, \end{aligned}$$

and, in other words, (3.13a) holds with $\Xi =\theta + (\rho /N\eta )^{1/2}+ 1/N\eta$. This implies that if $\left| \Theta \right| \prec \theta \lesssim N^{-c}$ for some arbitrarily small $c>0$, then

3.25 $$\begin{aligned} \left| \Theta ^3+ \xi _2 \Theta ^2 +\xi _1 \Theta \right| \lesssim N^{{5}\widetilde{\epsilon }}d_*+N^{-\widetilde{\epsilon }}(\theta ^3 + \widetilde{\xi }_2\theta ^2) \end{aligned}$$

holds for all sufficiently small $\widetilde{\epsilon }$ with overwhelming probability, where we defined

3.26 $$\begin{aligned} d_*:=\widetilde{\xi }_2 \bigg (\frac{\widetilde{\rho }}{N \eta } +\frac{1}{(N \eta )^2}\bigg ) +\frac{1}{(N \eta )^3} + \bigg ( \frac{\widetilde{\rho }}{N \eta }\bigg )^{3/2}. \end{aligned}$$

For this conclusion we used the comparison relations (3.8d), Proposition 3.2(iv) as well as (3.7b), and the bound $\sqrt{\eta /\rho }\sim \sqrt{\eta /\widetilde{\rho }}\lesssim \widetilde{\xi }_2$. $\square$

The bound (3.25) is a self-improving estimate on $\left| \Theta \right|$ in the following sense. For $k \in \mathbb {N}$ and $l \in \mathbb {N}\cup \{*\}$ let

$$\begin{aligned} d_k :=\max \{N^{-k\widetilde{\epsilon }},N^{6\widetilde{\epsilon }}d_*\}, \qquad \theta _l:=\min \bigg \{d_l^{1/3}, \frac{d_l^{1/2}}{\widetilde{\xi }_2^{1/2}},\frac{d_l}{\widetilde{\xi }_1}\bigg \}. \end{aligned}$$

Then (3.25) with $\left| \Theta \right| \prec \theta _k$ implies that $\left| \Theta ^3+ \xi _2 \Theta ^2 +\xi _1 \Theta \right| \lesssim N^{-\widetilde{\epsilon }} d_k$. Applying Lemma 3.10 with $d=N^{-\widetilde{\epsilon }}{d_k}$, $\eta ^*\sim 1$, $\eta _*=N^{\zeta -1}$ yields the improvement $\left| \Theta \right| \prec \theta _{k+1}$. Here we needed to check the condition in (3.23) but at $\eta ^*\sim 1$ we have $\widetilde{\xi }_1\sim 1$, so $\left| \Theta \right| \lesssim N^{-\widetilde{\epsilon }}d_k\le d_{k+1}\sim \theta _{k+1}$. After a *k*-step iteration until $N^{-k\widetilde{\epsilon }}$ becomes smaller than $N^{6\widetilde{\epsilon }}d_*$, we find $\left| \Theta \right| \prec \theta _*$, where we used that $\widetilde{\epsilon }$ can be chosen arbitrarily small. We are now ready to prove the following bound which we, for convenience, record as a proposition.

#### Proposition 3.11

For any $\zeta >0$ we have the bounds

3.27 $$\begin{aligned} \left| G-M\right| \prec \theta _*+ \sqrt{\frac{\rho }{N \eta }}+ \frac{1}{N \eta },\qquad \left| G-M\right| _{\mathrm {av}}\prec \theta _*+\frac{\rho }{N \eta }+\frac{1}{(N \eta )^2} \quad \text {in}\quad \mathbb {D}_\zeta ,\nonumber \\ \end{aligned}$$

where $\theta _*:=\min \{d_*^{1/3},d_*^{1/2}/\widetilde{\xi }_2^{1/2},d_*/\widetilde{\xi }_1\}$, and $d_*,\widetilde{\rho },\widetilde{\xi }_1,\widetilde{\xi }_2$ are given in (3.26), (3.7b) and (3.7e), respectively.

#### Proof

Using $\left| \Theta \right| \prec \theta _*$ proven above, we apply (3.24) with $\theta =\theta _*$ to conclude the first inequality in (3.27). For the second inequality in (3.27) we use the estimate on $\left| G-M\right| _{\mathrm {av}}$ from (3.13b) with $\theta = \theta _*$ and $\Xi = (\rho /N\eta )^{1/2}+ 1/N\eta$. $\square$

The bound on $\left| G-M\right|$ from (3.27) implies a complete delocalisation of eigenvectors uniformly at singularities of the scDOS. The following corollary was established already in [8, Corollary 1.14] and, given (3.27), the proof follows the same line of reasoning.

#### Corollary 3.12

(Eigenvector delocalisation). Let ${\mathbf {u}}\in \mathbb {C}^N$ be an eigenvector of *H* corresponding to an eigenvalue $\lambda \in \tau _0+(-c,c)$ for some sufficiently small positive constant $c \sim 1$. Then for any deterministic $\mathbf {x}\in \mathbb {C}^N$ we have

$$\begin{aligned} \left| \left\langle {\mathbf {u}}, \mathbf {x}\right\rangle \right| \prec \frac{1}{\sqrt{N}} \Vert {\mathbf {u}}\Vert \Vert \mathbf {x}\Vert . \end{aligned}$$

The bounds (3.27) simplify in the regime $\eta \ge N^\zeta \eta _\mathrm {f}$ above the typical eigenvalue spacing to

3.28 $$\begin{aligned} \left| G-M\right| \prec \sqrt{\frac{\rho }{N \eta }}+ \frac{1}{N \eta },\qquad \left| G-M\right| _{\mathrm {av}}\prec \frac{1}{N \eta }, \qquad \text {for} \quad \eta \ge N^\zeta \eta _\mathrm {f} \end{aligned}$$

using Lemma 3.3 which implies $\theta _*\le d_*/\widetilde{\xi }_1\le 1/N\eta$. The bound on $\left| G-M\right| _{\mathrm {av}}$ is further improved in the case when $\tau _0=\mathfrak {e}_-$ is an edge and, in addition to $\eta \ge N^\zeta \eta _\mathrm {f}$, we assume $N^{\delta }\eta \le \omega \le \Delta /2$ for some $\delta >0$, i.e. if $\omega$ is well inside a gap of size $\Delta \ge N^{\delta +\zeta }\eta _\mathrm {f}$. Then we find $\Delta >N^{-3/4}$ by the definition of $\eta _\mathrm {f}=\Delta ^{1/9}/N^{2/3}$ in (2.7) and use Lemma 3.3 and (3.7b), (3.7e) to conclude

3.29 $$\begin{aligned} \theta _*+\frac{\widetilde{\rho }}{N \eta }+\frac{1}{(N \eta )^2}\lesssim \frac{\widetilde{\xi }_2}{\widetilde{\xi }_1} \bigg (\frac{\widetilde{\rho }}{N \eta }+ \frac{1}{(N \eta )^2}\bigg )\sim \frac{\Delta ^{1/6}}{\omega ^{1/2}}\bigg (\frac{\eta }{\Delta ^{1/6}\omega ^{1/2}}+\frac{1}{N \eta }\bigg )\frac{1}{N \eta }\lesssim \frac{N^{-\delta /2}}{N \eta }.\nonumber \\ \end{aligned}$$

In the last bound we used $1/N\omega \le N^{-\delta }/N\eta$ and $\Delta ^{1/6}/(N\eta \omega ^{1/2})\le N^{-\delta /2}$. Using (3.29) in (3.27) yields the improvement

3.30 $$\begin{aligned} \left| G-M\right| _{\mathrm {av}}\prec \frac{N^{-\delta /2}}{N \eta }, \qquad \text {for} \quad \tau =\mathfrak {e}_-+\omega ,\quad \Delta /2\ge \omega \ge N^{\delta } \eta \ge N^{\zeta +\delta }\eta _\mathrm {f}.\qquad \end{aligned}$$

The bounds on $\left| G-M\right| _{\mathrm {av}}$ from (3.28) and (3.30), inside and outside the self-consistent spectrum, allow us to show the uniform rigidity, Corollary 2.6. We postpone these arguments until after we finish the proof of Theorem 2.5. The uniform rigidity implies that for ${{\,\mathrm{dist}\,}}(z, {{\,\mathrm{supp}\,}}\rho ) \ge N^{\zeta }\eta _\mathrm {f}$ we can estimate the imaginary part of the resolvent via

3.31 $$\begin{aligned} \mathfrak {I}\left\langle \mathbf {x}, G\mathbf {x}\right\rangle = \sum _{\lambda }\frac{\eta \left| \left\langle {\mathbf {u}}_\lambda , \mathbf {x}\right\rangle \right| ^2}{\eta ^2 + (\tau _0+\omega -\lambda )^2} \prec \eta + \frac{1}{N}\sum _{\left| \lambda -\tau _0\right| \le c}\frac{\eta }{\eta ^2 + (\tau _0+\omega -\lambda )^2}\prec \rho (z),\nonumber \\ \end{aligned}$$

for any normalised $\mathbf {x}\in \mathbb {C}^{N}$, where ${\mathbf {u}}_\lambda$ denotes the normalised eigenvector corresponding to $\lambda$. For the first inequality in (3.31) we used Corollary 3.12 and for the second we applied Corollary 2.6 that allows us to replace the Riemann sum with an integral as $[\eta ^2+(\tau _0+\omega -\lambda )^2]^{1/2}=\left| z-\lambda \right| \ge N^\zeta \eta _\mathrm {f}$.

Using with (3.31), we apply Lemma 3.8, repeating the strategy from the beginning of the proof. But this time we can choose the control parameter $\Xi =\rho$. In this way we find

3.32 $$\begin{aligned} \left| G-M\right| \prec \theta _{\#} + \sqrt{\frac{\rho }{N \eta }},\quad \left| G-M\right| _{\mathrm {av}}\prec \theta _{\#}+\frac{\rho }{N \eta }, \quad \text {for} \quad {{\,\mathrm{dist}\,}}(z, {{\,\mathrm{supp}\,}}\rho ) \ge N^{\zeta }\eta _\mathrm {f},\nonumber \\ \end{aligned}$$

where we defined

$$\begin{aligned} \theta _{\#} :=\min \bigg \{\frac{d_\#}{\widetilde{\xi }_1}, \frac{d_\#^{1/2}}{\widetilde{\xi }_2^{1/2}}, d_\#^{1/3}\bigg \}, \qquad d_{\#}:=\widetilde{\xi }_2\frac{\widetilde{\rho } }{N \eta } + \bigg ( \frac{\widetilde{\rho }}{N \eta }\bigg )^{3/2}. \end{aligned}$$

Note that the estimates in (3.32) are simpler than those in (3.27). The reason is that the additional terms $1/N\eta$, $1/(N\eta )^2$ and $1/(N\eta )^3$ in (3.27) are a consequence of the presence of $\Xi$ in (3.13a), (3.13b). With $\Xi =\rho$ these are immediately absorbed into $\rho$ and not present any more. The second term in the definition of $d_\#$ can be dropped since we still have $\widetilde{\xi }_2\gtrsim (\rho /N\eta )^{1/2}$ (this follows from Lemma 3.3 if $\eta \ge N^\zeta \eta _\mathrm {f}$, and directly from (3.7b), (3.7e) if $\omega \ge N^\zeta \eta _\mathrm {f}$). This implies $\theta _\#\lesssim d_\#^{1/2}/\widetilde{\xi }_2^{1/2}\lesssim (\rho /N\eta )^{1/2}$, so the first bound in (3.32) proves (2.8a).

Now we turn to the proof of (2.8b). Given the second bound in (3.28), it is sufficient to consider the case when $\tau =\mathfrak {e}_-+\omega$ and $\eta \le \omega \le \Delta /2$ with $\omega \ge N^\zeta \eta _\mathrm {f}$. In this case Proposition 3.2 yields $\widetilde{\xi }_2\widetilde{\rho }/\widetilde{\xi }_1+\widetilde{\rho }\lesssim \eta /\omega \sim \eta /{{\,\mathrm{dist}\,}}(z,{{\,\mathrm{supp}\,}}\rho )$. Thus we have

$$\begin{aligned} \theta _\#+\frac{\rho }{N\eta }\lesssim \frac{d_\#}{\widetilde{\xi }_1}+\frac{\widetilde{\rho }}{N\eta }\lesssim \frac{1}{N{{\,\mathrm{dist}\,}}(z,{{\,\mathrm{supp}\,}}\rho )} \end{aligned}$$

and therefore the second bound in (3.32) implies (2.8b). This completes the proof of Theorem 2.5. $\square$

### Rigidity and absence of eigenvalues

The proofs of Corollaries 2.6 and 2.8 rely on the bounds on $\left| G-M\right| _{\mathrm {av}}$ from (3.28) and (3.30). As before, we may restrict ourselves to the neighbourhood of a local minimum $\tau _0 \in {{\,\mathrm{supp}\,}}\rho$ of the scDOS which is either an internal minimum with a small value of $\rho (\tau _0)>0$, a cusp location or a right edge adjacent to a small gap of length $\Delta >0$. All other cases, namely the bulk regime and regular edges adjacent to large gaps, have been treated prior to this work [8, 11].

#### Proof of Corollary 2.8

Let us denote the empirical eigenvalue distribution of *H* by $\rho _H = \frac{1}{N} \sum _{i=1}^N \delta _{\lambda _i}$ and consider the case when $\tau _0=\mathfrak {e}_-$ is a right edge, $\Delta \ge N^{\delta } \eta _\mathrm {f}$ for any $\delta >0$ and $\eta _\mathrm {f}=\eta _\mathrm {f}(\mathfrak {e}_-)\sim \Delta ^{1/9}N^{-2/3}$. Then we show that there are no eigenvalues in $\mathfrak {e}_-+[N^{\delta }\eta _\mathrm {f}, \Delta /2]$ with overwhelming probability. We apply [8, Lemma 5.1] with the choices

$$\begin{aligned} \nu _1:=\rho ,\quad \nu _2:=\rho _H, \quad \eta _1:=\eta _2:=\epsilon :=N^{\zeta }\eta _\mathrm {f},\quad \tau _1:=\mathfrak {e}_-+\omega ,\quad \tau _2 :=\mathfrak {e}_-+\omega +N^{\zeta }\eta _\mathrm {f}, \end{aligned}$$

for any $\omega \in [N^{\delta }\eta _\mathrm {f}, \Delta /2]$ and some $\zeta \in (0,\delta /4)$. We use (3.30) to estimate the error terms $J_1, J_2$ and $J_3$ from [8, Eq. (5.2)] by $N^{2\zeta -\delta /2-1}$ and see that $(\rho _H-\rho )([\tau _1,\tau _2]) =\rho _H([\tau _1,\tau _2]) \prec N^{2\zeta -\delta /2-1}$, showing that with overwhelming probability the interval $[\tau _1,\tau _2]$ does not contain any eigenvalues. A simple union bound finishes the proof of Corollary 2.8. $\square$

#### Proof of Corollary 2.6

Now we establish Corollary 2.6 around a local minimum $\tau _0 \in {{\,\mathrm{supp}\,}}\rho$ of the scDOS. Its proof has two ingredients. First we follow the strategy of the proof of [8, Corollary 1.10] to see that

3.33 $$\begin{aligned} \left| (\rho -\rho _H)((-\infty ,\tau _0+\omega ]) \right| \prec \frac{1}{N}, \end{aligned}$$

for any $\left| \omega \right| \le c$, i.e. we have a very precise control on $\rho _H$. In contrast to the statement in that corollary we have a local law (3.28) with uniform $1/N\eta$ error and thus the bound (3.33) does not deteriorate close to $\tau _0$. We warn the reader that the standard argument inside the proof of [8, Corollary 1.10] has to be adjusted slightly to arrive at (3.33). In fact, when inside that proof the auxiliary result [8, Lemma 5.1] is used with the choice $\tau _1=-10$, $\tau _2 =\tau$, $\eta _1=\eta _2=N^{\zeta -1}$ for some $\zeta >0$, this choice should be changed to $\tau _1=-C$, $\tau _2 =\tau$, $\eta _1=N^{\zeta -1}$ and $\eta _2=N^{\zeta }\eta _{\mathrm {f}}(\tau )$, where $C>0$ is chosen sufficiently large such that $\tau _1$ lies far to the left of the self-consistent spectrum.

The control (3.33) suffices to prove Corollary 2.6 for all $\tau =\tau _0 +\omega$ except for the case when $\tau _0=\mathfrak {e}_-$ is an edge at a gap of length $\Delta \ge N^\zeta \eta _\mathrm {f}$ and $\omega \in [- N^\zeta \eta _\mathrm {f},0]$ for some fixed $\zeta >0$ and $\eta _\mathrm {f} = \eta _\mathrm {f}(\mathfrak {e}_-) \sim \Delta ^{1/9}/N^{2/3}$, i.e. except for some $N^\zeta$ eigenvalues close to the edge with arbitrarily small $\zeta >0$. In all other cases, the proof follows the same argument as the proof of [8, Corollary 1.11] using the uniform 1/*N*-bound from (3.33) and we omit the details here.

The reason for having to treat the eigenvalues very close to the edge $\mathfrak {e}_-$ separately is that (3.33) does not give information on which side of the gap these $N^\zeta$ eigenvalues are found. To get this information requires the second ingredient, the *band rigidity*,

3.34 $$\begin{aligned} \mathbf {P}\big [ \rho ((-\infty , \mathfrak {e}_-+\omega ])= \rho _H((-\infty , \mathfrak {e}_-+\omega ]) \big ]\ge 1-N^{-\nu }, \end{aligned}$$

for any $\nu \in \mathbb {N}$, $\Delta \ge \omega \ge N^\zeta \eta _\mathrm {f}$ and large enough *N*. The combination of (3.34) and (3.33) finishes the proof of Corollary 2.6.

Band rigidity has been shown in case $\Delta$ is bounded from below in [11] as part of the proof of Corollary 2.5. We will now adapt this proof to the case of small gap sizes $\Delta \ge N^{\zeta -3/4}$. Since by Corollary 2.8 with overwhelming probability there are no eigenvalues in $\mathfrak {e}_-+[N^\zeta \eta _\mathrm {f},\Delta /2]$, it suffices to show (3.34) for $\omega = \Delta /2$. As in the proof of [11, Corollary 2.5] we consider the interpolation

$$\begin{aligned} H_t:=\sqrt{1-t}W+A-t{\mathcal {S}}M(\tau ),\qquad t \in [0,1], \end{aligned}$$

between the original random matrix $H=H_0$ and the deterministic matrix $H_1=A-{\mathcal {S}}M(\tau )$, for $\tau =\mathfrak {e}_- +\Delta /2$. The interpolation is designed such that the solution $M_t$ of the MDE corresponding to $H_t$ is constant at spectral parameter $\tau$, i.e. $M_t(\tau )=M(\tau )$. Let $\rho _t$ denote the scDOS of $H_t$. Exactly as in the proof from [11] it suffices to show that no eigenvalue crosses the gap along the interpolation with overwhelming probability, i.e. that for any $\nu \in \mathbb {N}$ we have

3.35 $$\begin{aligned} \mathbf {P}\big [ {\mathfrak {a}}_t \in {{\,\mathrm{Spec}\,}}(H_t) \text { for some } t \in [0,1]\big ] \le \frac{C(\nu )}{N^{\nu }}. \end{aligned}$$

Here $t \rightarrow {\mathfrak {a}}_t \in \mathbb {R}{\setminus } {{\,\mathrm{supp}\,}}\rho _t$ is some spectral parameter inside the gap, continuously depending on *t*, such that ${\mathfrak {a}}_0 =\tau$. In [11] ${\mathfrak {a}}_t$ was chosen independent of *t*, but the argument remains valid with any other choice of ${\mathfrak {a}}_t$. We call $I_t$ the connected component of $\mathbb {R}{\setminus } {{\,\mathrm{supp}\,}}\rho _t$ that contains ${\mathfrak {a}}_t$ and denote $\Delta _t = \left| I_t\right|$ the gap length. In particular, $\Delta _0=\Delta$ and $\tau \in I_t$ for all $t \in [0,1]$ by [10, Lemma 8.1(ii)]. For concreteness we choose ${\mathfrak {a}}_t$ to be the spectral parameter lying exactly in the middle of $I_t$. The 1/3-Hölder continuity of $\rho _t$, hence $I_t$ and ${\mathfrak {a}}_t$ in *t* follows from [10, Proposition 10.1(a)]. Via a simple union bound it suffices to show that for any fixed $t \in [0,1]$ we have no eigenvalue in ${\mathfrak {a}}_t+[-N^{-100},N^{-100}]$.

Since $\Vert W\Vert \lesssim 1$ with overwhelming probability, in the regime $t \ge 1-\epsilon$ for some small constant $\epsilon >0$, the matrix $H_t$ is a small perturbation of the deterministic matrix $H_1$ whose resolvent $(H_1-\tau )^{-1} =M(\tau )$ at spectral parameter $\tau$ is bounded by Assumption (C), in particular $\Delta _1\gtrsim 1$. By 1/3-Hölder continuity hence $\Delta _t\gtrsim 1$, and ${{\,\mathrm{Spec}\,}}(H_t) \subset {{\,\mathrm{Spec}\,}}(H_1)+[-C\epsilon ^{1/3},C\epsilon ^{1/3}]$ for some $C\sim 1$ in this regime with very high probability. Since ${{\,\mathrm{Spec}\,}}(H_1) \subset {{\,\mathrm{supp}\,}}\rho _t+[-C\epsilon ^{1/3},C\epsilon ^{1/3}]$ by [10, Proposition 10.1(a)] there are no eigenvalues of $H_t$ in a neighbourhood of ${\mathfrak {a}}_t$, proving (3.35) for $t\ge 1-\epsilon$.

For $t \in [\epsilon , 1-\epsilon ]$ we will now show that $\Delta _t \sim _\epsilon 1$ for any $\epsilon >0$. In fact, we have ${{\,\mathrm{dist}\,}}(\tau , {{\,\mathrm{supp}\,}}\rho _t)~\gtrsim _\epsilon ~1$. This is a consequence of [10, Lemma D.1]. More precisely, we use the equivalence of (iii) and (v) of that lemma. We check (iii) and conclude the uniform distance to the self-consistent spectrum by (v). Since $M_t(\tau )=M(\tau )$ and $\Vert M(\tau )\Vert \lesssim 1$ we only need to check that the stability operator ${\mathcal {B}}_t = t+(1-t){\mathcal {B}}$ of $H_t$ has a bounded inverse. We write ${\mathcal {B}}_t = {\mathcal {C}}(1-(1-t) \widetilde{{\mathcal {C}}}{\mathcal {F}}){\mathcal {C}}^{-1}$ in terms of the saturated self-energy operator ${\mathcal {F}} = {\mathcal {C}}{\mathcal {S}}{\mathcal {C}}$, where ${\mathcal {C}}[R]:=\left| M(\tau )\right| ^{1/2}R\left| M(\tau )\right| ^{1/2}$ and $\widetilde{{\mathcal {C}}}[R]:=({{\,\mathrm{sgn}\,}}M(\tau ))R({{\,\mathrm{sgn}\,}}M(\tau ))$. Afterwards we use that $\Vert {\mathcal {F}}\Vert _{\mathrm {hs} \rightarrow \mathrm {hs}}\le 1$ (cf. [7, Eq. (4.24)]) and $\Vert \widetilde{{\mathcal {C}}}\Vert _{\mathrm {hs}\rightarrow \mathrm {hs}}=1$ to first show the uniform bound $\Vert {\mathcal {B}}_t\Vert _{\mathrm {hs} \rightarrow \mathrm {hs}} \lesssim 1/t$ and then improve the bound to $\Vert {\mathcal {B}}_t\Vert \lesssim 1/t$ using the trick of expanding in a geometric series from [7, Eqs. (4.60)–(4.63)]. This completes the argument that $\Delta _t\sim _\epsilon 1$. Now we apply [34, Corollary 2.3] to see that there are no eigenvalues of $H_t$ around ${\mathfrak {a}}_t$ as long as *t* is bounded away from zero and one, proving (3.35) for this regime.

Finally, we are left with the regime $t \in [0,\epsilon ]$ for some sufficiently small $\epsilon >0$. By [10, Proposition 10.1(a)] the self-consistent Green’s function $M_t$ corresponding to $H_t$ is bounded even in a neighbourhood of $\tau$, whose size only depends on model parameters. In particular, Assumptions (A)–(C) are satisfied for $H_t$ and Corollary 2.8, which was already proved above, is applicable. Thus it suffices to show that the size $\Delta _t$ of the gap in ${{\,\mathrm{supp}\,}}\rho _t$ containing $\tau$ is bounded from below by $\Delta _t \ge N^{\zeta -3/4}$ for some $\zeta >0$. The size of the gap can be read off from the following relationship between the norm of the saturated self-energy operator and the size of the gap: Let *H* be a random matrix satisfying (A)–(C) and $\tau$ be well inside the interior of the gap of length $\Delta \in [0,c]$ in the self-consistent spectrum for a sufficiently small $c\sim 1$. Then

3.36 $$\begin{aligned}&1-\Vert {\mathcal {F}}(\tau )\Vert _{\mathrm {hs} \rightarrow \mathrm {hs}} \sim \lim _{\eta \searrow 0}\frac{\eta }{\rho (\tau +\mathrm {i}\eta )} \sim (\Delta +{{\,\mathrm{dist}\,}}(\tau ,{{\,\mathrm{supp}\,}}\rho ))^{1/6}\nonumber \\&\quad {{\,\mathrm{dist}\,}}(\tau ,{{\,\mathrm{supp}\,}}\rho )^{1/2} \sim \Delta ^{2/3}, \end{aligned}$$

where in the first step we used [7, Eqs. (4.23)–(4.25)], in the second step (3.7b), and in the last step that ${{\,\mathrm{dist}\,}}(\tau ,{{\,\mathrm{supp}\,}}\rho )\sim \Delta$. Applying the analogue of (3.36) for $H_t$ with ${\mathcal {F}}_t(\tau )$ and using that ${{\,\mathrm{dist}\,}}(\tau ,\rho _t)\lesssim \Delta _t$, we obtain $1-\Vert {\mathcal {F}}_t(\tau )\Vert _{\mathrm {hs}\rightarrow \mathrm {hs}}\lesssim \Delta _t^{2/3}$. Combining this inequality with (3.36) and using that ${\mathcal {F}}_t(\tau )=(1-t)F(\tau )$ for $t\in [0,c]$, we have $\Delta _t^{3/2} \gtrsim t +(1-t)\Delta ^{2/3}$, i.e. $\Delta _t\gtrsim t^{3/2}+\Delta$. In particular, the gap size $\Delta _t$ never drops below $c\Delta \gtrsim N^{\zeta -3/4}$. This completes the proof of the last regime in (3.35). $\square$

## Cusp Fluctuation Averaging and Proof of Theorem 3.7

We will use the graphical multivariate cumulant expansion from [34] which automatically exploits the self-energy renormalization of $D$ to highest order. Since the final formal statement requires some custom notations, we first give a simple motivating example to illustrate the type of expansion and its graphical representation. If $W$ is Gaussian, then integration by parts shows that

4.1 $$\begin{aligned} \begin{aligned} {{\,\mathrm{\mathbf {E}}\,}}\left\langle D\right\rangle ^2 =&\sum _{\alpha ,\beta }\kappa (\alpha ,\beta ){{\,\mathrm{\mathbf {E}}\,}}\left\langle \Delta ^{\alpha } G\right\rangle \left\langle \Delta ^{\beta } G\right\rangle \\&+ \sum _{\alpha _1,\beta _1} \kappa (\alpha _1,\beta _1)\sum _{\alpha _2,\beta _2} \kappa (\alpha _2,\beta _2) {{\,\mathrm{\mathbf {E}}\,}}\left\langle \Delta ^{\alpha _1} G\Delta ^{\beta _2} G\right\rangle \left\langle \Delta ^{\alpha _2} G\Delta ^{\beta _1}G\right\rangle , \end{aligned} \end{aligned}$$

where we recall that $\kappa (\alpha , \beta ):=\kappa (w_\alpha ,w_\beta )$ is the second cumulant of the matrix entries $w_\alpha ,w_\beta$ index by double indices $\alpha =(a,b)$, $\beta =(a',b')$, and $\Delta ^{(a,b)}$ denotes the matrix of all zeros except for an 1 in the (*a*, *b*)th entry. Since for non-Gaussian $W$ or higher powers of $\left\langle D\right\rangle$ the expansion analogous to (4.1) consists of much more complicated polynomials in resolvent entries, we represent them concisely as the *values* of certain *graphs*. As an example, the rhs. of (4.1) is represented simply by4.2The graphs retain only the relevant information of the complicated expansion terms and chains of estimates can be transcribed into simple graph surgeries. Graphs also help identify critical terms that have to be estimated more precisely in order to obtain the improved high moment bound on $D$. For example, the key cancellation mechanism behind the cusp fluctuation averaging is encoded in a small distinguished part of the expansion that can conveniently be identified as certain subgraphs, called the $\sigma$-*cells*, see Definition 4.10 later. It is easy to count, estimate and manipulate $\sigma$-cells as part of a large graph, while following the same operations on the level of formulas would be almost intractable.

First we review some of the basic nomenclature from [34]. We consider random matrices $H=A+W$ with diagonal expectation *A* and complex Hermitian or real symmetric zero mean random component *W* indexed by some abstract set *J* of size $\left| J\right| =N$. We recall that Greek letters $\alpha ,\beta ,\ldots$ stand for labels, i.e. double-indices from $I=J\times J$, whereas Roman letters $a,b,\ldots$ stand for single indices. If $\alpha =(a,b)$, then we set $\alpha ^t:=(b,a)$ for its transpose. Underlined Greek letters stand for multisets of labels, whereas bold-faced Greek letters stand for tuples of labels with the counting combinatorics being their—for our purposes—only relevant difference.

According to [34, Proposition 4.4] with $\mathcal {N}(\alpha )=\{\alpha ,\alpha ^t\}$ it follows from the assumed independence that for general (conjugate) linear functionals $\Lambda ^{(k)}$, of bounded norm $\Vert \Lambda ^{(k)}\Vert ={\mathcal {O}}\,\left( 1\right)$

4.3a $$\begin{aligned} {{\,\mathrm{\mathbf {E}}\,}}\prod _{k\in [p]}\Lambda ^{(k)}(D) = {{\,\mathrm{\mathbf {E}}\,}}\prod _{l\in [p]}\bigg (1+\sum _{\alpha _l,{\varvec{\beta }}_l}^{\sim (l)}\bigg )\prod _{k\in [p]} {\left\{ \begin{array}{ll} \Lambda ^{(k)}_{\alpha _k,{\underline{\beta }}^k}&{}\text {if } \sum _{\alpha _k}\\ \Lambda ^{(k)}_{{\underline{\beta }}^k_{<k},{\underline{\beta }}^k_{>k}}&{}\text {else} \end{array}\right. } + {\mathcal {O}}\,\left( N^{-p}\right) ,\nonumber \\ \end{aligned}$$

where we recall that4.3band that

4.3c $$\begin{aligned} \begin{aligned} \Lambda _{\alpha _1,\ldots ,\alpha _k}&:=-(-1)^k\Lambda (\Delta ^{\alpha _{1}} G\ldots \Delta ^{\alpha _{k}} G),\quad \Lambda _{\{\alpha _1,\ldots ,\alpha _m\}}:=\sum _{\sigma \in S_m}\Lambda _{\alpha _{\sigma (1)},\ldots ,\alpha _{\sigma (m)}}, \\ \Lambda _{\alpha ,\{\alpha _1,\ldots ,\alpha _m\}}&:=\sum _{\sigma \in S_m}\Lambda _{\alpha ,\alpha _{\sigma (1)},\ldots ,\alpha _{\sigma (m)}},\quad \Lambda _{{\underline{\alpha }},{\underline{\beta }}}:=\sum _{\alpha \in {\underline{\alpha }}}\Lambda _{\alpha ,{\underline{\alpha }}\cup {\underline{\beta }}{\setminus }\{\alpha \}},\\ {\underline{\beta }}_{<k}^k&:=\bigsqcup _{j<k} {\underline{\beta }}_j^k, \quad {\underline{\beta }}_{>k}^k:=\bigsqcup _{j>k} {\underline{\beta }}_j^k. \end{aligned} \end{aligned}$$

Some notations in (4.3) require further explanation. The qualifier “if $\sum _{\alpha _k}$” is satisfied for those terms in which $\alpha _k$ is a summation variable when the brackets in the product $\prod _j(1+ \sum )$ are opened. The notation $\bigsqcup$ indicates the union of multisets.

For even *p* we apply (4.3) with $\Lambda ^{(k)}(D) :=\left\langle {{\,\mathrm{diag}\,}}({\mathbf {f}}{\mathbf {p}})D\right\rangle$ for $k\le p/2$ and $\Lambda ^{(k)}(D) :=\overline{\left\langle {{\,\mathrm{diag}\,}}({\mathbf {f}}{\mathbf {p}})D\right\rangle }$ for $k> p/2$. This is obviously a special case of $\Lambda ^{(k)}(D)=\left\langle BD\right\rangle$ which was considered in the so-called averaged case of [34] with arbitrary *B* of bounded operator norm since $\Vert {{\,\mathrm{diag}\,}}({\mathbf {f}}{\mathbf {p}})\Vert =\Vert {\mathbf {f}}{\mathbf {p}}\Vert _\infty \le C$. It was proved in [34] that

$$\begin{aligned} \left| \left\langle {{\,\mathrm{diag}\,}}({\mathbf {f}}{\mathbf {p}}) D\right\rangle \right| \lesssim \frac{\rho }{N\eta }, \end{aligned}$$

which is not good enough at the cusp. We can nevertheless use the graphical language developed in [34] to estimate the complicated right hand side of (4.3).

### Graphical representation via double index graphs

The graphs (or Feynman diagrams) introduced in [34] encode the structure of all terms in (4.3). Their (directed) edges correspond to resolvents *G*, while vertices correspond to $\Delta$’s. Loop edges are allowed while parallel edges are not. Resolvents *G* and their Hermitian conjugates $G^*$ are distinguished by different types of edges. Each vertex *v* carries a label $\alpha _v$ and we need to sum up for all labels. Some labels are independently summed up, these are the $\alpha$-labels in (4.3), while the $\beta$-labels are strongly restricted; in the independent case they can only be of the type $\alpha$ or $\alpha ^t$. These graphs will be called “double indexed” graphs since the vertices are naturally equipped with labels (double indices). Here we introduced the terminology “double indexed” for the graphs in [34] to distinguish them from the “single indexed” graphs to be introduced later in this paper.

To be more precise, the graphs in [34] were vertex-coloured graphs. The colours encoded a resummation of the terms in (4.3): vertices whose labels (or their transpose) appeared in one of the cumulants in (4.3) received the same colour. We then first summed up the colours and only afterwards we summed up all labels compatible with the given colouring. According to [34, Proposition 4.4] and the expansion of the main term [34, Eq. (49)] for every even *p* it holds that

4.4a $$\begin{aligned} {{\,\mathrm{\mathbf {E}}\,}}\left| \left\langle {{\,\mathrm{diag}\,}}({\mathbf {f}}{\mathbf {p}})D\right\rangle \right| ^p = \sum _{\Gamma \in \mathcal {G}^{\text {av}(p,6p)} }{{\,\mathrm{Val}\,}}(\Gamma )+{\mathcal {O}}\,\left( N^{-p}\right) , \end{aligned}$$

where $\mathcal {G}^{\text {av}(p,6p)}$ is a certain finite collection of vertex coloured directed graphs with *p* connected components, and ${{\,\mathrm{Val}\,}}(\Gamma )$, the value of the graph $\Gamma$, will be recalled below. According to [34] each graph $\Gamma \in \mathcal {G}^{\text {av}(p,6p)}$ fulfils the following properties:

#### Proposition 4.1

(Properties of double index graphs). There exists a finite set $\mathcal {G}^{\text {av}(p,6p)}$ of double index graphs $\Gamma$ such that (4.4) hold. Each $\Gamma$ fulfils the following properties.

- There exist exactly *p* connected components, all of which are oriented cycles. Each vertex has one incoming and one outgoing edge.
- Each connected component contains at least one vertex and one edge. Single vertices with a looped edge are in particular legal connected components.
- Each colour colours at least two and at most 6*p* vertices.
- If a colour colours exactly two vertices, then these vertices are in different connected components.
- The edges represent the resolvent matrix *G* or its adjoint $G^*$. Within each component either all edges represent *G* or all edges represent $G^*$. Accordingly we call the components either *G* or $G^*$-cycles.
- Within each cycle there is one designated edge which is represented as a wiggled line in the graph. The designated edge represents the matrix $G{{\,\mathrm{diag}\,}}({\mathbf {p}}{\mathbf {f}})$ in a *G*-cycle and the matrix ${{\,\mathrm{diag}\,}}({\mathbf {p}}{\mathbf {f}})G^*$ in a $G^*$-cycle.
- For each colour there exists at least one component in which a vertex of that colour is connected to the matrix ${{\,\mathrm{diag}\,}}({\mathbf {f}}{\mathbf {p}})$. According to (f) this means that if the relevant vertex is in a *G*-cycle, then the designated (wiggled) edge is its incoming edge. If the relevant vertex is in a *G*-cycle, then the designated edge is its outgoing edge.

If *V* is the vertex set of $\Gamma$ and for each colour $c\in C$, $V_c$ denotes the *c*-coloured vertices then we recall that

4.4b $$\begin{aligned} \begin{aligned} {{\,\mathrm{Val}\,}}(\Gamma )&= (-1)^{\left| V\right| } \Big (\prod _{c\in C} \prod _{v\in V_c} \sum _{\alpha _v} \frac{\kappa (\{\alpha _v\}_{v\in V_c})}{(\left| V_c\right| -1)!}\Big ) \\&\qquad \times {{\,\mathrm{\mathbf {E}}\,}}\prod _{\text {Cyc}(v_1,\ldots ,v_k)\in \Gamma } {\left\{ \begin{array}{ll} \left\langle G{{\,\mathrm{diag}\,}}({\mathbf {f}}{\mathbf {p}})\Delta ^{\alpha _{v_1}}G\ldots G\Delta ^{\alpha _{v_k}}\right\rangle \\ \left\langle \Delta ^{\alpha _{v_k}}G^*\ldots G^*\Delta ^{\alpha _{v_1}} {{\,\mathrm{diag}\,}}({\mathbf {f}}{\mathbf {p}})G^*\right\rangle \end{array}\right. } \end{aligned} \end{aligned}$$

where the ultimate product is the product over all *p* of the cycles in the graph. By the notation $\text {Cyc}(v_1,\ldots ,v_k)$ we indicate a directed cycle with vertices $v_1, \ldots , v_k$. Depending upon whether a given cycle is a *G*-cycle or $G^*$-cycle, it then contributes with one of the factors indicated after the last curly bracket in (4.4b) with the vertex order chosen in such a way that the designated edge represents the $G{{\,\mathrm{diag}\,}}({\mathbf {f}}{\mathbf {p}})$ or ${{\,\mathrm{diag}\,}}({\mathbf {f}}{\mathbf {p}})G^*$ matrix. As an example illustrating (4.4b) we have4.5Actually in [34] the graphical representation of the graph $\Gamma$ is simplified, it does not contain all information encoded in the graph. First, the direction of the edges are not indicated. In the picture both cycles should be oriented in a clockwise orientation. Secondly, the type of edges are not indicated, apart from the wiggled line. In fact, the edges in the second subgraph stand for $G^*$, while those in the first subgraph stand for *G*. To translate the pictorial representation directly let the striped vertices in the first and second cycle be associated with $\alpha _1,\beta _1$ and the dotted vertices with $\alpha _2,\beta _2$. Accordingly, the wiggled edge in the first cycle stands for $G{{\,\mathrm{diag}\,}}({\mathbf {f}}{\mathbf {p}})$, while the wiggled edge in the second cycle stands for ${{\,\mathrm{diag}\,}}({\mathbf {f}}{\mathbf {p}})G^*$. The reason why these details were omitted in the graphical representation of a double index graph is that they do not influence the basic power counting estimate of its value used in [34].

### Single index graphs

In [34] we operated with double index graphs that are structurally simple and appropriate for bookkeeping complicated correlation structures, but they are not suitable for detecting the additional smallness we need at the cusp. The contribution of the graphs in [34] were estimated by a relatively simple power counting argument where only the number of (typically off-diagonal) resolvent elements were recorded. In fact, for many subleading graphs this procedure already gave a very good bound that is sufficient at the cusps as well. The graphs carrying the leading contribution, however, have now to be computed to a higher accuracy and this leads to the concept of “single index graphs”. These are obtained by a certain refinement and reorganization of the double index graphs via a procedure we will call *graph resolution* to be defined later. The main idea is to restructure the double index graph in such a way that instead of labels (double indices) $\alpha =(a,b)$ its vertices naturally represent single indices *a* and *b*. Every double indexed graph will give rise to a finite number of resolved single index graphs. The double index graphs that require a more precise analysis compared with [34] will be resolved to single index graphs. After we explain the structure of the single index graphs and the graph resolution procedure, double index graphs will not be used in this paper any more. Thus, unless explicitly stated otherwise, by graph we will mean single index graph in the rest of this paper.

We now define the set $\mathcal {G}$ of single index graphs we will use in this paper. They are directed graphs, where parallel edges and loops are allowed. Let the graph be denoted by $\Gamma$ with vertex set $V(\Gamma )$ and edge set $E(\Gamma )$. We will assign a value to each $\Gamma$ which comprises weights assigned to the vertices and specific values assigned to the edges. Since an edge may represent different objects, we will introduce different types of edges that will be graphically distinguished by different line style. We now describe these ingredients precisely.

#### Vertices.

Each vertex $v\in V(\Gamma )$ is equipped with an associated index $a_v\in J$. Graphically the vertices are represented by small sunlabelled bullets  , i.e. in the graphical representation the actual index is not indicated. It is understood that all indices will be independently summed up over the entire index set *J* when we compute the value of the graph.

#### Vertex weights.

Each vertex $v\in V(\Gamma )$ carries some weight vector ${\mathbf {w}}^{(v)}\in \mathbb {C}^J$ which is evaluated ${\mathbf {w}}^{(v)}_{a_v}$ at the index $a_v$ associated with the vertex. We generally assume these weights to be uniformly bounded in *N*, i.e. $\sup _N\Vert {\mathbf {w}}^{(v)}\Vert _\infty <\infty$. Visually we indicate vertex weights by incoming arrows as in  . Vertices without explicitly indicated weight may carry an arbitrary bounded weight vector. We also use the notation  to indicate the constant ${\varvec{1}}$ vector as the weight, this corresponds to summing up the corresponding index unweighted

#### *G*-edges.

The set of *G*-edges is denoted by ${{\,\mathrm{GE}\,}}(\Gamma )\subset E(\Gamma )$. These edges describe resolvents and there are four types of *G*-edges. First of all, there are directed edges corresponding to *G* and $G^*$ in the sense that a directed *G* or $G^*$-edge $e=(v,u)\in E$ initiating from the vertex $v=i(e)$ and terminating in the vertex $u=t(e)$ represents the matrix elements $G_{a_va_u}$ or respectively $G^*_{a_va_u}$ evaluated in the indices $a_v,a_u$ associated with the vertices *v* and *u*. Besides these two there are also edges representing $G-M$ and $(G-M)^*$. Distinguishing between *G* and $G-M$, for practical purposes, is only important if it occurs in a loop. Indeed, $(G-M)_{aa}$ is typically much smaller than $G_{aa}$, while $(G-M)_{ab}$ basically acts just like $G_{ab}$ when *a*, *b* are summed independently. Graphically we will denote the four types of *G*-edges by 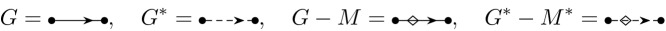


where all these edges can also be loops. The convention is that continuous lines represent *G*, dashed lines correspond to $G^*$, while the diamond on both types of edges indicates the subtraction of *M* or $M^*$. An edge $e\in {{\,\mathrm{GE}\,}}(\Gamma )$ carries its type as its attribute, so as a short hand notation we can simply write $G_e$ for $G_{a_{i(e)},a_{t(e)}}$, $G^*_{a_{i(e)},a_{t(e)}}$, $(G-M)_{a_{i(e)},a_{t(e)}}$ and $(G-M)^*_{a_{i(e)},a_{t(e)}}$ depending on which type of *G*-edge *e* represents. Due to their special role in the later estimates, we will separately bookkeep those $G-M$ or $G^*-M^*$ edges that appear looped. We thus define the subset ${{\,\mathrm{GE}\,}}_{g-m}\subset {{\,\mathrm{GE}\,}}$ as the set of *G*-edges $e\in {{\,\mathrm{GE}\,}}(\Gamma )$ of type $G-M$ or $G^*-M^*$ such that $i(e)=t(e)$. We write $g-m$ to refer to the fact that looped edges are evaluated on the diagonal $(g-m)_{a_v}$ of $(G-M)_{a_va_v}$.

#### (*G*-)edge degree.

For any vertex *v* we define its in-degree $\deg ^-(v)$ and out-degree $\deg ^+(v)$ as the number of incoming and outgoing *G*-edges. Looped edges (*v*, *v*) are counted for both in- and out-degree. We denote the total degree by $\deg (v)=\deg ^-(v)+\deg ^+(v)$.

#### Interaction edges.

Besides the *G*-edges we also have interaction edges, ${{\,\mathrm{IE}\,}}(\Gamma )$, representing the cumulants $\kappa$. A directed interaction edge $e=(u,v)$ represents the matrix $R^{(e)}=\big (r_{ab}^{(e)}\big )_{a,b\in J}$ given by the cumulant

4.6 $$\begin{aligned} r_{ab}^{(u,v)}= & {} \frac{1}{(\deg (u)-1)!}\kappa ( \underbrace{ab,\ldots ,ab}_{\deg ^-(u)\text { times}}, \underbrace{ba,\ldots ,ba}_{\deg ^+(u)\text { times}} )\nonumber \\= & {} \frac{1}{(\deg (v)-1)!}\kappa ( \underbrace{ab,\ldots ,ab}_{\deg ^+(v)\text { times}}, \underbrace{ba,\ldots ,ba}_{\deg ^-(v)\text { times}} ). \end{aligned}$$

For all graphs $\Gamma \in \mathcal {G}$ and all interaction edges $e=(u,v)$ we have the symmetries $\deg ^-(u)=\deg ^+(v)$ and $\deg ^-(v)=\deg ^+(u)$. Thus (4.6) is compatible with exchanging the roles of *u* and *v*. For the important case when $\deg (u)=\deg (v)=2$ it follows that the interaction from *u* to *v* is given by *S* if *u* has one incoming and one outgoing *G*-edge and *T* if *u* has two incoming *G*-edges, i.e.

$$\begin{aligned} s_{ab} = \kappa (ab,ba)\qquad t_{ab}= \kappa (ab,ab). \end{aligned}$$

Visually we will represent interaction edges as 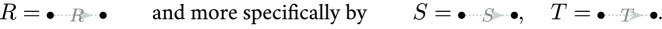


Although the interaction matrix $R^{(e)}$ is completely determined by the in- and out-degrees of the adjacent vertices *i*(*e*), *t*(*e*) we still write out the specific *S* and *T* names because these will play a special role in the latter part of the proof. As a short hand notation we shall frequently use $R_e:=R^{(e)}_{a_{i(e)},a_{t(e)}}$ to denote the matrix element selected by the indices $a_{i(e)},a_{t(e)}$ associated with the initial and terminal vertex of *e*. We also note that we do not indicate the direction of edges associated with *S* as the matrix *S* is symmetric.

#### Generic weighted edges.

Besides the specific *G*-edges and interaction edges, additionally we also allow for generic edges reminiscent of the generic vertex weights introduced above. They will be called *generic weighted edges*, or *weighted edges* for short. To every weighted edge *e* we assign a weight matrix $K^{(e)}=(k^{(e)}_{ab})_{a,b\in J}$ which is evaluated as $k^{(e)}_{a_{i(e)},a_{t(e)}}$ when we compute the value of the graph by summing up all indices. To simplify the presentation we will not indicate the precise form of the weight matrix $K^{(e)}$ but only its entry-wise scaling as a function of *N*. A weighted edge presented as  represents an arbitrary weight matrix $K^{(e)}$ whose entries scale like . We denote the set of weighted edges by ${{\,\mathrm{WE}\,}}(\Gamma )$. For a given weighted edge $e\in {{\,\mathrm{WE}\,}}$ we record the entry-wise scaling of $K^{(e)}$ in an exponent $l(e)\ge 0$ in such a way that we always have .

#### Graph value.

For graphs $\Gamma \in \mathcal {G}$ we define their value

4.7 $$\begin{aligned} {{\,\mathrm{Val}\,}}(\Gamma )&:=(-1)^{\left| {{\,\mathrm{GE}\,}}(\Gamma )\right| } \bigg (\prod _{v\in V(\Gamma )}\sum _{a_v\in J} {\mathbf {w}}^{(v)}_{a_v} \bigg ) \bigg (\prod _{e\in {{\,\mathrm{IE}\,}}(\Gamma )} r^{(e)}_{a_{i(e)},a_{t(e)}}\bigg )\bigg (\prod _{e\in {{\,\mathrm{WE}\,}}(\Gamma )} k^{(e)}_{a_{i(e)},a_{t(e)}}\bigg )\nonumber \\&\qquad \times {{\,\mathrm{\mathbf {E}}\,}}\bigg (\prod _{e\in {{\,\mathrm{GE}\,}}(\Gamma )} G_e\bigg ), \end{aligned}$$

which differs slightly from that in (4.4b) because it applies to a different class of graphs.

### Single index resolution

There is a natural mapping from double indexed graphs to a collection of single indexed graphs that encodes the rearranging of the terms in (4.4b) when the summation over labels $\alpha _v$ is reorganized into summation over single indices. Now we describe this procedure.

#### Definition 4.2

*(Single index resolution).* By the *single index resolution* of a double vertex graph we mean the collection of single index graphs obtained through the following procedure.

(i) For each colour, the identically coloured vertices of the double index graph are mapped into a pair of vertices of the single index graph.
(ii) The pair of vertices in the single index graph stemming from a fixed colour is connected by an interaction edge in the single index graph.
(iii) Every (directed) edge of the double index graph is naturally mapped to a *G*-edge of the single index graph. While mapping equally coloured vertices $x_1,\ldots ,x_k$ in the double index graph to vertices *u*, *v* connected by an interaction edge $e=(u,v)$ there are $k-1$ binary choices of whether we map the incoming edge of $x_j$ to an incoming edge of *u* and the outgoing edge of $x_j$ to an outgoing edge of *v* or vice versa. In this process we are free to consider the mapping of $x_1$ (or any other vertex, for that matter) as fixed by symmetry of $u\leftrightarrow v$.
(iv) If a wiggled *G*-edge is mapped to an edge from *u* to *v*, then *v* is equipped with a weight of ${\mathbf {p}}{\mathbf {f}}$. If a wiggled $G^*$-edge is mapped to an edge from *u* to *v*, then *u* is equipped with a weight of ${\mathbf {p}}{\mathbf {f}}$. All vertices with no weight specified in this process are equipped with the constant weight ${\varvec{1}}$.

We define the set $\mathcal {G}(p)\subset \mathcal {G}$ as the set of all graphs obtained from the double index graphs $\mathcal {G}^{\text {av}(p,6p)}$ via the single index resolution procedure.

#### Remark 4.3

(i) We note some ingredients described in Sect. 4.2 for a typical graph in $\mathcal {G}$ will be absent for graphs $\Gamma \in \mathcal {G}(p)\subset \mathcal {G}$. For example, ${{\,\mathrm{WE}\,}}(\Gamma )={{\,\mathrm{GE}\,}}_{g-m}(\Gamma )=\emptyset$ for all $\Gamma \in \mathcal {G}(p)$.
(ii) We also remark that loops in double index graphs are never mapped into loops in single index graphs along the single index resolution. Indeed, double index loops are always mapped to edges parallel to the interaction edge of the corresponding vertex.

A few simple facts immediately follow from the the single index construction in Definition 4.2. From (i) it is clear that the number of vertices in the single index graph is twice the number of colours of the double index graph. From (ii) it follows that the number of interaction edges in the single index graph equals the number of colours of the double index graph. Finally, from (iii) it is obvious that if for some colour *c* there are $k=k(c)$ vertices in the double index graph with colour *c*, then the resolution of this colour gives rise to $2^{k(c)-1}$ single indexed graph. Since these resolutions are done independently for each colour, we obtain that the number of single index graphs originating from one double index graph is

$$\begin{aligned} \prod _c 2^{k(c)-1} \end{aligned}$$

Since the number of double index graph in $\mathcal {G}^{\text {av}(p,6p)}$ is finite, so is the number of graphs in $\mathcal {G}(p)$.

Let us present an example of single index resolution applied to the graph from (4.5) where we, for the sake of transparency, label all vertices and edges. $\Gamma$ is a graph consisting of one 2-cycle on the vertices $x_1,y_2$ and one 2-cycle on the vertices $x_2,y_1$ as in4.8with $x_1,y_1$ and $x_2,y_2$ being of equal colour (i.e. being associated to labels connected through cumulants). In order to explain steps (i)–(iii) of the construction we first neglect that some edges may be wiggled, but we restore the orientation of the edges in the picture. We then fix the mapping of $x_i$ to pairs of vertices $(u_i,v_i)$ for $i=1,2$ in such a way that the incoming edges of $x_i$ are incoming at $u_i$ and the outgoing edges from $x_i$ are outgoing from $v_i$. It remains to map $y_i$ to $(u_i,v_i)$ and for each *i* there are two choices of doing so that we obtain the four possibilities 
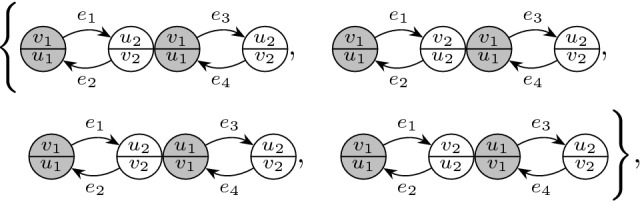


which translates to4.9in the language of single index graphs where the *S*, *T* assignment agrees with (4.6). Finally we want to visualize step (iv) in the single index resolution in our example. Suppose that in (4.8) the edges $e_1$ and $e_2$ are *G*-edges while $e_3$ and $e_4$ are $G^*$ edges with $e_2$ and $e_4$ being wiggled (in agreement with (4.5)). According to (iv) it follows that the terminal vertex of $e_2$ and the initial vertex of $e_4$ are equipped with a weight of ${\mathbf {p}}{\mathbf {f}}$ while the remaining vertices are equipped with a weight of ${\varvec{1}}$. The first graph in (4.9) would thus be equipped with the weights 
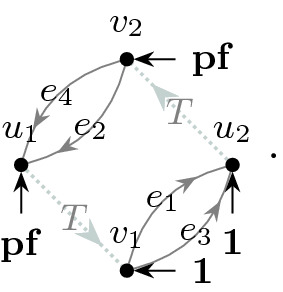


#### Single index graph expansion.

With the value definition in (4.7) it follows from Definition 4.2 that

4.10 $$\begin{aligned} {{\,\mathrm{\mathbf {E}}\,}}\left| \left\langle {{\,\mathrm{diag}\,}}({\mathbf {f}}{\mathbf {p}}) D\right\rangle \right| ^p = N^{-p} \sum _{\Gamma \in \mathcal {G}(p)} {{\,\mathrm{Val}\,}}(\Gamma ) + {\mathcal {O}}\,\left( N^{-p}\right) . \end{aligned}$$

We note that in contrast to the value definition for double index graphs (4.4), where each average in (4.4b) contains an 1/*N* prefactor, the single index graph value (4.7) does not include the $N^{-p}$ prefactor. We chose this convention in this paper mainly because the exponent *p* in the prefactor $N^{-p}$ cannot be easily read off from the single index graph itself, whereas in the double index graph *p* is simply the number of connected components.

We now collect some simple facts about the structure of these graphs in $\mathcal {G}(p)$ which directly follow from the corresponding properties of the double index graphs listed in Proposition 4.1.

##### Fact 1

The interaction edges ${{\,\mathrm{IE}\,}}(\Gamma )$ form a perfect matching of $\Gamma$, in particular $\left| V\right| =2\left| {{\,\mathrm{IE}\,}}\right|$. Moreover, $1\le \left| {{\,\mathrm{IE}\,}}(\Gamma )\right| \le p$ and therefore the number of vertices in the graph is even and satisfies $2\le \left| V(\Gamma )\right| \le 2p$. Finally, since for $(u,v)\in {{\,\mathrm{IE}\,}}$ we have $\deg ^-(u) = \deg ^+(v)$ and $\deg ^-(v)=\deg ^+(u)$, consequently also $\deg (e):=\deg (u)=\deg (v)$. The degree furthermore satisfies the bounds $2\le \deg (e)\le 6p$ for each $e\in {{\,\mathrm{IE}\,}}(\Gamma )$.

##### Fact 2

The weights associated with the vertices are some non-negative powers of ${\mathbf {f}}{\mathbf {p}}$ in such a way that the total power of all ${\mathbf {f}}{\mathbf {p}}$’s is exactly *p*. The trivial zeroth power, i.e. the constant weight ${\varvec{1}}$ is allowed. Furthermore, the ${\mathbf {f}}{\mathbf {p}}$ weights are distributed in such a way that at least one non-trivial ${\mathbf {f}}{\mathbf {p}}$ weight is associated with each interacting edge $(u,v)=e\in {{\,\mathrm{IE}\,}}(\Gamma )$.

### Examples of graphs

We now turn to some examples explaining the relation of the double index graphs from [34] and single index graphs. We note that the single index graphs actually contain more information because they specify edge direction, specify weights explicitly and differentiate between *G* and $G^*$ edges. These information were not necessary for the power counting arguments used in [34], but for the improved estimates they will be crucial.

We start with the graphs representing the following simple equality following from $\kappa (\alpha ,\beta )={{\,\mathrm{\mathbf {E}}\,}}w_\alpha w_\beta$

$$\begin{aligned} \begin{aligned}&N^2{{\,\mathrm{\mathbf {E}}\,}}\sum _{\alpha ,\beta } \kappa (\alpha ,\beta ) \left\langle {{\,\mathrm{diag}\,}}({\mathbf {f}}{\mathbf {p}}) \Delta ^{\alpha } G\right\rangle \left\langle G^*\Delta ^\beta {{\,\mathrm{diag}\,}}({\mathbf {f}}{\mathbf {p}})^*\right\rangle \\&\quad = \sum _{a,b} s_{ab} (pf)_a^2 {{\,\mathrm{\mathbf {E}}\,}}G_{ba} G^*_{ab} + \sum _{a,b} t_{ab} (pf)_a (pf)_b {{\,\mathrm{\mathbf {E}}\,}}G_{ba} G^*_{ba} \end{aligned} \end{aligned}$$

which can be represented as 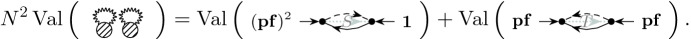


We now turn to the complete graphical representation for the second moment in the case of Gaussian entries,4.11where we again stress that the double index graphs hide the specific weights and the fact that one of the connected components actually contains $G^*$ edges. In terms of single index graphs, the rhs. of (4.11) can be represented as the sum over the values of the six graphs4.12The first two graphs were already explained above. The additional four graphs come from the second term in the rhs. of (4.11). Since $\kappa (\alpha _1, \beta _1)$ is non-zero only if $\alpha _1=\beta _1$ or $\alpha _1=\beta _1^t$, there are four possible choices of relations among the $\alpha$ and $\beta$ labels in the two kappa factors. For example, the first graph in the second line of (4.12) corresponds to the choice $\alpha _1^t = \beta _1$, $\alpha _2^t=\beta _2$. Written out explicitly with summation over single indices, this value is given by

$$\begin{aligned} \sum _{a_1,b_1}\sum _{a_2,b_2} (pf)_{a_1} (pf)_{b_2} s_{a_1b_1} s_{a_2b_2} {{\,\mathrm{\mathbf {E}}\,}}G_{a_2a_1} G_{b_1 b_2} G^*_{a_1a_2} G^*_{b_2b_1} \end{aligned}$$

where in the picture the left index corresponds to $a_1$, the top index to $b_2$, the right one to $a_2$ and the bottom one to $b_1$.

We conclude this section by providing an example of a graph with some degree higher than two which only occurs in the non-Gaussian situation and might contain looped edges. For example, in the expansion of $N^2{{\,\mathrm{\mathbf {E}}\,}}\left| \left\langle {{\,\mathrm{diag}\,}}({\mathbf {f}}{\mathbf {p}}) D\right\rangle \right| ^2$ in the non-Gaussian setup there is the term 
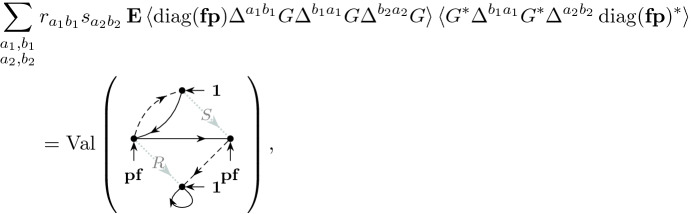


where $r_{ab}=\kappa (ab,ba,ba)/2$ and $s_{ab}=\kappa (ab,ba)$, in accordance with (4.6).

### Simple estimates on ${{\,\mathrm{Val}\,}}(\Gamma )$

In most cases we aim only at estimating the value of a graph instead of precisely computing it. The simplest power counting estimate on (4.7) uses that the matrix elements of *G* and those of the generic weight matrix *K* are bounded by an ${\mathcal {O}}\,\left( 1\right)$ constant, while the matrix elements of $R^{(e)}$ are bounded by $N^{-\deg (e)/2}$. Thus the naive estimate on (4.7) is

4.13 $$\begin{aligned} \left| {{\,\mathrm{Val}\,}}(\Gamma )\right| \lesssim \Big (\prod _{v\in V(\Gamma )} N\Big )\Big (\prod _{e\in {{\,\mathrm{IE}\,}}(\Gamma )} N^{-\deg (e)/2}\Big ) = \prod _{e\in {{\,\mathrm{IE}\,}}(\Gamma )} N^{2-\deg (e)/2} \le \prod _{e\in {{\,\mathrm{IE}\,}}(\Gamma )} N\le N^{p} \nonumber \\ \end{aligned}$$

where we used that the interaction edges form a perfect matching and that $\deg (e)\ge 2$, $\left| {{\,\mathrm{IE}\,}}(\Gamma )\right| \le p$. The somewhat informal notation $\lesssim$ in (4.13) hides a technical subtlety. The resolvent entries $G_{ab}$ are indeed bounded by an ${\mathcal {O}}\,\left( 1\right)$ constant in the sense of very high moments but not almost surely. We will make bounds like the one in (4.13) rigorous in a high moments sense in Lemma 4.8.

The estimate (4.13) ignores the fact that typically only the diagonal resolvent matrix elements of *G* are of ${\mathcal {O}}\,\left( 1\right)$, the off-diagonal matrix elements are much smaller. This is manifested in the *Ward-identity*

4.14a $$\begin{aligned} \sum _{a\in J} \left| G_{ab}\right| ^2 = (G^*G)_{bb} = \frac{(G-G^*)_{bb}}{2i\eta }= \frac{\mathfrak {I}G_{bb}}{\eta }. \end{aligned}$$

Thus the sum of off-diagonal resolvent elements $G_{ab}$ is usually smaller than its naive size of order *N*, at least in the regime $\eta \gg N^{-1}$. This is quantified by the so called Ward estimates

4.14b $$\begin{aligned} \sum _{a\in J} \left| G_{ab}\right| ^2 = N\frac{\mathfrak {I}G_{bb}}{N\eta } \lesssim N \psi ^2,\qquad \sum _{a\in J} \left| G_{ab}\right| \lesssim N\psi , \qquad \psi :=\left( \frac{\rho }{N\eta }\right) ^{1/2}.\nonumber \\ \end{aligned}$$

Similarly to (4.13) the inequalities $\lesssim$ in (4.14b) are meant in a power counting sense ignoring that the entries of $\mathfrak {I}G$ might not be bounded by $\rho$ almost surely but only in some high moment sense.

As a consequence of (4.14b) we can gain a factor of $\psi$ for each off-diagonal (that is, connecting two separate vertices) *G*-factor, but clearly only for at most two *G*-edges per adjacent vertex. Moreover, this gain can obviously only be used once for each edge and not twice, separately when summing up the indices at both adjacent vertices. As a consequence a careful counting of the total number of $\psi$-gains is necessary, see [34, Section 4.3] for details.

**Ward bounds for the example graphs from Sect.** 4.4. From the single index graphs drawn in (4.12) we can easily obtain the known bound ${{\,\mathrm{\mathbf {E}}\,}}\left| \left\langle {{\,\mathrm{diag}\,}}({\mathbf {f}}{\mathbf {p}})D\right\rangle \right| ^2\lesssim \psi ^4$. Indeed, the last four graphs contribute a combinatorial factor of $N^4$ from the summations over four single indices and a scaling factor of $N^{-2}$ from the size of *S*, *T*. Furthermore, we can gain a factor of $\psi$ for each *G*-edge through Ward estimates and the bound follows. Similarly, the first two graphs contribute a factor of $N=N^{2-1}$ from summation and *S*/*T* and a factor of $\psi ^2$ from the Ward estimates, which overall gives $N^{-1}\psi ^2\lesssim \psi ^4$. As this example shows, the bookkeeping of available Ward-estimates is important and we will do so systematically in the following sections.

### Improved estimates on ${{\,\mathrm{Val}\,}}(\Gamma )$: Wardable edges

For the sake of transparency we briefly recall the combinatorial argument used in [34], which also provides the starting point for the refined estimate in the present paper. Compared to [34], however, we phrase the counting argument directly in the language of the single index graphs. We only aim to gain from the *G*-edges adjacent to vertices of degree two or three; for vertices of higher degree the most naive estimate $\left| G_{ab}\right| \lesssim 1$ is already sufficient as demonstrated in [34]. We collect the vertices of degree two and three in the set $V_{2,3}$ and collect the *G*-edges adjacent to $V_{2,3}$ in the set $E_{2,3}$. In [34, Section 4.3] a specific *marking procedure* on the *G*-edges of the graph is introduced that has the following properties. For each $v\in V_{2,3}$ we put a *mark* on at most two adjacent *G*-edges in such a way that those edges can be estimated via (4.14b) while performing the $a_v$ summation. In this case we say that the mark comes from the *v*-perspective. An edge may have two marks coming from the perspective of each of its adjacent vertices. Later, marked edges will be estimated via (4.14b) while summing up $a_v$. After doing this for all of $V_{2,3}$ we call an edge in $E_{2,3}$*marked effectively* if it either *(i)* has two marks, or *(ii)* has one mark and is adjacent to only one vertex from $V_{2,3}$. While subsequently using (4.14b) in the summation of $a_v$ for $v\in V_{2,3}$ (in no particular order) on the marked edges (and estimating the remaining edges adjacent to *v* trivially) we can gain at least as many factors of $\psi$ as there are *effectively marked* edges. Indeed, this follows simply from the fact that *effectively marked* edges are never estimated trivially during the procedure just described, no matter the order of vertex summation.

#### Fact 3

For each $\Gamma \in \mathcal {G}(p)$ there is a marking of edges adjacent to vertices of degree at most 3 such that there are at least $\sum _{e\in {{\,\mathrm{IE}\,}}(\Gamma )} (4-\deg (e))_+$ effectively marked edges.

#### Proof

On the one hand we find from Fact 1 (more specifically, from the equality $\deg (e)=\deg (u)=\deg (v)$ for $(u,v)=e\in {{\,\mathrm{IE}\,}}(\Gamma )$) that

4.15 $$\begin{aligned} \left| E_{2,3}\right| \ge \sum _{v\in V_{2,3}} \frac{1}{2}\deg (v)=\sum _{e\in {{\,\mathrm{IE}\,}}(\Gamma ),\deg (e)\in \{2,3\}} \deg (e). \end{aligned}$$

On the other hand it can be checked that for every pair $(u,v)=e\in {{\,\mathrm{IE}\,}}(\Gamma )$ with $\deg (e)=2$ all *G*-edges adjacent to *u* or *v* can be marked from the *u*, *v*-perspective. Indeed, this is a direct consequence of Proposition 4.1(d): Because the two vertices in the double index graph being resolved to (*u*, *v*) cannot be part of the same cycle it follows that all of the (two, three or four) *G*-edges adjacent to the vertices with index *u* or *v* are not loops (i.e. do not represent diagonal resolvent elements). Therefore they can be bounded by using (4.14b). Similarly, it can be checked that for every edge $(u,v)=e\in {{\,\mathrm{IE}\,}}(\Gamma )$ with $\deg (e)=3$ at most two *G*-edges adjacent to *u* or *v* can remain unmarked from the *u*, *v*-perspective. By combining these two observations it follows that at most

4.16 $$\begin{aligned} \sum _{e\in {{\,\mathrm{IE}\,}}(\Gamma ),\deg (e)\in \{2,3\}} (2\deg (e)-4) \end{aligned}$$

edges in $E_{2,3}$ are *ineffectively marked* since those are counted as unmarked from the perspective of one of its vertices. Subtracting (4.16) from (4.15) it follows that in total at least

$$\begin{aligned} \sum _{e\in {{\,\mathrm{IE}\,}}(\Gamma )} (4-\deg (e))_+ = \sum _{e\in {{\,\mathrm{IE}\,}}(\Gamma ),\deg (e)\in \{2,3\}} (4-\deg (e)) \end{aligned}$$

edges are marked effectively, just as claimed. $\square$

In [34] it was sufficient to estimate the value of each graph in $\mathcal {G}(p)$ by subsequently estimating all effectively marked edges using (4.14b). For the purpose of improving the local law at the cusp, however, we need to introduce certain operations on the graphs of $\mathcal {G}(p)$ which allow to estimate the graph value to a higher accuracy. It is essential that during those operations we keep track of the number of edges we estimate using (4.14b). Therefore we now introduce a more flexible way of recording these edges. We first recall a basic definition [58] from graph theory.

#### Definition 4.4

For $k\ge 1$ a graph $\Gamma =(V,E)$ is called *k*-*degenerate* if any induced subgraph has minimal degree at most *k*.

It is well known that being *k*-degenerate is equivalent to the following sequential property.^2^ We provide a short proof for convenience.

#### Lemma 4.5

A graph $\Gamma =(V,E)$ is *k*-degenerate if and only if there exists an ordering of vertices $\{v_1,\ldots ,v_n\}=V$ such that for each $m\in [n]:=\{1,\ldots ,n\}$ it holds that

4.17 $$\begin{aligned} \deg _{\Gamma [\{v_1,\ldots ,v_m\}]}(v_m)\le k \end{aligned}$$

where for $V'\subset V$, $\Gamma [V']$ denotes the induced subgraph on the vertex set $V'$.

#### Proof

Suppose the graph is *k*-degenerate and let $n:=\left| V\right|$. Then there exists some vertex $v_n\in V$ such that $\deg (v_n)\le k$ by definition. We now consider the subgraph induced by $V':=V{\setminus }\{v_n\}$ and, by definition, again find some vertex $v_{n-1}\in V'$ of degree $\deg _{\Gamma [V']}(v_{n-1})\le k$. Continuing inductively we find a vertex ordering with the desired property.

Conversely, assume there exists a vertex ordering such that (4.17) holds for each *m*. Let $V'\subset V$ be an arbitrary subset and let $m:=\max \{l\in [n]|v_l\in V'\}$. Then it holds that

$$\begin{aligned} \deg _{\Gamma [V']}(v_m)\le \deg _{\Gamma [\{v_1,\ldots ,v_m\}]}(v_m)\le k \end{aligned}$$

and the proof is complete. $\square$

The reason for introducing this graph theoretical notion is that it is equivalent to the possibility of estimating edges effectively using (4.14b). A subset ${{\,\mathrm{GE}\,}}'$ of *G*-edges in $\Gamma \in \mathcal {G}$ can be fully estimated using (4.14b) if and only if there exists a vertex ordering such that we can subsequently remove vertices in such a way that in each step at most two edges from ${{\,\mathrm{GE}\,}}'$ are removed. Due to Lemma 4.5 this is the case if and only if $\Gamma '=(V,{{\,\mathrm{GE}\,}}')$ is 2-degenerate. For example, the graph $\Gamma _{\text {eff}}=(V,{{\,\mathrm{GE}\,}}_{\text {eff}})$ induced by the effectively marked *G*-edges ${{\,\mathrm{GE}\,}}_{\text {eff}}$ is a 2-degenerate graph. Indeed, each effectively marked edge is adjacent to at least one vertex which has degree at most 2 in $\Gamma _{\text {eff}}$: Vertices of degree 2 in $(V,{{\,\mathrm{GE}\,}})$ are trivially at most of degree 2 in $\Gamma _{\text {eff}}$, and vertices of degree 3 in $(V,{{\,\mathrm{GE}\,}})$ are also at most of degree 2 in $\Gamma _{\text {eff}}$ as they can only be adjacent to 2 effectively marked edges. Consequently any induced subgraph of $\Gamma _{\text {eff}}$ has to contain some vertex of degree at most 2 and thereby $\Gamma _{\text {eff}}$ is 2-degenerate.

#### Definition 4.6

For a graph $\Gamma =(V,{{\,\mathrm{GE}\,}}\cup {{\,\mathrm{IE}\,}}\cup {{\,\mathrm{WE}\,}})\in \mathcal {G}$ we call a subset of *G*-edges ${{\,\mathrm{{{\,\mathrm{GE}\,}}_{\text {W}}}\,}}\subset {{\,\mathrm{GE}\,}}$*Wardable* if the subgraph $(V,{{\,\mathrm{{{\,\mathrm{GE}\,}}_{\text {W}}}\,}})$ is 2-degenerate.

#### Lemma 4.7

For each $\Gamma \in \mathcal {G}(p)$ there exists a Wardable subset ${{\,\mathrm{{{\,\mathrm{GE}\,}}_{\text {W}}}\,}}\subset {{\,\mathrm{GE}\,}}$ of size

4.18 $$\begin{aligned} \left| {{\,\mathrm{GE}\,}}_W\right| = \sum _{e\in {{\,\mathrm{IE}\,}}} (4-\deg (e))_+. \end{aligned}$$

#### Proof

This follows immediately from Fact 3, the observation that $(V,{{\,\mathrm{GE}\,}}_{\text {eff}})$ is 2-degenerate and the fact that sub-graphs of 2-degenerate graphs remain 2-degenerate.

$$\square $$

For each $\Gamma \in \mathcal {G}(p)$ we choose a Wardable subset ${{\,\mathrm{{{\,\mathrm{GE}\,}}_{\text {W}}}\,}}(\Gamma )\subset {{\,\mathrm{GE}\,}}(\Gamma )$ satisfying (4.18). At least one such set is guaranteed to exist by the lemma. For graphs with several possible such sets, we arbitrarily choose one, and consider it permanently assigned to $\Gamma$. Later we will introduce certain operations on graphs $\Gamma \in \mathcal {G}(p)$ which produce families of derived graphs $\Gamma '\in \mathcal {G}\supset \mathcal {G}(p)$. During those operations the chosen Wardable subset ${{\,\mathrm{{{\,\mathrm{GE}\,}}_{\text {W}}}\,}}(\Gamma )$ will be modified in order to produce eligible sets of Wardable edges ${{\,\mathrm{{{\,\mathrm{GE}\,}}_{\text {W}}}\,}}(\Gamma ')$ and we will select one among those to define the Wardable subset of $\Gamma '$. We stress that the relation (4.18) on the Wardable set is required only for $\Gamma \in \mathcal {G}(p)$ but not for the derived graphs $\Gamma '$.

We now give a precise meaning to the vague bounds of (4.13), (4.14b). We define the *N*-exponent, $n(\Gamma )$, of a graph $\Gamma =(V,{{\,\mathrm{GE}\,}}\cup {{\,\mathrm{IE}\,}}\cup {{\,\mathrm{WE}\,}})$ as the effective *N*-exponent in its value-definition, i.e. as

$$\begin{aligned} n(\Gamma ):=\left| V\right| - \sum _{e\in {{\,\mathrm{IE}\,}}} \frac{\deg (e)}{2} - \sum _{e\in {{\,\mathrm{WE}\,}}} l(e). \end{aligned}$$

We defer the proof of the following technical lemma to the Appendix.

#### Lemma 4.8

For any $c>0$ there exists some $C>0$ such that the following holds. Let $\Gamma =(V,{{\,\mathrm{GE}\,}}\cup {{\,\mathrm{IE}\,}}\cup {{\,\mathrm{WE}\,}})\in \mathcal {G}$ be a graph with Wardable edge set ${{\,\mathrm{{{\,\mathrm{GE}\,}}_{\text {W}}}\,}}\subset {{\,\mathrm{GE}\,}}$ and at most $\left| V\right| \le c p$ vertices and at most $\left| {{\,\mathrm{GE}\,}}\right| \le c p^2$*G*-edges. Then for each $0<\epsilon <1$ it holds that

4.19a $$\begin{aligned} \left| {{\,\mathrm{Val}\,}}(\Gamma )\right| \le _\epsilon N^{\epsilon p} \big (1+\Vert G\Vert _q\big )^{Cp^2} {{\,\mathrm{W-Est}\,}}(\Gamma ), \end{aligned}$$

where

4.19b $$\begin{aligned} {{\,\mathrm{W-Est}\,}}(\Gamma ):=N^{n(\Gamma )} \big (\psi +\psi _q'\big )^{\left| {{\,\mathrm{{{\,\mathrm{GE}\,}}_{\text {W}}}\,}}\right| } \big (\psi +\psi '_q+\psi _q''\big )^{\left| {{\,\mathrm{GE}\,}}_{g-m}\right| }, \qquad q:=C p^3/\epsilon .\nonumber \\ \end{aligned}$$

#### Remark 4.9

(i) We consider $\epsilon$ and *p* as fixed within the proof of Theorem 3.7 and therefore do not explicitly carry the dependence of them in quantities like ${{\,\mathrm{W-Est}\,}}$.
(ii) We recall that the factors involving ${{\,\mathrm{GE}\,}}_{g-m}$ and ${{\,\mathrm{WE}\,}}$ do not play any role for graphs $\Gamma \in \mathcal {G}(p)$ as those sets are empty in this restricted class of graphs (see Remark 4.3).
(iii) Ignoring the difference between $\psi$ and $\psi _q'$, $\psi _q''$ and the irrelevant order ${\mathcal {O}}\,\left( N^{p\epsilon }\right)$ factor in (4.19), the reader should think of (4.19) as the heuristic inequality
  $$\begin{aligned} \left| {{\,\mathrm{Val}\,}}(\Gamma )\right| \lesssim N^{n(\Gamma )} \psi ^{\left| {{\,\mathrm{{{\,\mathrm{GE}\,}}_{\text {W}}}\,}}\right| +\left| {{\,\mathrm{GE}\,}}_{g-m}\right| }. \end{aligned}$$
  Using Lemma 4.7, $N^{-1/2}\lesssim \psi \lesssim 1$, $\left| V\right| =2\left| {{\,\mathrm{IE}\,}}\right| \le 2p$ and $\deg (e)\ge 2$ (from Fact 1) we thus find
  4.20 $$\begin{aligned} \begin{aligned} N^{-p} \left| {{\,\mathrm{Val}\,}}(\Gamma )\right|&\lesssim N^{\left| {{\,\mathrm{IE}\,}}\right| -p} \prod _{e\in {{\,\mathrm{IE}\,}}} N^{1-\deg (e)/2} \psi ^{(4-\deg (e))_+}\\&\lesssim \psi ^{2\left| {{\,\mathrm{IE}\,}}\right| -2p} \prod _{e\in {{\,\mathrm{IE}\,}}} \psi ^{\deg (e)-2+(4-\deg (e))_+}\le \psi ^{2p} \end{aligned} \end{aligned}$$
  for any $$\Gamma =(V,{{\,\mathrm{GE}\,}}\cup {{\,\mathrm{IE}\,}})\in \mathcal {G}(p)$$.

### Improved estimates on ${{\,\mathrm{Val}\,}}(\Gamma )$ at the cusp: $\sigma$-cells

#### Definition 4.10

For $\Gamma \in \mathcal {G}$ we call an interaction edge $(u,v)=e\in {{\,\mathrm{IE}\,}}(\Gamma )$ a $\sigma$-cell if the following four properties hold: (i) $\deg (e)=2$, (ii) there are no *G*-loops adjacent to *u* or *v*, (iii) precisely one of *u*, *v* carries a weight of ${\mathbf {p}}{\mathbf {f}}$ while the other carries a weight of ${\varvec{1}}$, and (iv), *e* is not adjacent to any other non ${{\,\mathrm{GE}\,}}$-edges. Pictorially, possible $\sigma$-cells are given by 
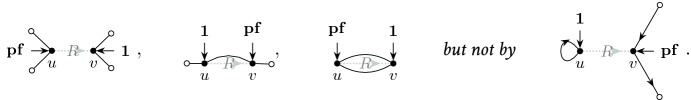


For $\Gamma \in \mathcal {G}$ we denote the number of $\sigma$-cells in $\Gamma$ by $\sigma (\Gamma )$.

Next, we state a simple lemma, estimating ${{\,\mathrm{W-Est}\,}}(\Gamma )$ of the graphs in the restricted class $\Gamma \in \mathcal {G}(p)$.

#### Lemma 4.11

For each $\Gamma =(V,{{\,\mathrm{IE}\,}}\cup {{\,\mathrm{GE}\,}})\in \mathcal {G}(p)$ it holds that

$$\begin{aligned} N^{-p}\left| {{\,\mathrm{W-Est}\,}}(\Gamma )\right| \le _p \Big (\sqrt{\eta /\rho }\Big )^{p-\sigma (\Gamma )}(\psi +\psi '_q)^{2p} \prod _{\begin{array}{c} e\in {{\,\mathrm{IE}\,}}\\ \deg (e)\ge 4 \end{array}} N^{2-\deg (e)/2}. \end{aligned}$$

#### Proof

We introduce the short-hand notations ${{\,\mathrm{IE}\,}}_k:=\{e\in {{\,\mathrm{IE}\,}}|\deg (e)=k\}$ and ${{\,\mathrm{IE}\,}}_{\ge k}:=\bigcup _{l\ge k}{{\,\mathrm{IE}\,}}_l$. Starting from (4.19b) and Lemma 4.7 we find

$$\begin{aligned} \begin{aligned}&N^{-p} \left| {{\,\mathrm{W-Est}\,}}(\Gamma )\right| \\&\quad \le N^{-(p-\left| {{\,\mathrm{IE}\,}}\right| )}\Bigg (\prod _{e\in {{\,\mathrm{IE}\,}}_2} (\psi +\psi '_q)^2\Bigg ) \Bigg (\prod _{e\in {{\,\mathrm{IE}\,}}_3} \frac{\psi +\psi '_q}{\sqrt{N}}\Bigg ) \Bigg (\prod _{e\in {{\,\mathrm{IE}\,}}_{\ge 4}} \frac{1}{N}\Bigg ) \Bigg (\prod _{e\in {{\,\mathrm{IE}\,}}_{\ge 4}} N^{2-\deg (e)/2}\Bigg ). \end{aligned} \end{aligned}$$

Using $N^{-1/2}= \psi \sqrt{\eta /\rho } \le C\psi$ it then follows that

4.21 $$\begin{aligned} \begin{aligned}&N^{-p}\left| {{\,\mathrm{W-Est}\,}}(\Gamma )\right| \\&\quad \le _p \bigg [\frac{\eta }{\rho } \psi ^2\bigg ]^{p-\left| {{\,\mathrm{IE}\,}}\right| }\Bigg (\prod _{e\in {{\,\mathrm{IE}\,}}_2} (\psi +\psi _q')^2\Bigg ) \Bigg (\prod _{e\in {{\,\mathrm{IE}\,}}_{\ge 3}} \sqrt{\frac{\eta }{\rho }}(\psi +\psi _q')^2\Bigg )\Bigg (\prod _{e\in {{\,\mathrm{IE}\,}}_{\ge 4}} N^{2-\deg (e)/2}\Bigg ). \end{aligned}\nonumber \\ \end{aligned}$$

It remains to relate (4.21) to the number $\sigma (\Gamma )$ of $\sigma$-cells in $\Gamma$. Since each interaction edge of degree two which is not a $\sigma$-cell has an additional weight ${\mathbf {p}}{\mathbf {f}}$ attached to it, it follows from Fact 2 that $\left| {{\,\mathrm{IE}\,}}_2\right| -\sigma (\Gamma )\le p - \left| {{\,\mathrm{IE}\,}}\right|$. Therefore, from (4.21) and $\eta /\rho \le C$ we have that

$$\begin{aligned} \begin{aligned}&N^{-p} \left| {{\,\mathrm{W-Est}\,}}(\Gamma )\right| \\&\quad \le _p \Big [\sqrt{\eta /\rho }(\psi +\psi '_q)^2\Big ]^{p-\left| {{\,\mathrm{IE}\,}}\right| +\left| {{\,\mathrm{IE}\,}}_{\ge 3}\right| +\left| {{\,\mathrm{IE}\,}}_2\right| -\sigma (\Gamma )} \Big [(\psi +\psi '_q)^2\Big ]^{\sigma (\Gamma )} \Bigg (\prod _{e\in {{\,\mathrm{IE}\,}}_{\ge 4}} N^{2-\deg (e)/2}\Bigg ), \end{aligned} \end{aligned}$$

proving the claim. $\square$

Using Lemma 4.8 and $\sqrt{\eta /\rho }\le \sigma _q$, the estimate in Lemma 4.11 has improved the previous bound (4.20) by a factor $\sigma _q^{p-\sigma (\Gamma )}$ (ignoring the irrelevant factors). In order to prove (3.11c), we thus need to remove the $-\sigma (\Gamma )$ from this exponent, in other words, we need to show that from each $\sigma$-cell we can multiplicatively gain a factor of $\sigma _q$. This is the content of the following proposition.

#### Proposition 4.12

Let $c>0$ be any constant and $\Gamma \in \mathcal {G}$ be a single index graph with at most *cp* vertices and $cp^2$ edges with a $\sigma$-cell $(u,v)=e\in {{\,\mathrm{IE}\,}}(\Gamma )$. Then there exists a finite collection of graphs $\{\Gamma _\sigma \}\sqcup \mathcal {G}_\Gamma$ with at most one additional vertex and at most 6*p* additional *G*-edges such that

4.22 $$\begin{aligned} \begin{aligned} {{\,\mathrm{Val}\,}}(\Gamma )&= \sigma {{\,\mathrm{Val}\,}}(\Gamma _\sigma )+\sum _{\Gamma '\in \mathcal {G}_\Gamma }{{\,\mathrm{Val}\,}}(\Gamma ') + {\mathcal {O}}\,\left( N^{-p}\right) ,\\ {{\,\mathrm{W-Est}\,}}(\Gamma _\sigma )&= {{\,\mathrm{W-Est}\,}}(\Gamma ),\qquad {{\,\mathrm{W-Est}\,}}(\Gamma ')\le _p \sigma _q{{\,\mathrm{W-Est}\,}}(\Gamma ), \quad \Gamma '\in \mathcal {G}_\Gamma \end{aligned} \end{aligned}$$

and all graphs $\Gamma _\sigma$ and $\Gamma '\in \mathcal {G}_\Gamma$ have exactly one $\sigma$-cell less than $\Gamma$.

Using Lemmas 4.8 and 4.11 together with the repeated application of Proposition 4.12 we are ready to present the proof of Theorem 3.7.

#### Proof of Theorem 3.7

We remark that the isotropic local law (3.11a) and the averaged local law (3.11b) are verbatim as in [34, Theorem 4.1]. We therefore only prove the improved bound (3.11c)–(3.11d) in the remainder of the section. We recall (4.10) and partition the set of graphs $\mathcal {G}(p)=\mathcal {G}_0(p)\cup \mathcal {G}_{\ge 1}(p)$ into those graphs $\mathcal {G}_0(p)$ with no $\sigma$-cells and those graphs $\mathcal {G}_{\ge 1}(p)$ with at least one $\sigma$-cell. For the latter group we then use Proposition 4.12 for some $\sigma$-cell to find

4.23 $$\begin{aligned} \begin{aligned} {{\,\mathrm{\mathbf {E}}\,}}\left| \left\langle {{\,\mathrm{diag}\,}}({\mathbf {p}}{\mathbf {f}})D\right\rangle \right| ^p&= N^{-p}\sum _{\Gamma \in \mathcal {G}_0(p)}{{\,\mathrm{Val}\,}}(\Gamma ) +{\mathcal {O}}\,\left( N^{-2p}\right) \\&\quad +N^{-p}\sum _{\Gamma \in \mathcal {G}_{\ge 1}(p)}\left( \sigma {{\,\mathrm{Val}\,}}(\Gamma _\sigma )+ \sum _{\Gamma '\in \mathcal {G}_\Gamma }{{\,\mathrm{Val}\,}}(\Gamma ') \right) , \end{aligned} \end{aligned}$$

where the number of $\sigma$-cells is reduced by 1 for $\Gamma _\sigma$ and each $\Gamma '\in \mathcal {G}_\Gamma$ as compared to $\Gamma$. We note that the Ward-estimate ${{\,\mathrm{W-Est}\,}}(\Gamma )$ from Lemma 4.11 together with Lemma 4.8 is already sufficient for the graphs in $\mathcal {G}_0(p)$. For those graphs $\mathcal {G}_1(p)$ with exactly one $\sigma$-cell the expansion in (4.23) is sufficient because $\sigma \le \sigma _q$ and, according to (4.22), each $\Gamma '\in \mathcal {G}_{\Gamma }$ has a Ward estimate which is already improved by $\sigma _q$. For the other graphs we iterate the expansion from Proposition 4.12 until no sigma cells are left.

It only remains to count the number of *G*-edges and vertices in the successively derived graphs to make sure that Lemma 4.8 and Proposition 4.12 are applicable and that the last two factors in (3.11c) come out as claimed. Since every of the $\sigma (\Gamma )\le p$ applications of Proposition 4.12 creates at most 6*p* additional *G*-edges and one additional vertex, it follows that $\left| {{\,\mathrm{GE}\,}}(\Gamma )\right| \le C'p^2$, $\left| V\right| \le C'p$ also in any successively derived graph. Finally, it follows from the last factor in Lemma 4.11 that for each $e\in {{\,\mathrm{IE}\,}}$ with $\deg (e)\ge 5$ we gain additional factors of $N^{-1/2}$. Since $\left| {{\,\mathrm{IE}\,}}\right| \le p$, we easily conclude that if there are more than 4*p* *G*-edges, then each of them comes with an additional gain of $N^{-1/2}$. Now (3.11c) follows immediately after taking the *p*th root.

We turn to the proof of (3.11d). We first write out

$$\begin{aligned} \left\langle {{\,\mathrm{diag}\,}}({\mathbf {p}}{\mathbf {f}}) [T\odot G^t]G\right\rangle = \frac{1}{N} \sum _{a,b} (pf)_a t_{ab} G_{ba} G_{ba} \end{aligned}$$

and therefore can, for even *p*, write the *p*th moment as the value

$$\begin{aligned} {{\,\mathrm{\mathbf {E}}\,}}\left| \left\langle {{\,\mathrm{diag}\,}}({\mathbf {p}}{\mathbf {f}}) [T\odot G^t]G\right\rangle \right| ^p = N^{-p}{{\,\mathrm{Val}\,}}(\Gamma _0) \end{aligned}$$

of the graph $\Gamma _0=(V,{{\,\mathrm{GE}\,}}\cup {{\,\mathrm{IE}\,}})\in \mathcal {G}$ which is given by *p* disjoint 2-cycles as 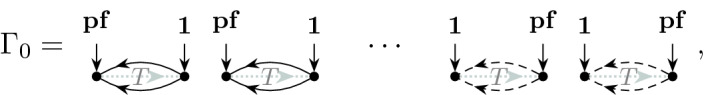


where there are *p*/2 cycles of *G*-edges and *p*/2 cycles of $G^*$ edges. It is clear that $(V,{{\,\mathrm{GE}\,}})$ is 2-degenerate and since $\left| {{\,\mathrm{GE}\,}}\right| =2p$ it follows that

$$\begin{aligned} {{\,\mathrm{W-Est}\,}}(\Gamma _0) \le N^p(\psi +\psi _q')^{2p}. \end{aligned}$$

On the other hand each of the *p* interaction edges in $\Gamma _0$ is a $\sigma$-cell and we can use Proposition 4.12*p* times to obtain (3.11d) just as in the proof of (3.11c). $\square$

### Proof of Proposition 4.12

It follows from the MDE that

$$\begin{aligned} G=M-M\mathcal {S}[M]G- MWG=M-G\mathcal {S}[M]M-GWM, \end{aligned}$$

which we use to locally expand a term of the form $G_{xa}G^*_{ay}$ for fixed *a*, *x*, *y* further. To make the computation local we allow for an arbitrary random function $f=f(W)$, which in practice encodes the remaining *G*-edges in the graph. A simple cumulant expansion shows

4.24 $$\begin{aligned} \sum _{b}B_{ab}{{\,\mathrm{\mathbf {E}}\,}}G_{x b} G^{*}_{b y} f&={{\,\mathrm{\mathbf {E}}\,}}M_{xa} G^*_{ay} f - \sum _{k=2}^{6p} \sum _{b}\sum _{{\varvec{\beta }}\in I^k} \kappa (ba,{\underline{\beta }})m_a {{\,\mathrm{\mathbf {E}}\,}}\partial _{{\varvec{\beta }}}\Big [ G_{xb} G^{*}_{a y} f \Big ] + {\mathcal {O}}\,\left( N^{-p}\right) \nonumber \\&\quad + \sum _b s_{ba} m_a {{\,\mathrm{\mathbf {E}}\,}}\Big [G_{xa}(g-m)_{b} G^*_{ay}+G_{xb} \overline{(g-m)}_a G^*_{by}- G_{xb} G^*_{ay}\partial _{ab} \Big ]f\nonumber \\&\quad +\sum _b t_{ba}m_a {{\,\mathrm{\mathbf {E}}\,}}\Big [G_{xb}(G-M)_{ab} G^*_{ay}+ G_{xb} G^*_{ab}G^*_{ay} - G_{xb} G^*_{ay}\partial _{ba}\Big ]f \nonumber \\ \end{aligned}$$

where $\partial _\alpha :=\partial _{w_\alpha }$ and introduced the stability operator $B:=1-{{\,\mathrm{diag}\,}}(\left| {\mathbf {m}}\right| ^2) S$. The stability operator *B* appears from rearranging the equation obtained from the cumulant expansion to express the quantity ${{\,\mathrm{\mathbf {E}}\,}}G_{x b} G^{*}_{b y} f$. In our graphical representation, the stability operator is a special edge that we can also express as4.25An equality like (4.25) is meant locally in the sense that the pictures only represent subgraphs of the whole graph with the empty, labelled vertices symbolizing those vertices which connect the subgraph to its complement. Thus (4.25) holds true for every fixed graph extending *x*, *y* consistently in all three graphs. The doubly drawn edge in (4.25) means that the external vertices *x*, *y* are identified with each other and the associated indices are set equal via a $\delta _{a_x,a_y}$ function. Thus (4.25) should be understood as the equality4.26where the outside edges incident at the merged vertices *x*, *y* are reconnected to one common vertex in the middle graph. For example, in the picture (4.26) the vertex *x* is connected to the rest of the graph by two edges, and the vertex *y* by one.

In order to represent (4.24) in terms of graphs we have to define a notion of *differential edge*. First, we define a *targeted differential edge* represented by an interaction edge with a red $\partial$-sign written on top and a red-coloured *target* *G*-*edge* to denote the collection of graphs4.27The second picture in (4.27) shows that the target *G*-edge may be a loop; the definition remains the same. This definition extends naturally to $G^*$ edges and is exactly the same for $G-M$ edges (note that this is compatible with the usual notion of derivative as *M* does not depend on *W*). Graphs with the differential signs should be viewed only as an intermediate simplifying picture but they really mean the collection of graphs indicated in the right hand side of (4.27). They represent the identities

$$\begin{aligned} \begin{aligned} \sum _{\alpha } \kappa (uv,\alpha )\partial _{uv} G_{xy}&= - s_{uv} G_{xv} G_{uy} - t_{uv} G_{xu} G_{vy}, \\ \sum _{\alpha } \kappa (uv,\alpha ) \partial _{uv} G_{xx}&= - s_{uv} G_{xv} G_{ux} - t_{uv} G_{xu} G_{vx} \end{aligned} \end{aligned}$$

In other words we introduced these graphs only to temporary encode expressions with derivatives (e.g. second term in the rhs. of (4.24)) *before* the differentiation is actually performed. We can then further define the action of an *untargeted differential edge* according the Leibniz rule as the collection of graphs with the differential edge being targeted on all *G*-edges of the graph one by one (in particular not only those in the displayed subgraph), i.e. for example4.28Here the union is a union in the sense of multisets, i.e. allows for repetitions in the resulting set (note that also this is compatible with the usual action of derivative operations). The $\sqcup \cdots$ symbol on the rhs. of (4.28) indicates that the targeted edge cycles through *all* *G*-edges in the graph, not only the ones in the subgraph. For example, if there are *k* *G*-edges in the graph, then the picture (4.28) represents a collection of 2*k* graphs arising from performing the differentiation

$$\begin{aligned} \begin{aligned}&\sum _\alpha \kappa (uv,\alpha ) \partial _{uv} \big [G_{xy}G_{yz} f\big ]\\&\quad = \sum _\alpha \kappa (uv,\alpha ) \big [\partial _{uv} G_{xy}\big ]G_{yz}f+\sum _\alpha \kappa (uv,\alpha ) G_{xy}\big [\partial _{uv}G_{yz} \big ]f \\&\quad +\sum _\alpha \kappa (uv,\alpha ) G_{xy}G_{yz}\big [\partial _{uv}f \big ] \\&\quad = - s_{uv} \big [G_{xv}G_{uy}G_{yz}f + G_{xy} G_{yv}G_{uz}f + G_{xy} G_{yz} (\partial _{vu}f)\big ]\\&\qquad - t_{uv} \big [G_{xu}G_{vy}G_{yz}f + G_{xy} G_{yu}G_{vz}f+G_{xy} G_{yz} (\partial _{uv}f)\big ], \end{aligned} \end{aligned}$$

where $f=f(W)$ represents the value of the *G*-edges outside the displayed subgraph.

Finally we introduce the notation that a differential edge which is targeted on all *G*-vertices except for those in the displayed subgraph. This differential edge targeted on the outside will be denoted by ${\widehat{\partial }}$.

Regarding the value of the graph, we define the value of a collection of graphs as the sum of their values. We note that this definition is for the collection of graphs encoded by the differential edges also consistent with the usual differentiation.

Written in a graphical form (4.24) reads4.29where the ultimate graph encodes the ultimate terms in the last two lines of (4.24).

We worked out the example for the resolution of the quantity ${{\,\mathrm{\mathbf {E}}\,}}G_{x a} G^{*}_{a y} f$, but very similar formulas hold if the order of the fixed indices (*x*, *y*) and the summation index *a* changes in the resolvents, as well as for other combinations of the complex conjugates. In graphical language this corresponds to changing the arrows of the two *G*-edges adjacent to *a*, as well as their types. In other words, equalities like the one in (4.29) hold true for other any degree two vertex but the stability operator changes slightly: In total there are 16 possibilities, four for whether the two edges are incoming or outgoing at *a* and another four for whether the edges are of type *G* or of type $G^*$. The general form for the stability operator is

4.30 $$\begin{aligned} B:=1-{{\,\mathrm{diag}\,}}({\mathbf {m}}^{\#_1}{\mathbf {m}}^{\#_2})R, \end{aligned}$$

where $R=S$ if there is one incoming and one outgoing edge, $R=T$ if there are two outgoing edges and $R=T^t$ otherwise, and where $\#_1,\#_2$ represent complex conjugations if the corresponding edges are of $G^*$ type. Thus for, for example, the stability operator in *a* for $G_{xa}^*G_{ya}^*$ is $1-{{\,\mathrm{diag}\,}}(\overline{{\mathbf {m}}}^2)T^t$. Note that the stability operator at vertex with degree two is exclusively determined by the type and orientation of the two *G*-edges adjacent to *a*. In the sequel the letter *B* will refer to the appropriate stability operator, we will not distinguish their 9 possibilities ($R=S,T,T^t$ and ${\mathbf {m}}^{\#_1}{\mathbf {m}}^{\#_2}=\left| {\mathbf {m}}\right| ^2,{\mathbf {m}}^2,\overline{{\mathbf {m}}}^2$) in the notation.

#### Lemma 4.13

Let $c>0$ be any constant, $\Gamma \in {\mathcal {G}}$ be a single index graph with at most *cp* vertices and $cp^2$ edges and let $a\in V(\Gamma )$ be a vertex of degree $\deg (a)=2$ not adjacent to a $G$-loop. The insertion of the stability operator *B* (4.30) at *a* as in (4.29) produces a finite set of graphs with at most one additional vertex and 6*p* additional edges, denoted by $\mathcal {G}_\Gamma$, such that

$$\begin{aligned} {{\,\mathrm{Val}\,}}(\Gamma )=\sum _{\Gamma '\in \mathcal {G}_\Gamma } {{\,\mathrm{Val}\,}}\left( \Gamma '\right) +{\mathcal {O}}\,\left( N^{-p}\right) , \end{aligned}$$

and all of them have a Ward estimate

$$\begin{aligned} {{\,\mathrm{W-Est}\,}}(\Gamma ')\le _p \big (\rho +\psi +\eta /\rho +\psi _q'+\psi _q''\big ){{\,\mathrm{W-Est}\,}}(\Gamma )\le _p \sigma _q {{\,\mathrm{W-Est}\,}}(\Gamma ),\qquad \Gamma '\in \mathcal {G}_\Gamma . \end{aligned}$$

Moreover all $\sigma$-cells in $\Gamma$, except possibly a $\sigma$-cell adjacent to *a*, remain $\sigma$-cells also in each $\Gamma '$.

#### Proof

As the proofs for all of the 9 cases of *B*-operators are almost identical we prove the lemma for the case (4.29) for definiteness. Now we compare the value of the graph 
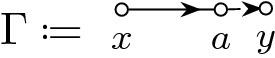


with the graph in the lhs. of (4.29), i.e. when the stability operator *B* is attached to the vertex *a*. We remind the reader that the displayed graphs only show a certain subgraph of the whole graph. The goal is to show that ${{\,\mathrm{W-Est}\,}}\left( \Gamma ' \right) \le \big (\rho +\psi +\eta /\rho +\psi _q'+\psi _q''\big ) {{\,\mathrm{W-Est}\,}}(\Gamma )$ for each graph $\Gamma '$ occurring on the rhs. of (4.29). The forthcoming reasoning is based on comparing the quantities $\left| V\right|$, $\left| {{\,\mathrm{{{\,\mathrm{GE}\,}}_{\text {W}}}\,}}\right|$, $\left| {{\,\mathrm{GE}\,}}_{g-m}\right|$ and $\sum _{e\in {{\,\mathrm{IE}\,}}} \deg (e)/2$ defining the Ward estimate ${{\,\mathrm{W-Est}\,}}$ from (4.19b) of the graph $\Gamma$ and the various graphs $\Gamma '$ occurring on the rhs. of (4.29).

- We begin with the first graph and claim that 
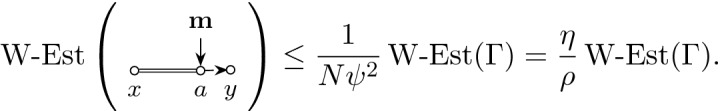
 Due to the double edge which identifies the *x* and *a* vertices it follows that $\left| V(\Gamma ')\right| =\left| V(\Gamma )\right| -1$. The degrees of all interaction edges remain unchanged when going from $\Gamma$ to $\Gamma '$. As the 2-degenerate set of Wardable edges ${{\,\mathrm{{{\,\mathrm{GE}\,}}_{\text {W}}}\,}}(\Gamma ')$ we choose ${{\,\mathrm{{{\,\mathrm{GE}\,}}_{\text {W}}}\,}}(\Gamma ){\setminus } N(a)$, i.e. the 2-degenerate edge set in the original graph except for the edge-neighbourhood *N*(*a*) of *a*, i.e. those edges adjacent to *a*. As a subgraph of $(V,{{\,\mathrm{{{\,\mathrm{GE}\,}}_{\text {W}}}\,}}(\Gamma ))$ it follows that $(V{\setminus }\{a\},{{\,\mathrm{{{\,\mathrm{GE}\,}}_{\text {W}}}\,}}(\Gamma '))$ is again 2-degenerate. Thus $\left| {{\,\mathrm{{{\,\mathrm{GE}\,}}_{\text {W}}}\,}}(\Gamma )\right| \ge \left| {{\,\mathrm{{{\,\mathrm{GE}\,}}_{\text {W}}}\,}}(\Gamma ')\right| \ge \left| {{\,\mathrm{{{\,\mathrm{GE}\,}}_{\text {W}}}\,}}(\Gamma )\right| -2$ and the claimed bound follows since $\left| {{\,\mathrm{GE}\,}}_{g-m}(\Gamma ')\right| =\left| {{\,\mathrm{GE}\,}}_{g-m}(\Gamma )\right|$ and
  $$\begin{aligned} \frac{{{\,\mathrm{W-Est}\,}}(\Gamma ')}{{{\,\mathrm{W-Est}\,}}(\Gamma )} = \frac{1 }{N (\psi +\psi _q')^{\left| {{\,\mathrm{{{\,\mathrm{GE}\,}}_{\text {W}}}\,}}(\Gamma )\right| -\left| {{\,\mathrm{{{\,\mathrm{GE}\,}}_{\text {W}}}\,}}(\Gamma ')\right| } }\le \frac{1}{N\psi ^2}. \end{aligned}$$
- Next, we consider the third and fourth graph and claim that 
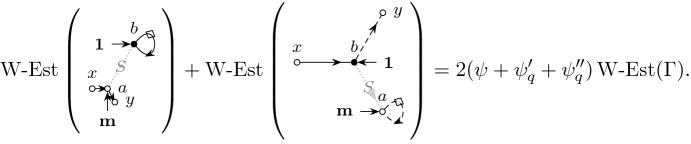
 Here there is one more vertex (corresponding to an additional summation index), $\left| V(\Gamma ')\right| =\left| V(\Gamma )\right| +1$, whose effect in (4.19b) is compensated by one additional interaction edge *e* of degree 2. Hence the *N*-exponent $n(\Gamma )$ remains unchanged. In the first graph we can simply choose ${{\,\mathrm{{{\,\mathrm{GE}\,}}_{\text {W}}}\,}}(\Gamma ')={{\,\mathrm{{{\,\mathrm{GE}\,}}_{\text {W}}}\,}}(\Gamma )$, whereas in the second graph we choose ${{\,\mathrm{{{\,\mathrm{GE}\,}}_{\text {W}}}\,}}(\Gamma ')={{\,\mathrm{{{\,\mathrm{GE}\,}}_{\text {W}}}\,}}(\Gamma ){\setminus }\{(x,a),(a,y)\}\cup \{(x,b),(b,y)\}$ which is 2-degenerate as a subgraph of a 2-degenerate graph together with an additional vertex of degree 2. Thus in both cases we can choose ${{\,\mathrm{{{\,\mathrm{GE}\,}}_{\text {W}}}\,}}(\Gamma ')$ (if necessary, by removing excess edges from ${{\,\mathrm{{{\,\mathrm{GE}\,}}_{\text {W}}}\,}}(\Gamma ')$ again) in such a way that $\left| {{\,\mathrm{{{\,\mathrm{GE}\,}}_{\text {W}}}\,}}(\Gamma ')\right| =\left| {{\,\mathrm{{{\,\mathrm{GE}\,}}_{\text {W}}}\,}}(\Gamma )\right|$ but the number of $(g-m)$-loops is increased by 1, i.e. $\left| {{\,\mathrm{GE}\,}}_{g-m}(\Gamma ')\right| =\left| {{\,\mathrm{GE}\,}}_{g-m}(\Gamma )\right| +1$.
- Similarly, we claim for the fifth and sixth graph that 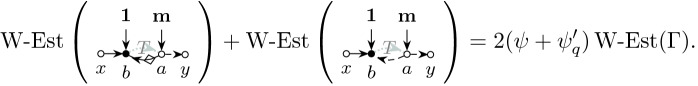 There is one more vertex whose effect in (4.19b) is compensated by one more interaction edge of degree 2, whence the number *N*-exponent remains unchanged. The number of Wardable edges can be increased by one by setting ${{\,\mathrm{{{\,\mathrm{GE}\,}}_{\text {W}}}\,}}(\Gamma ')$ to be a suitable subset of ${{\,\mathrm{{{\,\mathrm{GE}\,}}_{\text {W}}}\,}}(\Gamma ){\setminus }\{(x,a),(a,y)\}\cup \{(x,b),(a,b),(a,y)\}$ which is 2-degenerate as the subset of a 2-degenerate graph together with two vertices of degree 2. The number of $(g-m)$-loops remains unchanged.
- For the last graph in (4.29), i.e. where the derivative targets an outside edge, we claim that 
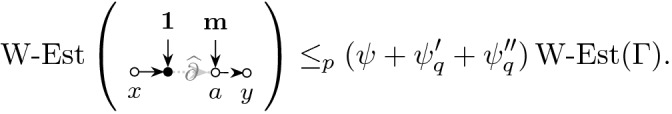
 Here the argument on the lhs., $\Gamma '$, stands for a whole collection of graphs but we essentially only have to consider two types: The derivative edge either hits a *G*-edge or a $(g-m)$-loop, i.e. 
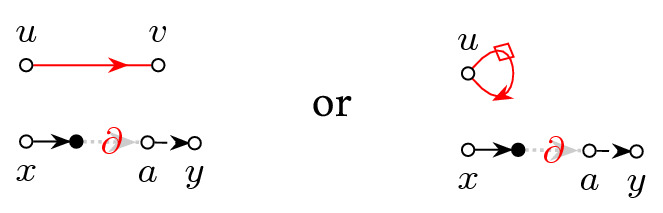
 which encodes the graphs 
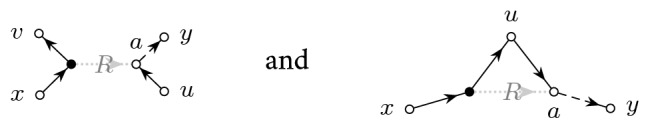
 as well as the corresponding transpositions (as in (4.27)). In both cases the *N*-size of ${{\,\mathrm{W-Est}\,}}$ remains constant since the additional vertex is balanced by the additional degree two interaction edge. In both cases all four displayed edges can be included in ${{\,\mathrm{{{\,\mathrm{GE}\,}}_{\text {W}}}\,}}(\Gamma ')$. So $\left| {{\,\mathrm{{{\,\mathrm{GE}\,}}_{\text {W}}}\,}}\right|$ can be increased by 1 in the first case and by 2 in the second case while the number of $(g-m)$-loops remains constant in the first case is decreased by 1 in the second case. The claim follows directly in the first case and from
  $$\begin{aligned} \frac{{{\,\mathrm{W-Est}\,}}(\Gamma ')}{{{\,\mathrm{W-Est}\,}}(\Gamma )}=\frac{(\psi +\psi '_q)^2}{\psi +\psi '_q+\psi ''_q} \le \psi +\psi '_q +\psi ''_q \end{aligned}$$
  in the second case.
- It remains to consider the second graph in the rhs. of (4.29) with the higher derivative edge. We claim that for each $k\ge 2$ it holds that 
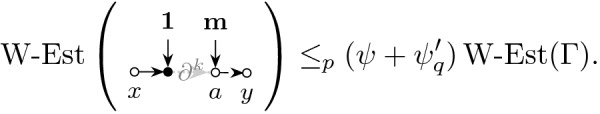
 We prove the claim by induction on *k* starting from $k=2$. For any $k\ge 2$ we write $\partial ^k = \partial ^{k-1}\partial$. For the action of the last derivative we distinguish three cases: (i) action on an edge adjacent to the derivative edge, (ii) action on a non-adjacent *G*-edge and (iii) an action on a non-adjacent $(g-m)$-loop. Graphically this means 4.31 We ignored the case where the derivative acts on (*a*, *y*) since it is estimated identically to the first graph. We also neglected the possibility that the derivative acts on a *g*-loop, as this is estimated exactly as the last graph and the result is even better since no $(g-m)$-loop is destroyed. After performing the last derivative in (4.31) we obtain the following graphs $\Gamma '$4.32 where we neglected the transposition of the third graph with *u*, *v* exchanged because this is equivalent with regard to the counting argument. First, we handle the second, third and fourth graphs in (4.32). In all these cases the set ${{\,\mathrm{{{\,\mathrm{GE}\,}}_{\text {W}}}\,}}(\Gamma ')$ is defined simply by adding all edges drawn in (4.32) to the set ${{\,\mathrm{{{\,\mathrm{GE}\,}}_{\text {W}}}\,}}(\Gamma ){\setminus } \{(x,a), (a,y)\}$. The new set remains 2-degenerate since all these new edges are adjacent to vertices of degree 2. Compared to the original graph, $\Gamma$, we thus have increased $\left| {{\,\mathrm{{{\,\mathrm{GE}\,}}_{\text {W}}}\,}}\right| +\left| {{\,\mathrm{GE}\,}}_{g-m}\right|$ by at least 1.
  We now continue with the first graph in (4.32), where we explicitly expand the action of another derivative (notice that this is the only graph where $k\ge 2$ is essentially used). We distinguish four cases, depending on whether the derivative acts on (i) the *b*-loop, (ii) an adjacent edge, (iii) a non-adjacent edge or (iv) a non-adjacent $(g-m)$-loop, i.e. graphically we have 4.33 After performing the indicated derivative, the encoded graphs $\Gamma '$ are 4.34 where we again neglected the version of the third graph with *u*, *v* exchanged. We note that both the first and the second graph in (4.33) produce the first graph in (4.34). Now we define how to get the set ${{\,\mathrm{{{\,\mathrm{GE}\,}}_{\text {W}}}\,}}(\Gamma ')$ from ${{\,\mathrm{{{\,\mathrm{GE}\,}}_{\text {W}}}\,}}(\Gamma ){\setminus }\{ (x,a), (a,y)\}$ for each case. In the first graph of (4.34) we add all three non-loop edges to ${{\,\mathrm{{{\,\mathrm{GE}\,}}_{\text {W}}}\,}}(\Gamma ')$, in the second graph we add both non-loop edges, in the third and fourth graph we add the non-looped edge adjacent to *b* as well as any two non-looped edges adjacent to *a*. Thus, compared to the original graph the number $\left| {{\,\mathrm{{{\,\mathrm{GE}\,}}_{\text {W}}}\,}}\right| +\left| {{\,\mathrm{GE}\,}}_{g-m}\right|$ is at least preserved. On the other hand the *N*-power counting is improved by $N^{-1/2}$. Indeed, there is one additional vertex *b*, yielding a factor *N*, which is compensated by the scaling factor $N^{-3/2}$ from the interaction edge of degree 3.
     To conclude the inductive step we note that additional derivatives (i.e. the action of $\partial ^{k-2}$) can only decrease the Ward-value of a graph. Indeed, any single derivative can at most decrease the number $\left| {{\,\mathrm{{{\,\mathrm{GE}\,}}_{\text {W}}}\,}}(\Gamma )\right| +\left| {{\,\mathrm{GE}\,}}_{g-m}\right|$ by 1 by either differentiating a $(g-m)$-loop or differentiating an edge from ${{\,\mathrm{{{\,\mathrm{GE}\,}}_{\text {W}}}\,}}$. Thus the number $\left| {{\,\mathrm{{{\,\mathrm{GE}\,}}_{\text {W}}}\,}}\right| +\left| {{\,\mathrm{GE}\,}}_{g-m}\right|$ is decreased by at most $k-2$ while the number $\left| {{\,\mathrm{GE}\,}}_{g-m}\right|$ is not increased. In particular, by choosing a suitable subset of Wardable edges, we can define ${{\,\mathrm{{{\,\mathrm{GE}\,}}_{\text {W}}}\,}}(\Gamma ')$ in such a way that $\left| {{\,\mathrm{{{\,\mathrm{GE}\,}}_{\text {W}}}\,}}\right| +\left| {{\,\mathrm{GE}\,}}_{g-m}\right|$ is decreased by exactly $k-2$. But at the same time each derivative provides a gain of $cN^{-1/2}\le \psi \le \psi +\psi _q'$ since the degree of the interaction edge is increased by one. Thus we have
  $$\begin{aligned} \frac{{{\,\mathrm{W-Est}\,}}(\Gamma ')}{{{\,\mathrm{W-Est}\,}}(\Gamma )} \le _p (\psi +\psi _q')^{k-1 +\left| {{\,\mathrm{{{\,\mathrm{GE}\,}}_{\text {W}}}\,}}(\Gamma ')\right| +\left| {{\,\mathrm{GE}\,}}_{g-m}(\Gamma ')\right| -\left| {{\,\mathrm{{{\,\mathrm{GE}\,}}_{\text {W}}}\,}}(\Gamma )\right| -\left| {{\,\mathrm{GE}\,}}_{g-m}(\Gamma )\right| } = \psi +\psi _q', \end{aligned}$$
  just as claimed.$\square$

Lemma 4.13 shows that the insertion of the *B*-operator reduces the Ward-estimate by at least $\rho$. However, this insertion does not come for free since the inverse

$$\begin{aligned} B^{-1} = (1-{{\,\mathrm{diag}\,}}({\mathbf {m}}^{\#_1}{\mathbf {m}}^{\#_2})R)^{-1} \end{aligned}$$

is generally not a uniformly bounded operator. For example, it follows from (2.2) that

$$\begin{aligned} \mathfrak {I}{\mathbf {m}}= \eta \left| {\mathbf {m}}\right| ^2 + \left| {\mathbf {m}}\right| ^2 S\mathfrak {I}{\mathbf {m}}\end{aligned}$$

and therefore $(1-{{\,\mathrm{diag}\,}}(\left| {\mathbf {m}}\right| ^2) S)^{-1}$ is singular for small $\eta$ with $\mathfrak {I}{\mathbf {m}}$ being the unstable direction. It turns out, however, that *B* is invertible on the subspace complementary to some bad direction ${\mathbf {b}}^{(B)}$. At this point we distinguish two cases. If *B* has a uniformly bounded inverse, i.e. if $\Vert B^{-1}\Vert _{\infty \rightarrow \infty }\le C$ for some constant $C>0$, then we set $P_B:=0$. Otherwise we define $P_B$ as the spectral projection operator onto the eigenvector ${\mathbf {b}}^{(B)}$ of *B* corresponding to the eigenvalue $\beta$ with smallest modulus:

4.35 $$\begin{aligned} P_B:=\frac{\left\langle {\mathbf {l}}^{(B)},\cdot \right\rangle }{\left\langle {\mathbf {l}}^{(B)},{\mathbf {b}}^{(B)}\right\rangle } {\mathbf {b}}^{(B)},\qquad Q_B:=1-P_B, \end{aligned}$$

where $\left\langle \mathbf {v},{\mathbf {w}}\right\rangle :=N^{-1} \sum _a \overline{v_a} w_a$ denotes the normalized inner product and ${\mathbf {l}}^{(B)}$ is the corresponding left eigenvector, $(B^*-\beta ){\mathbf {l}}^{(B)} = 0$.

#### Lemma 4.14

For all 9 possible *B*-operators in (4.30) it holds that

4.36 $$\begin{aligned} \Vert B^{-1} Q_B\Vert _{\infty \rightarrow \infty } \le C < \infty \end{aligned}$$

for some constant $C>0$, depending only on model parameters.

#### Proof

First we remark that it is sufficient to prove the bound (4.36) on $B^{-1}Q_B$ as an operator on $\mathbb {C}^N$ with the Euclidean norm, i.e. $\Vert B^{-1} Q_B\Vert \le C$. For this insight we refer to [5, Proof of (5.28) and (5.40a)]. Recall that $R =S$, $R=T$ or $R=T^t$, depending on which stability operator we consider (cf. (4.30)). We begin by considering the complex hermitian symmetry class and the cases $R=T$ and $R=T^t$. We will now see that in this case *B* has a bounded inverse and thus $Q_B =1$. Indeed, we have

$$\begin{aligned} \Vert B^{-1}\Vert \lesssim \frac{1}{1-\Vert F^{(R)}\Vert }, \end{aligned}$$

where $F^{(R)}{\mathbf {w}}:=\left| {\mathbf {m}}\right| R (\left| {\mathbf {m}}\right| {\mathbf {w}})$. The fullness Assumption (B) in (2.3) implies that $\left| t_{ij}\right| \le (1-c)s_{ij}$ for some constant $c>0$ and thus $\Vert F^{(R)}\Vert \le (1-c)\Vert F^{(S)}\Vert \le 1-c$ for $R=T,T^t$. Here we used $\Vert F^{(S)}\Vert \le 1$, a general property of the saturated self-energy matrix $F^{(S)}$ that was first established in [6, Lemma 4.3] (see also [7, Eq. (4.24)] and [10, Eq. (4.5)]). Now we turn to the case $R=S$ for both the real symmetric and complex hermitian symmetry classes. In this case *B* is the restriction to diagonal matrices of an operator ${\mathcal {T}}: \mathbb {C}^{N \times N} \rightarrow \mathbb {C}^{N \times N}$, where ${\mathcal {T}} \in \{\mathrm {Id}-M^*{\mathcal {S}}[\cdot ]M,\mathrm {Id}-M{\mathcal {S}}[\cdot ]M,\mathrm {Id}-M^*{\mathcal {S}}[\cdot ]M^*\}$. All of these operators were covered in [10, Lemma 5.1] and thus (4.36) is a consequence of that lemma. Recall that the flatness (3.6) of ${\mathcal {S}}$ ensured the applicability of the lemma. $\square$

We will insert the identity $1= P_B+BB^{-1}Q_B$, and we will perform an explicit calculation for the $P_B$ component, while using the boundedness of $B^{-1}Q_B$ in the other component. We are thus left with studying the effect of inserting *B*-operators and suitable projections into a $\sigma$-cell. To include all possible cases with regard to edge-direction and edge-type (i.e. *G* or $G^*$), in the pictures below we neither indicate directions of the *G*-edges nor their type but implicitly allow all possible assignments. We recall that both the *R*-interaction edge as well as the *relevant* *B*-operators (cf. (4.30)) are completely determined by the type of the four *G*-edges as well as their directions. To record the type of the inserted *B*, $P_B$, $Q_B$ operators we call those inserted on the rhs. of the *R*-edge $B'$, $P_B'$ and $Q_B'$ in the following graphical representations. Pictorially we first decompose the $\sigma$-cell subgraph of some graph $\Gamma$ as4.37where we allow the vertices *x*, *y* to agree with *z* or *w*. With formulas, the insertion in (4.37) means the following identity

$$\begin{aligned} \sum _{ab} (pf)_a G_{ya} G_{xa} R_{ab} G_{bw} G_{bz} = \sum _{abc} (pf)_c G_{ya} G_{xa} \big (P_{ac} + Q_{ac} \big ) R_{cb} G_{bw} G_{bz} \end{aligned}$$

since $P_{ac} + Q_{ac} =\delta _{ac}$. We first consider with the second graph in (4.37), whose treatment is independent of the specific weights, so we already removed the weight information. We insert the *B* operator as 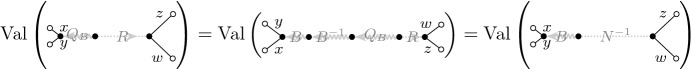


and notice that due to Lemma 4.14 the matrix $K=(B^{-1})^t Q_B^t R$, assigned to the weighted edge in the last graph, is entry-wise $\left| k_{ab}\right| \le cN^{-1}$ bounded (the transpositions compensate for the opposite orientation of the participating edges). It follows from Lemma 4.13 that4.38where all $\Gamma '\in \mathcal {G}_\Gamma$ satisfy ${{\,\mathrm{W-Est}\,}}(\Gamma ')\le _p \sigma _q {{\,\mathrm{W-Est}\,}}(\Gamma )$ and all $\sigma$-cells in $\Gamma$ except for the currently expanded one remain $\sigma$-cells in $\Gamma '$. We note that it is legitimate to compare the Ward estimate of $\Gamma '$ with that of $\Gamma$ because with respect to the Ward-estimate there is no difference between $\Gamma$ and the modification of $\Gamma$ in which the *R*-edge is replaced by a generic $N^{-1}$-weighted edge.

We now consider the first graph in (4.37) and repeat the process of inserting projections $P_B'+Q_B'$ to the other side of the *R*-edge to find4.39where we already neglected those weights which are of no importance to the bound. The argument for the second graph in (4.39) is identical to the one we used in (4.38) and we find another finite collection of graphs $\mathcal {G}'_\Gamma$ such that4.40where the weighted edge carries the weight matrix $K=P_{B}^t R Q_{B'} B'^{-1}$, which is according to Lemma 4.14 indeed scales like $\left| k_{ab}\right| \le cN^{-1}$. The graphs $\Gamma '\in \mathcal {G}_\Gamma '$ also satisfy ${{\,\mathrm{W-Est}\,}}(\Gamma ')\le _p \sigma _q {{\,\mathrm{W-Est}\,}}(\Gamma )$ and all $\sigma$-cells in $\Gamma$ except for the currently expanded one remain $\sigma$-cells in $\Gamma '$.

It remains to consider the first graph in (4.39) in the situation where *B* does not have a bounded inverse. We compute the weight matrix of the $P_B^t R P_B'$ interaction edge as

$$\begin{aligned} \begin{aligned} P_B^t {{\,\mathrm{diag}\,}}({\mathbf {p}}{\mathbf {f}}) R P_B'&= \left( \frac{\left\langle \overline{{\mathbf {b}}^{(B)}},\cdot \right\rangle }{\left\langle \overline{{\mathbf {b}}^{(B)}},\overline{{\mathbf {l}}^{(B)}}\right\rangle }\overline{{\mathbf {l}}^{(B)}}\right) \left[ {{\,\mathrm{diag}\,}}({\mathbf {p}}{\mathbf {f}}) R \frac{\left\langle {\mathbf {l}}^{(B')},\cdot \right\rangle }{\left\langle {\mathbf {l}}^{(B')},{\mathbf {b}}^{(B')}\right\rangle }{\mathbf {b}}^{(B')} \right] \\&= \frac{\left\langle {\mathbf {b}}^{(B)}{\mathbf {p}}{\mathbf {f}}(R {\mathbf {b}}^{(B')}) \right\rangle }{\left\langle \overline{{\mathbf {b}}^{(B)}},\overline{{\mathbf {l}}^{(B)}}\right\rangle }\frac{\left\langle {\mathbf {l}}^{(B')},\cdot \right\rangle \overline{{\mathbf {l}}^{(B)}}}{\left\langle {\mathbf {l}}^{(B')},{\mathbf {b}}^{(B')}\right\rangle } \end{aligned} \end{aligned}$$

which we separate into the scalar factor

$$\begin{aligned} \frac{\left\langle {\mathbf {b}}^{(B)}{\mathbf {p}}{\mathbf {f}}(R {\mathbf {b}}^{(B')}) \right\rangle \left\langle {\mathbf {l}}^{(B')},\overline{{\mathbf {l}}^{(B)}}\right\rangle }{\left\langle \overline{{\mathbf {b}}^{(B)}},\overline{{\mathbf {l}}^{(B)}}\right\rangle \left\langle {\mathbf {l}}^{(B')},{\mathbf {b}}^{(B')}\right\rangle } \end{aligned}$$

and the weighted edge

4.41 $$\begin{aligned} K=\frac{\left\langle {\mathbf {l}}^{(B')},\cdot \right\rangle \overline{{\mathbf {l}}^{(B)}}}{\left\langle {\mathbf {l}}^{(B')},\overline{{\mathbf {l}}^{(B)}}\right\rangle } \end{aligned}$$

which scales like $\left| k_{ab}\right| \le cN^{-1}$ since ${\mathbf {l}}$ is $\ell ^2$-normalised and delocalised. Thus we can write4.42Note that the *B* and $B'$ operators are not completely independent: According to Fact 1 it follows that for an interaction edge $e=(u,v)$ associated with the matrix *R* the number of incoming *G*-edges in *u* is the same as the number of outgoing *G*-edges from *v*, and vice versa. Thus, according to (4.30), the *B*-operator at *u* comes with an *S* if and only if the $B'$-operator at *v* comes also with an *S*. Furthermore, if the *B*-operator comes with an *T*, then the $B'$-operator comes with an $T^t$, and vice versa. The distribution of the conjugation operators to $B,B'$ in (4.30), however, can be arbitrary. We now use the fact that the scalar factor in (4.42) can be estimated by $\left| \sigma \right| +\rho +\eta /\rho$ (cf. Lemma A.2). Summarising the above arguments, from (4.37)–(4.42), the proof of Proposition 4.12 is complete.

## Cusp Universality

The goal of this section is the proof of cusp universality in the sense of Theorem 2.3. Let *H* be the original Wigner-type random matrix with expectation $A:={{\,\mathrm{\mathbf {E}}\,}}H$ and variance matrix $S=(s_{ij})$ with $s_{ij}:={{\,\mathrm{\mathbf {E}}\,}}\left| h_{ij}-a_{ij}\right| ^2$ and $T=(t_{ij})$ with $t_{ij}:={{\,\mathrm{\mathbf {E}}\,}}(h_{ij}-a_{ij})^2$. We consider the Ornstein Uhlenbeck process $\{{\widetilde{H}}_t| t\ge 0\}$ starting from $\widetilde{H}_0=H$, i.e.

5.1 $$\begin{aligned} \mathrm{d}\widetilde{H}_t = - \frac{1}{2}(\widetilde{H}_t-A)\mathrm{d}t+\Sigma ^{1/2}[\mathrm{d}B_t], \qquad \Sigma [R]:={{\,\mathrm{\mathbf {E}}\,}}W{{\,\mathrm{Tr}\,}}(W R) \end{aligned}$$

which preserves expectation and variance. In our setting of deformed Wigner-type matrices the covariance operator $\Sigma : \mathbb {C}^{N \times N} \rightarrow \mathbb {C}^{N \times N}$ is given by

$$\begin{aligned} \Sigma [R] :=S \odot R+ T\odot R^t. \end{aligned}$$

The OU process effectively adds a small Gaussian component to $$\widetilde{H}_t$$ along the flow in the sense that $${\tilde{H}}_t=A+e^{-t/2} (H-A) + \widetilde{U}_t$$ in distribution with $$\widetilde{U}_t$$ being and independent centred Gaussian matrix with covariance $$\mathbf {Cov}(\widetilde{U})= (1-e^{-t/2})\Sigma $$. Due to the fullness Assumption (B) there exist small $$c,t_*$$ such that $$\widetilde{U}_t$$ can be decomposed as $$\widetilde{U}_t=\sqrt{ct} U+U'_t$$ with $$U\sim \mathrm {GUE}$$ and $$U_t'$$ Gaussian and independent of *U* for $$t\le t_*$$. Thus there exists a Wigner-type matrix $$ H_t$$ such that

5.2 $$\begin{aligned} \begin{aligned} \widetilde{H}_t&= H_t + \sqrt{ct} U, \qquad \mathcal {S}_t= \mathcal {S}- ct\mathcal {S}^{\mathrm {GUE}}, \qquad {{\,\mathrm{\mathbf {E}}\,}}H_t = A, \\ U&\sim \text {GUE}, \qquad \mathcal {S}^{\mathrm {GUE}}[R] :=\left\langle R\right\rangle = \frac{1}{N}{{\,\mathrm{Tr}\,}}R \end{aligned} \end{aligned}$$

with *U* independent of $$ H_t$$. Note that we do not define $$H_t$$ as a stochastic process and we will use the representation (5.2) only for one carefully chosen $$t=N^{-1/2+\epsilon }$$. We note that $$H_t$$ satisfies the assumption of our local law from Theorem 2.5. It thus follows that $$G_t:=(H_t-z)^{-1}$$ is well approximated by the solution $$M_t={{\,\mathrm{diag}\,}}(M_t)$$ to the MDE

$$\begin{aligned} -M_t^{-1} = z-A + \mathcal {S}_t[M_t]. \qquad \rho _t(E):=\lim _{\eta \searrow 0}\frac{\mathfrak {I}\left\langle M_t(E+i\eta )\right\rangle }{\pi }. \end{aligned}$$

In particular, by setting $t=0$, $M_0$ well approximates the resolvent of the original matrix *H* and $\rho _0=\rho$ is its self-consistent density. Note that the Dyson equation of $\widetilde{H}_t$ and hence its solution as well are independent of *t*, since they are entirely determined by the first and second moments of $\widetilde{H}_t$ that are the same *A* and *S* for any *t*. Thus the resolvent of $\widetilde{H}_t$ is well approximated by the same $M_{0}$ and the self-consistent density of $\widetilde{H}_t$ is given by $\rho _0=\rho$ for any *t*. While *H* and $\widetilde{H}_t$ have identical self-consistent data, structurally they differ in a key point: $\widetilde{H}_t$ has a small Gaussian component. Thus the correlation kernel of the local eigenvalue statistics has a contour integral representation using a version of the Brézin–Hikami formulas, see Sect. 5.2.

The contour integration analysis requires a Gaussian component of size at least $ct\gg N^{-1/2}$ and a very precise description of the eigenvalues of $H_t$ just above the scale of the eigenvalue spacing. This information will come from the optimal rigidity, Corollary 2.6, and the precise shape of the self-consistent density of states of $H_t$. The latter will be analysed in Sect. 5.1 where we describe the evolution of the density near the cusp under an additive GUE perturbation $\sqrt{s} U$. We need to construct $H_t$ with a small gap carefully so that after a relatively long time $s=ct$ the matrix $H_t+ \sqrt{ct} U$ develops a cusp exactly at the right location. In fact, we the process has two scales in the shifted variable $\nu = s-ct$ that indicates the time relative to the cusp formation. It turns out that the *locations* of the edges typically move linearly with $\nu$, while the *length of the gap* itself scales like $(-\nu )_+^{3/2}$, i.e. it varies much slower and we need to fine-tune the evolution of both.

To understand this tuning process, we fix $t= N^{-1/2+\epsilon }$ and we consider the matrix flow $s\rightarrow H_t(s):=H_t + \sqrt{s}U$ for any $s\ge 0$ and not just for $s=ct$. It is well known that the corresponding self-consistent densities are given by the semicircular flow. Equivalently, these densities can be described by the free convolution of $\rho _t$ with a scaled semicircular distribution $\rho _{\text {sc}}$. In short, the self-consistent density of $H_t(s)$ is given by $\rho ^\text {fc}_s :=\rho _t \boxplus \sqrt{s} \rho _{\text {sc}}$, where we omitted *t* from the notation $\rho ^\text {fc}_s$ since we consider *t* fixed. In particular we have $\rho ^\text {fc}_0=\rho _t$, the density of $H_t$ and $\rho ^\text {fc}_{ct}=\rho$, the density of $\widetilde{H}_t= H_t+\sqrt{ct} U$ as well as that of *H*. Hence, as a preparation to the contour integration, in Sect. 5.1 we need to describe the cusp formation along the semicircular flow. Before going into details, we describe the strategy.

Since in the sequel the densities $\rho ^\text {fc}_s$ and their local minima and gaps will play an important role, we introduce the convention that properties of the original density $\rho$ will always carry $\rho$ as a superscript for the remainder of Sect. 5. In particular, the points $\mathfrak {c},\mathfrak {e}_\pm ,\mathfrak {m}$ and the gap size $\Delta$ from (2.4) and Theorem 2.3 will from now on be denoted by $\mathfrak {c}^\rho ,\mathfrak {e}_\pm ^\rho , \mathfrak {m}^\rho$ and $\Delta ^\rho$. In particular a superscript of $\rho$ never denotes a power.

### Proof strategy

First we consider case (i) when $\rho$, the self-consistent density associated with *H*, has an exact cusp at the point $\mathfrak {c}^\rho \in \mathbb {R}$. Note that $\mathfrak {c}^\rho$ is also a cusp point of the self-consistent density of $\widetilde{H}_t$ for any *t*.

We set $t:=N^{-1/2+\epsilon }$. Define the functions

$$\begin{aligned} \Delta (\nu ) :=(2\gamma )^2 (\nu /3)^{3/2}\qquad \text {and}\qquad \rho ^{\min }(\nu ):=\gamma ^2\sqrt{\nu }/\pi \end{aligned}$$

for any $\nu \ge 0$. For $s<ct$ denote the gap in the support of $\rho ^\text {fc}_s$ close to $\mathfrak {c}^\rho$ by $[\mathfrak {e}_s^-,\mathfrak {e}_s^+]$ and its length by $\Delta _s:=\mathfrak {e}_s^+-\mathfrak {e}_s^-$. In Sect. 5.1 we will prove that if $\rho$ has an exact cusp in $\mathfrak {c}^\rho$ as in (2.4a), then $\rho ^\text {fc}_s$ has a gap of size $\Delta _s\approx \Delta (ct-s)$, and, in particular, $\rho _t=\rho ^\text {fc}_0$ has a gap of size $\Delta _0\approx \Delta (ct)\sim t^{3/2}$, only depending on *c*, *t* and $\gamma$. The distance of $\mathfrak {c}^\rho$ from the gap is $\approx \text {const}\cdot t$. This overall shift will be relatively easy to handle, but notice that it must be tracked very precisely since the gap changes much slower than its location. For $s>ct$ with $s-ct={\mathcal {O}}\,\left( 1\right)$ we will similarly prove that $\rho ^\text {fc}_s$ has no gap anymore close to $\mathfrak {c}^\rho$ but a unique local minimum in $\mathfrak {m}_s$ of size $\rho ^\text {fc}_s(\mathfrak {m}_s)\approx \rho ^{\min }(s-ct)$.

Now we consider the case where $\rho$ has no exact cusp but a small gap of size $\Delta ^\rho >0$. We parametrize this gap length via a parameter $t^\rho >0$ defined by $\Delta ^\rho =\Delta (t^\rho )$. It follows from the associativity (5.3b) of the free convolution that $\rho _t$ has a gap of size $\Delta _0\approx \Delta (ct+t^\rho )$.

Finally, the third case is where $\rho$ has a local minimum of size $\rho (\mathfrak {m}^\rho )$. We parametrize it as $\rho (\mathfrak {m}^\rho )=\rho ^{\min }(t^\rho )$ with $0<t^\rho <ct$ then it follows that $\rho _t$ has a gap of size $\Delta _0\approx \Delta (ct-t^\rho )$.

Note that these conclusions follow purely from the considerations in Sect. 5.1 for exact cusps and the associativity of the free convolution. We note that in both almost cusp cases $t^\rho$ should be interpreted as a time (or reverse time) to the cusp formation.

In the final part of the proof in Sects. 5.2–5.3 we will write the correlation kernel of $H_t+\sqrt{ct} U$ as a contour integral purely in terms of the mesoscopic shape parameter $\gamma$ and the gap size $\Delta _0$ of the density $\rho _t$ associated with $H_t$. If $\Delta _0\approx \Delta (ct)$, then the gap closes after time $s\approx ct$ and we obtain a Pearcey kernel with parameter $\alpha =0$. If $\Delta _0\approx \Delta (ct+t^\rho )$ and $t^\rho \sim N^{-1/2}$, then the gap does not quite close at time $s=ct$ and we obtain a Pearcey kernel with $\alpha >0$, while for $\Delta _0\approx \Delta (ct-t^\rho )$ with $t^\rho \sim N^{-1/2}$ the gap after time $s=ct$ is transformed into a tiny local minimum and we obtain a Pearcey kernel with $\alpha <0$. The precise value of $\alpha$ in terms of $\Delta ^\rho$ and $\rho (\mathfrak {m}^\rho )$ are given in (2.6). Note that as an input to the contour integral analysis, in all three cases we use the local law only for $H_t$, i.e. in a situation when there is a small gap in the support of $\rho _t$, given by $\Delta _0$ defined as above in each case.

### Free convolution near the cusp

In this section we quantitatively investigate the free semi-circular flow before and after the formation of cusp. We first establish the exact rate at which a gap closes to form a cusp, and the rate at which the cusp is transformed into a non-zero local minimum. We now suppose that $\rho ^*$ is a general density with a small spectral gap $[\mathfrak {e}_-^*,\mathfrak {e}_+^*]$ whose Stieltjes transform $m^*$ can be obtained from solving a Dyson equation. Let $\rho _{\mathrm {sc}}(x):=\sqrt{(4-x^2)_+}/2\pi$ be the density of the semicircular distribution and let $s\ge 0$ be a time parameter. The free semicircular convolution $\rho _s^\text {fc}$ of $\rho ^*$ with $\sqrt{s}\rho _\mathrm {sc}$ is then defined implicitly via its Stieltjes transform

5.3a $$\begin{aligned} m_s^\text {fc}(z) = m^*(\xi _s(z))= m^*(z+ s m_s^\text {fc}(z)),\qquad \xi _s(z):=z + s m_s^\text {fc}(z), \qquad z,m_s^\text {fc}(z)\in \mathbb {H}.\nonumber \\ \end{aligned}$$

It follows directly from the definition that $s\mapsto m_s^\text {fc}$ is *associative* in the sense that

5.3b $$\begin{aligned} m_{s+s'}^\text {fc}(z) = m_s(z+ s' m_{s+s'}^\text {fc}(z)), \qquad s,s'\ge 0. \end{aligned}$$

**Fig. 1:**
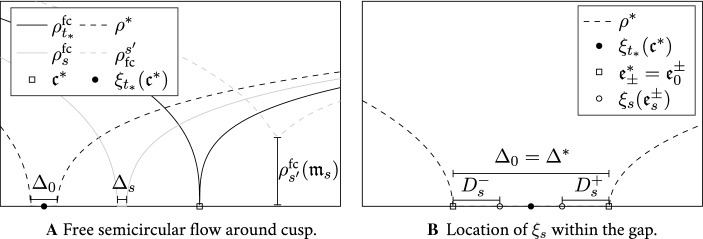
(**a**) illustrates the evolution of $\rho ^\text {fc}_s$ along the semicircular flow at two times $0<s<t_*<s'$ before and after the cusp. We recall that $\rho ^*=\rho ^\text {fc}_0$ and $\rho =\rho ^\text {fc}_{t_*}$. (**b**) shows the points $\xi _s(\mathfrak {e}_s^\pm )$ as well as their distances to the edges $\mathfrak {e}_0^\pm$

Figure 1a illustrates the quantities in the following lemma. We state the lemma for scDOSs from arbitrary data pairs $(A_*,\mathcal {S}_*)$ satisfying the conditions in [10], i.e.

5.4 $$\begin{aligned} \Vert A_*\Vert \le C, \qquad c\left\langle R\right\rangle \le \mathcal {S}_*[R]\le C \left\langle R\right\rangle \end{aligned}$$

for any self-adjoint $R=R^*$ and some constants $c,C>0$.

#### Lemma 5.1

Let $\rho ^*$ be the density of a Stieltjes transform $m^*=\left\langle M_*\right\rangle$ associated with some Dyson equation

$$\begin{aligned} -1=(z-A_*+\mathcal {S}_*[M_*])M_*, \end{aligned}$$

with $(A_*,\mathcal {S}_*)$ satisfying (5.4). Then there exists a small constant *c*, depending only on the constants in Assumptions (5.4) such that the following statements hold true. Suppose that $\rho ^*$ has an initial gap $[\mathfrak {e}_-^*,\mathfrak {e}_+^*]$ of size $\Delta ^*= \mathfrak {e}_+^*-\mathfrak {e}_-^*\le c$. Then there exists some critical time $t_*\lesssim (\Delta ^*)^{2/3}$ such that $m_{t_*}^\text {fc}$ has exactly one exact cusp in some point $\mathfrak {c}^*$ with , and that $\rho _{t_*}^\text {fc}$ is locally around $\mathfrak {c}^*$ given by (2.4a) for some $\gamma >0$. Considering the time evolution $[0,2t_*]\ni s\mapsto m_s^\text {fc}$ we then have the following asymptotics.

(i) *After the cusp.* For $t_*<s\le 2t_*$, $\rho _s^\text {fc}$ has a unique non-zero local minimum in some point $\mathfrak {m}_s$ such that
  5.5a $$\begin{aligned} \rho _s^\text {fc}(\mathfrak {m}_s)= & {} \frac{\sqrt{s-t_*}\gamma ^2}{\pi }[1+{\mathcal {O}}\,\left( [\right) 0]{(s-t_*)^{1/2}}],\nonumber \\&\qquad \left| \mathfrak {m}_s-\mathfrak {c}^*+(s-t_*)\mathfrak {R}m_s^\text {fc}(\mathfrak {m}_s)\right| \lesssim (s-t_*)^{3/2+1/4}. \end{aligned}$$
  Furthermore, $\mathfrak {m}_s$ can approximately be found by solving a simple equation, namely there exists $\widetilde{\mathfrak {m}}_s$ such that 5.5b
(ii) *Before the cusp.* For $0\le s<t_*$, the support of $\rho _s^\text {fc}$ has a spectral gap $[\mathfrak {e}_s^-,\mathfrak {e}_s^+]$ of size $\Delta _{s}:=\mathfrak {e}_s^+-\mathfrak {e}_s^-$ near $\mathfrak {c}^*$ which satisfies
  5.5c $$\begin{aligned} \Delta _s = (2\gamma )^2 \Big (\frac{t_*-s}{3}\Big )^{3/2}[1+{\mathcal {O}}\,\left( [\right) 0]{(t_*-s)^{1/3}}]. \end{aligned}$$
  In particular we find that the initial gap $\Delta ^*=\Delta _0$ is related to $t_*$ via $\Delta ^*= (2\gamma )^2 (t_*/3)^{3/2}[1+{\mathcal {O}}\,\left( [\right) 0]{(t_*-s)^{1/3}}]$.

#### Proof

Within the proof of the lemma we rely on the extensive shape analysis from [10]. We are doing so not only for the density $\rho ^*=\rho _0^\text {fc}$ and its Stieltjes transform, but also for $\rho _s^\text {fc}$ and its Stieltjes transform $m_s^\text {fc}$ for $0\le s\le 2t_*$. The results from [10] also apply here since $m_s^\text {fc}(z)=\left\langle M_*(\xi _s(z))\right\rangle$ can also be realized as the solution

$$\begin{aligned} -M_*(\xi _s(z))^{-1}= & {} z + s\left\langle M_*(\xi _s(z))\right\rangle -A_*+ \mathcal {S}_*[M_*(\xi _s(z))] \\= & {} z -A_*+ (\mathcal {S}_*+s\mathcal {S}^{\mathrm {GUE}})[M_*(\xi _s(z))] \end{aligned}$$

to the Dyson equation with perturbed self-energy $\mathcal {S}_*+ s\mathcal {S}^{\mathrm {GUE}}$. Since $t_*\lesssim 1$ it follows that the shape analysis from [10] also applies to $\rho _s^\text {fc}$ for any $s\in [0,2t_*]$.

We begin with part (i). Set $\nu :=s-t_*$, then for $0\le \nu \le t_*$ we want to find $x_\nu$ such that $\mathfrak {I}m^\text {fc}_s$ has a local minimum in $\mathfrak {m}_s:=\mathfrak {c}^*+x_\nu$ near $\mathfrak {c}^*$, i.e.

$$\begin{aligned} x_\nu :={{\,\mathrm{arg~min}\,}}_x \mathfrak {I}m^\text {fc}_s(\mathfrak {c}^*+x), \qquad \left| x_\nu \right| \lesssim \nu . \end{aligned}$$

First we show that $x_\nu$ with these properties exists and is unique by using the extensive shape analysis in [10]. Uniqueness directly follows from [10, Theorem 7.2(ii)]. For the existence, we set

$$\begin{aligned} a_\nu (x):=\mathfrak {I}m_{\text {fc}}^s(\mathfrak {c}^*+x), \quad b_\nu (x) :=\mathfrak {R}m^\text {fc}_s(\mathfrak {c}^*+x), \quad a_\nu :=a_\nu (x_\nu ), \quad b_\nu :=b_\nu (x_\nu ). \end{aligned}$$

Set $\delta :=K\nu$ with a large constant *K*. Since $a_0(x)=\mathfrak {I}m_{t_*}(\mathfrak {c}^*+x)\sim \left| x\right| ^{1/3}$, we have $a_0(\pm \delta )\sim \delta ^{1/3}$ and $a_0(0)=0$. Recall from [10, Proposition 10.1(a)] that the map $s\mapsto m^\text {fc}_s$ is 1/3-Hölder continuous. It then follows that $a_\nu (\pm \delta )\sim \delta ^{1/3} + {\mathcal {O}}\,\left( \nu ^{1/3}\right)$, while $a_\nu (0)\lesssim \nu ^{1/3}$. Thus $a_\nu$ necessarily has a local minimum in $(-\delta ,\delta )$ if *K* is sufficiently large. This shows the existence of a local minimum with $\left| x_\nu \right| \lesssim K\nu \sim \nu$.

We now study the function $f_\nu (x)=x+\nu b_\nu (x)$ in a small neighbourhood around 0. From [10, Eqs. (7.62),(5.43)–(5.45)] it follows that

5.6 $$\begin{aligned} \begin{aligned} b_\nu '(x)&= \mathfrak {R}\frac{c_1(x)+{\mathcal {O}}\,\left( a_\nu (x)\right) }{-\mathrm {i}c_2(x) a_\nu (x) + a_\nu (x)^2+{\mathcal {O}}\,\left( a_\nu (x)^3\right) } +{\mathcal {O}}\,\left( 1\right) \\&= \frac{c_1(x)}{c_2(x)^2 + a_\nu (x)^2} + {\mathcal {O}}\,\left( \frac{1}{c_2(x)+a_\nu (x)}\right) \end{aligned} \end{aligned}$$

whenever $a_\nu (x)\ll 1$, with appropriate real functions^3^$c_1(x)\sim 1$ and $c_2(x)\ge 0$. Moreover, $\left| c_2(0)\right| \ll 1$ since $\mathfrak {c}^*$ is an almost cusp point for $m_s^\text {fc}$ for any $s\in [0,2t_*]$. Thus it follows that $b_\nu '(x)>0$ whenever $a_\nu (x)+c_2(x)\ll 1$. Due to the 1/3-Hölder continuity^4^ of both $a_\nu (x)$ and $c_2(x)$ and $a_\nu (0)+ \left| c_2(0)\right| \ll 1$, it follows that $b_\nu '(x)>0$ whenever $\left| x\right| \ll 1$. We can thus conclude that $f_\nu$ satisfies $f_\nu '\ge 1$ in some ${\mathcal {O}}\,\left( 1\right)$-neighbourhood of 0. As $\left| f_\nu (0)\right| \lesssim \nu$ we can conclude that there exists a root $\widetilde{x}_\nu$, $f_\nu (\widetilde{x}_\nu )=0$ of size . With $\widetilde{\mathfrak {m}}_s:=\mathfrak {c}^*+\widetilde{x}_\nu$ we have thus shown the first equality in (5.5b).

Using (2.4a), we now expand the defining equation

$$\begin{aligned} a_\nu (x) = \mathfrak {I}m_{t_*}^\text {fc}(\mathfrak {c}^*+x+\nu b_\nu (x) +\mathrm {i}\nu a_\nu (x) ) \end{aligned}$$

for the free convolution in the regime for those *x* sufficiently close to $\widetilde{x}_\nu$ such that  to find

$$\begin{aligned} \begin{aligned} a_\nu (x)&=\frac{\sqrt{3} \gamma ^{4/3}}{2\pi } \nu a_\nu (x) \int _\mathbb {R}\frac{\left| \lambda \right| ^{1/3}+{\mathcal {O}}\,\left( \left| \lambda \right| ^{2/3}\right) }{(\lambda -x-\nu b_\nu (x))^2+ (\nu a_\nu (x))^2}\mathrm{d}\lambda \\&= \frac{\sqrt{3} \gamma ^{4/3}}{2\pi } \int _\mathbb {R}\frac{ (\nu a_\nu (x))^{1/3} \left| \lambda \right| ^{1/3}}{(\lambda -[x+\nu b_\nu (x)]/\nu a_\nu (x))^2+1}\mathrm{d}\lambda + {\mathcal {O}}\,\left( (\nu a_\nu (x))^{2/3}\right) \\&= (\nu a_\nu (x))^{1/3} \gamma ^{4/3} \left[ 1+ \frac{1}{9}\left( \frac{x+\nu b_\nu (x)}{\nu a_\nu (x)}\right) ^2 + {\mathcal {O}}\,\left( \left( \frac{x+\nu b_\nu (x)}{\nu a_\nu (x)}\right) ^4 + (\nu a_\nu (x))^{1/3}\right) \right] , \end{aligned} \end{aligned}$$

i.e.

5.7 $$\begin{aligned} a_\nu (x) = \nu ^{1/2}\gamma ^2 \left[ 1+ \frac{1}{9}\left( \frac{x+\nu b_\nu (x)}{\nu a_\nu (x)}\right) ^2 + {\mathcal {O}}\,\left( \left( \frac{x+\nu b_\nu (x)}{\nu a_\nu (x)}\right) ^4 + (\nu a_\nu (x))^{1/3}\right) \right] ^{3/2}. \nonumber \\ \end{aligned}$$

Note that (5.7) implies that $\nu a_\nu (\widetilde{x}_\nu )\sim \nu ^{3/2}$, i.e. the last claim in (5.5b). We now pick some large *K* and note that from (5.7) it follows that $a_\nu (\widetilde{x}_\nu \pm K \nu ^{7/4})> a_\nu (\widetilde{x}_\nu )$. Thus the interval $[\widetilde{x}_\nu - K \nu ^{7/4}, \widetilde{x}_\nu + K \nu ^{7/4}]$ contains a local minimum of $a_\nu (x)$, but by the uniqueness this must then be $x_\nu$. We thus have , proving the second claim in (5.5b). By 1/3-Hölder continuity of $a_\nu (x)$ and by $a_\nu (\widetilde{x}_\nu )\sim \nu ^{1/2}$ from (5.7), we conclude that $a_\nu = a_\nu (x_\nu )\sim \nu ^{1/2}$ as well. Using that $\widetilde{x}_\nu + \nu b_\nu (\widetilde{x}_\nu )=0$ and $b_\nu '\lesssim 1/\nu$ from (5.6) and $a_\nu (x)\gtrsim \sqrt{\nu }$, we conclude that $\left| x_\nu +\nu b_\nu (x_\nu )\right| \lesssim \nu ^{7/4}$, i.e. the second claim in (5.5a). Plugging this information back into (5.7), we thus find $a_\nu =\gamma ^2\sqrt{\nu }(1+{\mathcal {O}}\,\left( \nu ^{1/2}\right) )$ and have also proven the first claim in (5.5a).

We now turn to part (5.5). It follows from the analysis in [10] that $\rho ^\text {fc}_s$ exhibits either a small gap, a cusp or a small local minimum close to $\mathfrak {c}^*$. It follows from (i) that a cusp is transformed into a local minimum, and a local minimum cannot be transformed into a cusp along the semicircular flow. Therefore it follows that the support of $\rho ^\text {fc}_s$ has a gap of size $\Delta _s=\mathfrak {e}_s^+-\mathfrak {e}_s^-$ between the edges $\mathfrak {e}_s^\pm$. Evidently $\mathfrak {e}_{t_*}^-=\mathfrak {e}_{t_*}^+=\mathfrak {c}^*$, $\mathfrak {e}_0^+-\mathfrak {e}_0^-=\Delta _0$, $\mathfrak {e}_0^\pm =\mathfrak {e}_\pm ^*$ and for $s>0$ we differentiate (5.3a) to obtain

5.8 $$\begin{aligned} \frac{(m^\text {fc}_s)'(z)}{1+s(m^\text {fc}_s)'(z)} = m_*'(z+sm^\text {fc}_s (z))\quad \text {and conclude}\quad m_*'(\xi _s(\mathfrak {e}_s^\pm ))=1/s \end{aligned}$$

by considering the $z\rightarrow \mathfrak {e}_s^\pm$ limit and the fact that $\rho ^\text {fc}_s$ has a square root at edge (for $s<t_*$) hence $(m^\text {fc}_s)'$ blows up at this point. Denoting the $\mathrm{d}/\mathrm{d}s$ derivative by dot, from

$$\begin{aligned} \frac{\mathrm{d}}{\mathrm{d}s} m^\text {fc}_s(\mathfrak {e}_s^\pm ) = m_*'(\xi _s(\mathfrak {e}_s^\pm ))\left( {\dot{\mathfrak {e}}}_s^\pm + m^\text {fc}_s(\mathfrak {e}_s^\pm ) +s\frac{\mathrm{d}}{\mathrm{d}s} m^\text {fc}_s(\mathfrak {e}_s^\pm ) \right) = \frac{{\dot{\mathfrak {e}}}_s^\pm + m^\text {fc}_s(\mathfrak {e}_s^\pm )}{s}+ \frac{\mathrm{d}}{\mathrm{d}s} m^\text {fc}_s(\mathfrak {e}_s^\pm ) \end{aligned}$$

we can thus conclude that ${\dot{\mathfrak {e}}}_s^\pm =-m^\text {fc}_s(\mathfrak {e}_s^\pm )$. This implies that the gap as a whole moves with linear speed (for non-zero $m^\text {fc}_s(\mathfrak {e}_s^\pm )$), and, in particular, the distance of the gap of $\rho ^*$ to $\mathfrak {c}^*$ is an order of magnitude larger than the size of the gap. It follows that the size $\Delta _{s}:=\mathfrak {e}_s^+-\mathfrak {e}_s^-$ of the gap of $\rho ^\text {fc}_s$ satisfies

$$\begin{aligned} {\dot{\Delta }}_{s}=m^\text {fc}_s(\mathfrak {e}_s^-)-m^\text {fc}_s(\mathfrak {e}_s^+)=\int _\mathbb {R}\Big [\frac{1}{x-\mathfrak {e}_s^-}-\frac{1}{x-\mathfrak {e}_s^+}\Big ]\rho ^\text {fc}_s(x)\mathrm{d}x=-\Delta _{s}\int _\mathbb {R}\frac{\rho ^\text {fc}_s(x)}{(x-\mathfrak {e}_s^-)(x-\mathfrak {e}_s^+)}\mathrm{d}x. \end{aligned}$$

We now use the precise shape of $\rho ^\text {fc}_s$ close to $\mathfrak {e}_s^\pm$ according to (2.4b) which is given by

5.9 $$\begin{aligned} \rho ^\text {fc}_s(\mathfrak {e}_s^\pm \pm x)= & {} \frac{\sqrt{3}(2\gamma )^{4/3}\Delta _{s}^{1/3}}{2\pi }\nonumber \\&\left( (1+{\mathcal {O}}\,\left( [\right) 0]{(t_*-t)^{1/3}})\Psi _{\text {edge}}(x/\Delta _{s})+{\mathcal {O}}\,\left( \Delta _s^{1/3}\Psi ^2_{\text {edge}}(x/\Delta _{s})\right) \right) ,\nonumber \\ \end{aligned}$$

where $\Psi _\mathrm {edge}$ defined in (2.4c) exhibits the limiting behaviour

$$\begin{aligned} \lim _{\Delta \rightarrow 0}\Delta ^{1/3}\Psi _{\text {edge}}(x/\Delta ) = \left| x\right| ^{1/3}/2^{4/3}. \end{aligned}$$

Using (5.9), we compute

5.10 $$\begin{aligned} \begin{aligned} {\dot{\Delta }}_{s}&=-(1+{\mathcal {O}}\,\left( [\right) 0]{(t_*-s)^{1/3}})\frac{\sqrt{3}(2\gamma )^{4/3}\Delta _{s}^{1/3}}{\pi } \int _0^\infty \frac{\Psi _{\text {edge}}(x)}{x(1+x)}\mathrm{d}x \\&= -\gamma ^{4/3}(2\Delta _{s})^{1/3}\left[ 1+{\mathcal {O}}\,\left( [\right) 0]{(t_*-s)^{1/3}+\Delta _{s}^{1/3}}\right] , \end{aligned} \end{aligned}$$

where the $(1+{\mathcal {O}}\,\left( [\right) 0]{(t_*-s)^{1/3}})$ factor in (5.9) encapsulates two error terms; both are due to the fact that the shape factor $\gamma _s$ of $\rho _s^\text {fc}$ from (2.4b) is not exactly the same as $\gamma$, i.e. the one for $s=t_*$. To track this error in $\gamma$ we go back to [10]. First, $\left| \sigma \right|$ in [10, Eq. (7.5a)] is of size $(t_*-s)^{1/3}$ by the fact that $\sigma$ vanishes at $s=t_*$ and is 1/3-Hölder continuous according to [10, Lemma 10.5]. Secondly, according to [10, Lemma 10.5] the shape factor $\Gamma$ (which is directly related to $\gamma$ in the present context) is also 1/3-Hölder continuous and therefore we know that the shape factors of $\rho ^*$ at $\mathfrak {e}_0^\pm$ are at most multiplicatively perturbed by a factor of $(1+{\mathcal {O}}\,\left( [\right) 0]{(t_*-s)^{1/3}})$. By solving the differential equation (5.10) with the initial condition $\Delta _{t_*}=0$, the claim (5.5c) follows. $\square$

Besides the asymptotic expansion for gap size and local minimum we also require some quantitative control on the location of $\xi _{t_*}(\mathfrak {c}^*)$, as defined in (5.3a), and some slight perturbations thereof within the spectral gap $[\mathfrak {e}_-^*,\mathfrak {e}_+^*]$ of $\rho ^*$. We remark the the point $\xi ^*:=\xi _{t_*}(\mathfrak {c}^*)$ plays a critical role for the contour integration in Sect. 5.2 since it will be the critical point of the phase function. From (5.5c) we recall that the gap size scales as $t_*^{3/2}$ which makes it natural to compare distances on that scale. In the regime where $t'\ll t_*$ all of the following estimates thus identify points very close to the centre of the initial gap.

#### Lemma 5.2

Suppose that we are in the setting of Lemma 5.1. We then find that $\xi _{t_*}(\mathfrak {c}^*)$ is very close to the centre of $[\mathfrak {e}_-^*,\mathfrak {e}_+^*]$ in the sense that 5.11aFurthermore, for $0\le t'\le t_*$ we have that5.11b

#### Proof

We begin with proving (5.11a). For $s<t_*$ we denote the distance of $\xi _s(\mathfrak {e}_s^\pm )$ to the edges $\mathfrak {e}_0^\pm$ by $D_s^\pm :=\pm (\mathfrak {e}_0^\pm - \xi _s(\mathfrak {e}_s^\pm ))$, cf. Fig. 1b. We have, by differentiating $m_*'(\xi _s(\mathfrak {e}_s^\pm ))=1/s$ from (5.8) that

5.12 $$\begin{aligned} \dot{D}_{s}^\pm =\mp \frac{\mathrm{d}}{\mathrm{d}s}\xi _s(\mathfrak {e}_s^\pm ),\qquad -\frac{1}{s^2}= m''_*(\xi _s(\mathfrak {e}_s^\pm )) \frac{\mathrm{d}}{\mathrm{d}s}\xi _s(\mathfrak {e}_s^\pm ) \end{aligned}$$

and by differentiating (5.3a),

$$\begin{aligned} (m^\text {fc}_s)'= m_*'(\xi _s) \xi _s',\quad \xi _s'(m^\text {fc}_s)''= m_*''(\xi _s)(\xi _s')^3+(m^\text {fc}_s)'\xi _s'',\quad m_*''(\xi _s)=\frac{(m^\text {fc}_s)''}{(1+s (m^\text {fc}_s)')^3}. \end{aligned}$$

We now consider $z=\mathfrak {e}_s^\pm +\mathrm {i}\eta$ with $\eta \rightarrow 0$ and compute from (5.9), for any $s<t_*$,

$$\begin{aligned} \begin{aligned} \lim _{\eta \searrow 0}\sqrt{\eta }(m^\text {fc}_s)'(z)&=\lim _{\eta \searrow 0}\sqrt{\eta }\int _\mathbb {R}\frac{\rho ^\text {fc}_s(x)}{(x-z)^2}\mathrm{d}x =\lim _{\eta \searrow 0}\frac{\sqrt{3\eta }(2\gamma )^{4/3}\Delta _{s}^{1/3}}{2\pi }\int _0^\infty \frac{\Psi _\text {edge}(x/\Delta _{s})}{(x-\mathrm {i}\eta )^2}\mathrm{d}x\\&= \frac{(2\gamma )^{4/3}}{2\sqrt{3}\Delta _{s}^{1/6}\pi }\int _0^\infty \frac{x^{1/2}}{(x-\mathrm {i})^2}\mathrm{d}x = \frac{(2\gamma )^{4/3}\sqrt{\mathrm {i}}}{4\sqrt{3}\Delta _{s}^{1/6}} \end{aligned} \end{aligned}$$

and

$$\begin{aligned} \begin{aligned} \lim _{\eta \searrow 0}\eta ^{3/2}(m^\text {fc}_s)''(z)&=\lim _{\eta \searrow 0}\eta ^{3/2}2\int _\mathbb {R}\frac{\rho ^\text {fc}_s(x)}{(x-z)^3}\mathrm{d}x\\&= \lim _{\eta \searrow 0}\frac{\sqrt{3\eta }^{3/2}(2\gamma )^{4/3}\Delta _{s}^{1/3}}{\pi }\int _0^\infty \frac{\Psi _\text {edge}(x/\Delta _{s})}{(x-\mathrm {i}\eta )^3}\mathrm{d}x\\&=\frac{(2\gamma )^{4/3}}{\sqrt{3} \Delta _{s}^{1/6}\pi }\int _0^\infty \frac{x^{1/2}}{(x-\mathrm {i})^3}\mathrm{d}x = \frac{(2\gamma )^{4/3}\mathrm {i}^{3/2}}{8\sqrt{3}\Delta _{s}^{1/6}}. \end{aligned} \end{aligned}$$

Here we used that fact that the error terms in (5.9) become irrelevant in the $\eta \rightarrow 0$ limit. We conclude, together with (5.12), that

$$\begin{aligned} \begin{aligned} m_*''(\xi _s(\mathfrak {e}_s^\pm ))&= \pm \frac{3(2\Delta _{s})^{1/3}}{s^3\gamma ^{8/3}},\\ \dot{D}_s^\pm&=\pm (s^2 m_*''(\xi _s(\mathfrak {e}_s^\pm )))^{-1}=\frac{s\gamma ^{8/3}}{3(2\Delta _{s})^{1/3}}= \frac{s\gamma ^2}{2\sqrt{3}\sqrt{t_*-s}}[1+{\mathcal {O}}\,\left( [\right) 0]{t_*^{1/3}}]. \end{aligned} \end{aligned}$$

Since $D_0^-=D_0^+=0$ and $\dot{D}_s^-\approx \dot{D}_s^+$ it follows that, to leading order, $D_{s}^+\approx D_{s}^-$ and more precisely

$$\begin{aligned} D_s^\pm = \gamma ^2 \frac{2t_*^{3/2} -s \sqrt{t_*-s}-2t_*\sqrt{t_*-s}}{3^{3/2}}[1+{\mathcal {O}}\,\left( [\right) 0]{t_*^{1/3}}]. \end{aligned}$$

In particular it follows that $\left| \mathfrak {e}_0^\pm -\xi _{t_*}(\mathfrak {c}^*)\right| = [1+{\mathcal {O}}\,\left( [\right) 0]{t_*}^{1/3}]2\gamma ^2 t_*^{3/2} /3^{3/2}$. Together with the $s=0$ case from (5.5c) we thus findproving (5.11a).

We now turn to the proof of (5.11b) where we treat the small gap and small non-zero minimum separately. We start with the first inequality. We observe that (5.11a) in the setting where $(\rho ^*,t_*)$ are replaced by $(\rho _{t_*-t'}^\text {fc},t')$ implies5.13Furthermore, we infer from the definition of $\xi$ and the associativity (5.3b) of the free convolution that

$$\begin{aligned} \xi _{t_*-t'}\Big ( \mathfrak {c}^*+ t' m_{t_*}^\text {fc}(\mathfrak {c}^*) \Big ) = \mathfrak {c}^*+ t' m_{t_*}^\text {fc}(\mathfrak {c}^*) + (t_*-t') m_{t_*-t'}^\text {fc}\Big ( \mathfrak {c}^*+ t' m_{t_*}^\text {fc}(\mathfrak {c}^*) \Big ) = \xi _{t_*}(\mathfrak {c}^*) \end{aligned}$$

and can therefore estimatejust as claimed. In the last step we used (5.13) and the fact that

5.14 $$\begin{aligned} \left| \xi _s(a)-\xi _s(b)\right| \lesssim \left| a-b\right| + s \left| a-b\right| ^{1/3}, \end{aligned}$$

which directly follows from the definition of $\xi$ and the 1/3-Hölder continuity of $m_s^\text {fc}$.

Finally, we address the second inequality in (5.11b) and appeal to Lemma 5.1(i) to establish the existence of $\widetilde{\mathfrak {m}}_{t_*+t'}$ such that

5.15 $$\begin{aligned} \mathfrak {c}^*-\widetilde{\mathfrak {m}}_{t_*+t'} = t' \mathfrak {R}m_{t_*+t'}^\text {fc}(\widetilde{\mathfrak {m}}_{t_*+t'}). \end{aligned}$$

It thus follows from (5.5b) that  and therefore from (5.14) thatUsing (5.15) twice, as well as the associativity (5.3b) of the free convolution and $\mathfrak {I}m_{t_*}^\text {fc}(\mathfrak {c}^*)=0$ we then further compute

5.16 $$\begin{aligned} \begin{aligned}&\xi _{t_*+t'}(\widetilde{\mathfrak {m}}_{t_*+t'})-\xi _{t_*}(\mathfrak {c}^*) = \widetilde{\mathfrak {m}}_{t_*+t'} + (t_{*}+t') m_{t_*+t'}^\text {fc}(\widetilde{\mathfrak {m}}_{t_*+t'})-\mathfrak {c}^*- t_*m_{t_*}^\text {fc}(\mathfrak {c}^*) \\&\quad = t_*\mathfrak {R}\Big [ m_{t_*}^\text {fc}(\mathfrak {c}^*+\mathrm {i}t' \mathfrak {I}m_{t_*+t'}^\text {fc}(\widetilde{\mathfrak {m}}_{t_*+t'}))- m_{t_*}^\text {fc}(\mathfrak {c}^*) \Big ] +\mathrm {i}(t_{*}+t')\mathfrak {I}m_{t_*+t'}^\text {fc}(\widetilde{\mathfrak {m}}_{t_*+t'}). \end{aligned}\qquad \end{aligned}$$

By Hölder continuity we can, together with (5.11a) and $\mathfrak {I}m_{t_*+t'}(\widetilde{\mathfrak {m}}_{t_*+t'}) \sim (t')^{1/2}$ from (5.5b), conclude thatIn the first term we used (5.14) and the second estimate of (5.5b). In the second term we used (5.16) together with $\mathfrak {I}m_{t_*+t'}(\widetilde{\mathfrak {m}}_{t_*+t'})\sim (t')^{1/2}$ from (5.5b) and 1/3-Hölder continuity of $m_{t_*}^\text {fc}$. Finally, the last term was already estimated in the exact cusp case, i.e. in (5.11a). $\square$

### Correlation kernel as contour integral

We denote the eigenvalues of $H_t$ by $\lambda _1,\ldots ,\lambda _N$. Following the work of Brézin and Hikami (see e.g. [22, Eq. (2.14)] or [35, Eq. (3.13)] for the precise version used in the present context) the correlation kernel of $\widetilde{H}_t=H_t+\sqrt{ct} U$ can be written as

$$\begin{aligned}&\widehat{K}_N^t(u,v) :=\frac{N}{(2\pi \mathrm {i})^2 ct} \int _\Upsilon \mathrm{d}z \int _\Gamma \mathrm{d}w \frac{\exp \left( N\left[ w^2-2vw+v^2 -z^2 +2zu-u^2\right] /2ct\right) }{w-z}\\&\quad \prod _{i}\frac{w-\lambda _i}{z-\lambda _i}, \end{aligned}$$

where $\Upsilon$ is any contour around all $\lambda _i$, and $\Gamma$ is any vertical line not intersecting $\Upsilon$. With this notation, the *k*-point correlation function of the eigenvalues of $\widetilde{H}_t$ is given by

$$\begin{aligned} p_k^{(N)}(x_1,\ldots ,x_k)=\det \Big (\frac{1}{N}\widehat{K}_N^t(x_i,x_j)\Big )_{i,j\in [k]}. \end{aligned}$$

Due to the determinantal structure we can freely conjugate $K_N$ with $v\mapsto e^{N(\xi v-v^2/2)/ct}$ for $\xi :=\xi _{ct}(\mathfrak {b})$ to redefine the correlation kernel as

$$\begin{aligned}&K_N^t(u,v) :=\frac{N}{(2\pi \mathrm {i})^2 c t} \int _\Upsilon \mathrm{d}z \int _\Gamma \mathrm{d}w \frac{\exp \left( N\left[ w^2-2v(w-\xi ) -z^2+2u(z-\xi )\right] /2ct\right) }{w-z}\\&\quad \prod _{i}\frac{w-\lambda _i}{z-\lambda _i}. \end{aligned}$$

This redefinition $K_N^t$ does not agree point-wise with the previous definition $\widehat{K}_N^t$, but gives rise to the same determinant, and in particular to the same *k*-point correlation function. Here $\mathfrak {b}$ is the base point chosen in Theorem 2.3. The central result concerning the correlation kernel is the following proposition.

#### Proposition 5.3

Under the assumptions of Theorem 2.3, the rescaled correlation kernel

5.17 $$\begin{aligned} \widetilde{K}_N^t(x,y):=\frac{1}{N^{3/4}\gamma } K_N^t\left( \mathfrak {b}+\frac{x}{N^{3/4}\gamma },\mathfrak {b}+\frac{y}{N^{3/4}\gamma }\right) \end{aligned}$$

around the base point $\mathfrak {b}$ chosen in (2.6) converges uniformly to the Pearcey kernel from (2.5) in the sense that

$$\begin{aligned} \left| \widetilde{K}_N^t(x,y)-K_\alpha (x,y)\right| \le C N^{-c} \end{aligned}$$

for $x,y\in [-R,R]$. Here *R* is an arbitrary large threshold, $c>0$ is some universal constant, $C>0$ is a constant depending only on the model parameters and *R*, and $\alpha$ is chosen according to (2.6).

#### Proof

We now split the contour $\Upsilon$ into two parts, one encircling all eigenvalues $\lambda _i$ to the left of $\xi =\mathfrak {b}+ct\left\langle M(\mathfrak {b})\right\rangle$, and the other one encircling all eigenvalues $\lambda _i$ to the right of $\xi$, which does not change the value of $K_N^t$. We then move the vertical $\Gamma$ contour so that it crosses the real axis in $\xi$. This does also not change the value $K_N^t$ as the only pole is the one in *z* for which the residue reads

$$\begin{aligned} \frac{N}{(2\pi \mathrm {i})^2 ct}\int _\Upsilon \mathrm{d}z \exp \left( \frac{N}{ct\gamma }(u-v)(z-\xi )\right) =0. \end{aligned}$$

We now perform a linear change of variables $z\mapsto \xi +\Delta _0 z$, $w\mapsto \xi + \Delta _0 w$ in (5.17) to transform the contours $\Upsilon ,\Gamma$ into contours

5.18 $$\begin{aligned} \widehat{\Gamma } :=(\Gamma -\xi )/\Delta _0,\qquad \widehat{\Upsilon } :=(\Upsilon -\xi )/\Delta _0 \end{aligned}$$

to obtain

5.19 $$\begin{aligned} \widetilde{K}_N^t(x,y)= \frac{N^{1/4}\Delta _0}{(2\pi \mathrm {i})^2ct\gamma } \int _{{\widehat{\Upsilon }}}\mathrm{d}z\int _{{\widehat{\Gamma }}} \mathrm{d}w \frac{\exp \left( \Delta _0 N^{1/4} (xz-yw)/ct\gamma + N\Delta _0^2[\widetilde{f}(w)-\widetilde{f}(z)]/ct\right) }{w-z},\nonumber \\ \end{aligned}$$

where

$$\begin{aligned} \widetilde{f}(z) :=\frac{z^2}{2}-\frac{ct}{\Delta _0^2}\int _\xi ^{\xi +\Delta _0 z} \left\langle G_t(u)-M_t(\xi )\right\rangle \mathrm{d}u. \end{aligned}$$

Here $\Delta _0:=\mathfrak {e}_0^+-\mathfrak {e}_0^-$ indicates the length of the gap $[\mathfrak {e}_0^-,\mathfrak {e}_0^+]$ in the support of $\rho _t$. From Lemma 5.1 with $\rho ^*=\rho _t$ and $t_*=ct$ we infer $\Delta _0\sim t^{3/2}\sim N^{-3/4+3\epsilon /2}$. In order to obtain (5.19) we used the relation $\xi -\mathfrak {b}=ct m^\text {fc}_{ct}(\mathfrak {b})=ct \left\langle M_t(\mathfrak {b}+ct m^\text {fc}_{ct}(\mathfrak {b}))\right\rangle =ct \left\langle M_t(\xi )\right\rangle$.

We begin by analysing the deterministic variant of $\widetilde{f}(z)$,

$$\begin{aligned} f(z):=\frac{z^2}{2}-\frac{ct}{\Delta _0^2}\int _\xi ^{\xi +\Delta _0 z} \left\langle M_t(u)-M_t(\xi )\right\rangle \mathrm{d}u. \end{aligned}$$

We separately analyse the large- and small-scale behaviour of *f*(*z*). On the one hand, using the 1/3-Hölder continuity of $u\mapsto \left\langle M_t(u)\right\rangle$, eq. (5.5c) and

$$\begin{aligned} \frac{ct}{\Delta _0^2}\int _\xi ^{\xi +\Delta _0 z} \left| \left\langle M_t(u)-M_t(\xi )\right\rangle \right| \mathrm{d}u \lesssim \frac{t(\Delta _0\left| z\right| )^{4/3}}{\Delta _0^2} \lesssim \left| z\right| ^{4/3}. \end{aligned}$$

we conclude the large-scale asymptotics

5.20 $$\begin{aligned} f(z)=\frac{z^2}{2}+{\mathcal {O}}\,\left( \left| z\right| ^{4/3}\right) , \qquad \left| z\right| \gg 1. \end{aligned}$$

We now turn to the small-scale $\left| z\right| \ll 1$ asymptotics. We first specialize Lemmas 5.1 and 5.2 to $\rho ^*=\rho _t$ and collect the necessary conclusions in the following Lemma.

#### Lemma 5.4

Under the assumptions of Theorem 2.3 it follows that $\rho _t$ has a spectral gap $[\mathfrak {e}_0^-,\mathfrak {e}_0^+]$ of size

5.21a $$\begin{aligned}&\Delta _0 = \mathfrak {e}_0^+-\mathfrak {e}_0^-= \Delta (ct \pm t^\rho ) \left[ 1+{\mathcal {O}}\,\left( t^{1/3}\right) \right] ,\quad \text {where}\quad \pm t^\rho \nonumber \\&\quad :={\left\{ \begin{array}{ll} 0 &{}\text {in case (i)}\\ 3 (\Delta ^\rho )^{2/3} /(2\gamma )^{4/3} &{}\text {in case (ii)}\\ -\pi ^2 \rho (\mathfrak {m}^\rho )^2/\gamma ^4 &{}\text {in case (iii)}. \end{array}\right. } \end{aligned}$$

Furthermore, in all three cases we have that $\xi$ is is very close to the centre of the gap in the support of $\rho _t$ in the sense that

5.21b $$\begin{aligned} \left| \xi -\frac{\mathfrak {e}_0^++\mathfrak {e}_0^-}{2}\right| = {\mathcal {O}}\,\left( t^{3/2}N^{-\epsilon /2}\right) . \end{aligned}$$

#### Proof

We prove (5.21a)–(5.21b) separately in cases (i), (ii) and (iii).

(i) Here (5.21a) follows directly from (5.5c) with $\rho ^*=\rho _t$, $t_*=ct$, $s=0$ and $\mathfrak {c}^*=\mathfrak {c}^\rho$. Furthermore (5.21b) follows from (5.11a) with $\rho ^*=\rho _t$, $t_*=ct$ and $\mathfrak {c}^*=\mathfrak {c}^\rho$.
(ii) We apply (5.5c) with $\rho ^*=\rho =\rho _{ct}^\text {fc}$, $t_*=t^\rho$, $s=0$ to conclude that $\Delta ^\rho = (2\gamma )^2 (t^\rho /3)^{3/2}[1+{\mathcal {O}}\,\left( [\right) 0]{(t^\rho )^{1/3}}]$, and that $\rho _{ct+t^\rho }^\text {fc}$ has an exact cusp in some point $\mathfrak {c}$. Thus (5.21a) follows from another application of (5.5c) with $\rho ^*=\rho _t$, $t_*=ct+t^\rho$, $s=0$ and $\mathfrak {c}^*=\mathfrak {c}$. Furthermore, (5.21b) follows again from (5.11b) but this time with $\rho ^*=\rho _t$, $t_*=ct+t^\rho$, $t'=t^\rho$ and $\mathfrak {e}^\pm _{t_*-t'}=\mathfrak {e}^\rho _\pm$, and using that $t_*^{1/9}\le N^{-\epsilon /2}$ for sufficiently small $\epsilon$.
(iii) From (5.5a) with $\rho ^*=\rho _t$, $t_*=ct-t^\rho$, $s=ct$ to conclude $\rho (\mathfrak {m}^\rho )=[1+{\mathcal {O}}\,\left( [\right) 0]{(t^\rho )^{1/2}}]\gamma ^2 \sqrt{t^\rho }/\pi$, and that $\rho _{ct-t^\rho }$ has an exact cusp in some point $\mathfrak {c}$. Finally, (5.21b) follows again from (5.11b) but with $\rho ^*=\rho _t$, $t_*=ct-t^\rho$, $t'=t^\rho$ and $\mathfrak {m}_{t_*+t'}=\mathfrak {m}^\rho$, and using $t'/t_*\lesssim t^\rho /ct\lesssim N^{-\epsilon }$ and $t_*^{1/12}\le N^{-\epsilon /2}$ for sufficiently small $\epsilon$.$\square$

Equipped with Lemma 5.4 we can now turn to the small scale analysis of *f*(*z*) and write out the Stieltjes transform to find

$$\begin{aligned} \begin{aligned} f(z)&=\frac{z^2}{2}-\frac{ct}{\Delta _0^2}\int _{\mathbb {R}} \int _{\xi }^{\xi +\Delta _0 z} \frac{u-\xi }{(x-u)(x-\xi )}\rho _t(x)\mathrm{d}u\mathrm{d}x \\&= \frac{z^2}{2}-\frac{ct}{\Delta _0}\int _\mathbb {R}\int _{0}^{z} \frac{u}{(x-u)x}\rho _t(\xi +\Delta _0 x)\mathrm{d}u\mathrm{d}x. \end{aligned} \end{aligned}$$

Note that these integrals are not singular since $\rho _{t}(\xi +\Delta _0 x)$ vanishes for $\left| x\right| \le 1/2$. We now perform the *u* integration to find

5.22 $$\begin{aligned} f(z)= \frac{z^2}{2}-\frac{ct}{\Delta _0}\int _\mathbb {R}\left[ \log x -\log (x-z)-\frac{z}{x} \right] \rho _t(\xi +\Delta _0 x)\mathrm{d}x. \end{aligned}$$

By using the precise shape (5.9) (with $s=0$) of $\rho _t$ close to the edges $\mathfrak {e}_0^\pm$, and recalling the gap size from (5.21a) and location of $\xi$ from (5.21b) we can then write

5.23 $$\begin{aligned} f(z) = (1+{\mathcal {O}}\,\left( [\right) 0]{t^{1/3}})\widetilde{g}(z) + {\mathcal {O}}\,\left( \left| z\right| ^2 t^{1/3}\right) \end{aligned}$$

with

$$\begin{aligned} \widetilde{g}(z):=\frac{z^2}{2} - \frac{3\sqrt{3}}{2\pi (1\pm t^\rho /ct)} \int _\mathbb {R}\left[ \log x -\log (x-z)-\frac{z}{x} \right] \Psi _\text {edge}(\left| x\right| -1/2) \mathbb {1}_{\left| x\right| \ge 1/2}\mathrm{d}x \end{aligned}$$

being the leading order contribution. Here ± indicates that the formula holds for all three cases (i), (ii) and (iii) simultaneously, where $t^\rho =0$ in case (i). The contribution of the error term in (5.9) to the integral in (5.22) is of order ${\mathcal {O}}\,\left( [\right) 0]{\left| z\right| ^2 t^{1/2}}$ using that $\log x-\log (x-z)-z/x={\mathcal {O}}\,\left( [\right) 0]{\left| z/x\right| ^2}$ and that $\left| x\right| \ge 1/2$ on the support of $\rho _t(\xi +\Delta _0 x)$. By the explicit integrals

$$\begin{aligned} \frac{3\sqrt{3}}{2\pi }\int _0^\infty \frac{\Psi _\text {edge}(x)}{(x+1/2)^2}\mathrm{d}x = \frac{1}{2}, \qquad \frac{3\sqrt{3}}{2\pi }\int _0^\infty \frac{\Psi _\text {edge}(x)}{(x+1/2)^4}\mathrm{d}x = \frac{8}{27} \end{aligned}$$

and a Taylor expansion of the logarithm $\log (x-z)$ we find that the quadratic term $z^2/2$ almost cancels and we conclude the small-scale asymptotics

5.24 $$\begin{aligned} \widetilde{g}(z) = \left( \frac{\pm t^\rho }{ct}\frac{z^2}{2} - \frac{4z^4}{27}\right) \Big (1+{\mathcal {O}}\,\left( t^\rho /t\right) \Big )+{\mathcal {O}}\,\left( \left| z\right| ^5\right) , \qquad \left| z\right| \ll 1. \end{aligned}$$

### Contour deformations

We now argue that we can deform the contours $\Upsilon ,\Gamma$ and thereby via (5.18) the derived contours $\widehat{\Upsilon },\widehat{\Gamma }$, in a way which bounds the sign of $\mathfrak {R}g$ away from zero along the contours. Here *g*(*z*) is the *N*-independent variant of $\widetilde{g}(z)$ given by5.25The topological aspect of our argument is inspired by the approach in [42–44].

#### Lemma 5.5

For all sufficiently small $\delta >0$ there exists $K=K(\delta )$ such that the following holds true. The contours $\Upsilon ,\Gamma$ then can be deformed, without touching $({{\,\mathrm{supp}\,}}\rho _t+[-1,1]){\setminus }\{\xi \}$ or each other, in such a way that the rescaled contours $\widehat{\Upsilon },\widehat{\Gamma }$ defined in (5.18) satisfy $\mathfrak {R}g\ge K$ on $\widehat{\Upsilon }\cap \{\left| z\right| >\delta \}$ and $\mathfrak {R}g\le -K$ on $\widehat{\Gamma }\cap \{\left| z\right| >\delta \}$. Furthermore, locally around 0 the contours can be chosen in such a way that

5.26 $$\begin{aligned} \begin{aligned} \widehat{\Gamma }\cap \{z\in \mathbb {C}|\left| z\right| \le \delta \}&= (-\mathrm {i}\delta ,\mathrm {i}\delta ), \\ \widehat{\Upsilon }\cap \{z\in \mathbb {C}|\left| z\right| \le \delta \}&= (- \delta e^{\mathrm {i}\pi /4},\delta e^{\mathrm {i}\pi /4})\cup (-\delta e^{-\mathrm {i}\pi /4},\delta e^{-\mathrm {i}\pi /4}). \end{aligned} \end{aligned}$$

#### Proof

Just as in (5.24) we have the expansion

5.27 $$\begin{aligned} g(z) = - \frac{4z^4}{27}+{\mathcal {O}}\,\left( \left| z\right| ^5\right) , \qquad \left| z\right| \ll 1. \end{aligned}$$

**Fig. 2:**
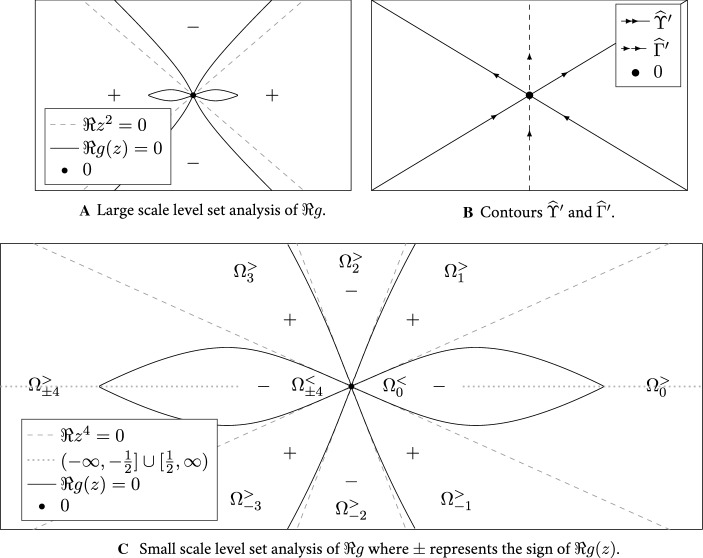
Representative cusp analysis. (a) and (c) show the level set $\mathfrak {R}g(z)=0$. On a small scale $g(z)\sim z^4$, while on a large scale $g(z)\sim z^2$. (**b**) shows the final deformed and rescaled contours $\widehat{\Upsilon }'$ and $\widehat{\Gamma }'$. (**c**) furthermore shows the cone sections $\Omega _k^>$ and $\Omega _k^{<}$, where we for clarity do not indicate the precise area thresholds given by $\delta$ and *R*. We also do not specifically indicate $\Omega _k^<$ for $k = \pm 1,\pm 2,\pm 3$ as then ${{\,\mathrm{cc}\,}}(\Omega _k^<)={{\,\mathrm{cc}\,}}(\Omega _k^>)$, cf. Claims 4–5 in the proof of Lemma 5.5

It thus follows that for some small $\delta >0$, andwe have $\Omega _{\pm 1}^<,\Omega _{\pm 3}^<\subset \Omega _+:=\{\mathfrak {R}g>0\}$ and $\Omega _{0}^<,\Omega _{\pm 2}^<,\Omega _4^<\subset \Omega _-:=\{\mathfrak {R}g<0\}$ in agreement with Fig. 2c. For large *z*, however, it also follows from (5.20) together with (5.25) and (5.23) that for some large *R*, and

$$\begin{aligned} \Omega _k^> :=\{z\in \mathbb {C}|\left| z\right| >R, \frac{(k-1)\pi }{4}+\delta<\arg z<\frac{(k+1)\pi }{4}+\delta \} \end{aligned}$$

we have $\Omega _0^>,\Omega _4^>\subset \Omega _+$ and $\Omega _{\pm 2}^>\subset \Omega _-$, in agreement with Fig. 2a. We denote the connected component of $\Omega _\pm$ containing some set *A* by ${{\,\mathrm{cc}\,}}(A)$.

- **Claim 1**—${{\,\mathrm{cc}\,}}(\Omega _{0}^>),{{\,\mathrm{cc}\,}}(\Omega _{4}^>)$ are **the only two unbounded connected components of** $\Omega _+$ Suppose there was another unbounded connected component *A* of $\Omega _+$. Since $\Omega _{\pm _2}^>\subset \Omega _-$ we would be able to find some $z_0\in A$ with arbitrarily large $\left| \mathfrak {R}z_0\right|$. If $\mathfrak {R}z_0>0$, then we note that the map $x\mapsto \mathfrak {R}g(z_0+x)$ is increasing, and otherwise we note that the map $x\mapsto \mathfrak {R}g(z_0-x)$ is increasing. Thus it follows in both cases that the connected component *A* actually coincides with ${{\,\mathrm{cc}\,}}(\Omega _0^>)$ or with ${{\,\mathrm{cc}\,}}(\Omega _4^>)$, respectively.
- **Claim 2**—${{\,\mathrm{cc}\,}}(\Omega _{\pm 2}^>)$**are the only two unbounded connected components of** $\Omega _-$ This follows very similarly to Claim 1.
- **Claim 3**—${{\,\mathrm{cc}\,}}(\Omega _{\pm 1}^<),{{\,\mathrm{cc}\,}}(\Omega _{\pm 2}^<),{{\,\mathrm{cc}\,}}(\Omega _{\pm 3}^<)$**are unbounded** We note that the map $z\mapsto \mathfrak {R}g(z)$ is harmonic on $\mathbb {C}{\setminus }([1/2,\infty )\cup (-\infty ,-1/2])$ and subharmonic on $\mathbb {C}$. Therefore it follows that ${{\,\mathrm{cc}\,}}(\Omega ^<_{\pm 1}),{{\,\mathrm{cc}\,}}(\Omega ^<_{\pm 3})\subset \Omega _+$ are unbounded. Since these sets are moreover symmetric with respect to the real axis it then also follows that ${{\,\mathrm{cc}\,}}(\Omega _{\pm 2})\cap ((-\infty ,-1/2]\cup [1/2,\infty ))=\emptyset$. This implies that $\mathfrak {R}g(z)$ is harmonic on ${{\,\mathrm{cc}\,}}(\Omega ^<_{\pm 2})$ and consequently also that ${{\,\mathrm{cc}\,}}(\Omega ^<_{\pm 2})$ are unbounded.
- **Claim 4**—${{\,\mathrm{cc}\,}}(\Omega _{1}^<)={{\,\mathrm{cc}\,}}(\Omega _{-1}^<)={{\,\mathrm{cc}\,}}(\Omega _0^>)$**and** ${{\,\mathrm{cc}\,}}(\Omega _{3}^<)={{\,\mathrm{cc}\,}}(\Omega _{-3}^<)={{\,\mathrm{cc}\,}}(\Omega _4^>)$ This follows from Claims 1–3.
- **Claim 5**—${{\,\mathrm{cc}\,}}(\Omega _2^<)={{\,\mathrm{cc}\,}}(\Omega _2^>)$**and** ${{\,\mathrm{cc}\,}}(\Omega _{-2}^<)={{\,\mathrm{cc}\,}}(\Omega _{-2}^>)$ This also follows from Claims 1–3.

The claimed bounds on $\mathfrak {R}g$ now follow from Claims 4–5 and compactness. The claimed small scale shape (5.26) follows by construction of the sets $\Omega ^<_k$. $\square$

From Lemmas 5.5 and 2.8 it follows that $K_N^t$ and thereby also $\widetilde{K}_N^t$ remain, with overwhelming probability, invariant under the chosen contour deformation. Indeed, $K_N^t$ only has poles where $z=w$ or $z=\lambda _i$ for some *i*. Due to self-adjointness and Lemma 5.5, $z=\lambda _i$ can only occur if $\lambda _i=\xi$ or ${{\,\mathrm{dist}\,}}(\lambda _i,{{\,\mathrm{supp}\,}}\rho _t)>1$. Both probabilities are exponentially small as a consequence of Lemma 2.8, since for the former we have $\eta _{\mathrm {f}}(\xi )\sim N^{-3/4+\epsilon /6}$ according to (2.7), while ${{\,\mathrm{dist}\,}}(\xi ,{{\,\mathrm{supp}\,}}\rho _t)\sim N^{-3/4+3\epsilon /2}$.

For $z\in \widehat{\Gamma }\cup \widehat{\Upsilon }$ it follows from (5.26) that we can estimate5.28Indeed, for (5.28) we used (5.26) to obtain ${{\,\mathrm{dist}\,}}(\mathfrak {R}u,{{\,\mathrm{supp}\,}}\rho _t)\gtrsim t^{3/2}$, so that  follows from the local law from (2.8b).

We now distinguish three regimes: $\left| z\right| \lesssim N^{-\epsilon /2}$, $N^{-\epsilon /2}\lesssim \left| z\right| \ll 1$ and finally $\left| z\right| \gtrsim 1$ which we call microscopic, mesoscopic and macroscopic. We first consider the latter two regimes as they only contribute small error terms.

#### Macroscopic regime.

If either $\left| z\right| \ge \delta$ or $\left| w\right| \ge \delta$, it follows from Lemma 5.5 that $\mathfrak {R}g(w)\le -K$ and/or $\mathfrak {R}g(z)\ge K$, and therefore together with (5.23),(5.25) and (5.28) that $\mathfrak {R}\widetilde{f}(w)\lesssim -K$ and/or $\mathfrak {R}\widetilde{f}(z)\gtrsim K$ with overwhelming probability. Using $\Delta _0\sim N^{-3/4+3\epsilon /2}$ from (5.21a), we find that $N\Delta _0^2/ct\sim N^{2\epsilon }$ and $\Delta _0 N^{1/4}/ct\gamma \sim N^{\epsilon /2}$, so that the integrand in (5.19) in the considered regime is exponentially small.

#### Mesoscopic regime.

If either $\delta \ge \left| z\right| \gg N^{-\epsilon /2}$ or $\delta \ge \left| w\right| \gg N^{-\epsilon /2}$, then $\mathfrak {R}g(w)\sim -\left| w\right| ^4 \ll - N^{-2\epsilon }$ and/or $\mathfrak {R}g(z)\sim \left| z\right| ^4 \gg N^{-2\epsilon }$ from (5.27). Thus it follows from (5.23) and (5.25) that also $\mathfrak {R}f(w)\ll - N^{-2\epsilon }$ and/or $\mathfrak {R}f(z)\gg N^{-2\epsilon }$ and by (5.28) that with overwhelming probability $\mathfrak {R}\widetilde{f}(w)\ll - N^{-2\epsilon }$ and/or $\mathfrak {R}\widetilde{f}(z)\gg N^{-2\epsilon }$. Since $1/\left| w-z\right|$ is integrable over the contours it thus follows that the contribution to $\widetilde{K}_N^t(x,y)$, as in (5.19), from *z*, *w* with either $\left| z\right| \gg N^{-\epsilon /2}$ or $\left| w\right| \gg N^{-\epsilon /2}$ is negligible.

#### Microscopic regime.

We can now concentrate on the important regime where $\left| z\right| +\left| w\right| \lesssim N^{-\epsilon /2}$ and to do so perform another change of variables $z\mapsto ct \gamma z/\Delta _0 N^{1/4}\sim N^{-\epsilon /2} z$, $w\mapsto ct \gamma w/\Delta _0 N^{1/4}\sim N^{-\epsilon /2} w$ which gives rise to two new contours

$$\begin{aligned} \widehat{\Gamma }':=\frac{\Delta _0 N^{1/4}}{ct\gamma }\widehat{\Gamma },\qquad \widehat{\Upsilon }' :=\frac{\Delta _0 N^{1/4}}{ct\gamma }\widehat{\Upsilon }, \end{aligned}$$

as depicted in Fig. 2B, and the kernel

5.29 $$\begin{aligned} \widetilde{K}_N^t(x,y)= \frac{1}{(2\pi \mathrm {i})^2} \int _{{\widehat{\Upsilon }}'}\mathrm{d}z\int _{{\widehat{\Gamma }}'} \mathrm{d}w \frac{\exp \left( xz-yw + \frac{N\Delta _0^2}{ct}[\widetilde{f}(\frac{ct\gamma w}{\Delta _0 N^{1/4}})-\widetilde{f}(\frac{ct\gamma z}{\Delta _0 N^{1/4}})]\right) }{w-z}.\nonumber \\ \end{aligned}$$

We only have to consider *w*, *z* with $\left| w\right| +\left| z\right| \lesssim 1$ in (5.29) since $t/\Delta _0 N^{1/4}\sim N^{-\epsilon /2}$ and the other regime has already been covered in the previous paragraph before the change of variables.

We now separately estimate the errors stemming from replacing $\widetilde{f}(z)$ first by *f*(*z*), then by $\widetilde{g}(z)$ and finally by $\pm t^\rho z^2/2ct-4z^4/27$. We recall that $\Delta _0\sim t^{3/2}=N^{-3/4+3\epsilon /2}$ from (5.21a), $t^\rho \lesssim N^{-1/2}$ from the definition of $t^\rho$ in (5.21a), and that $t=N^{-1/2+\epsilon }$ which will be used repeatedly in the following estimates. According to (5.28), we have

5.30a $$\begin{aligned} \frac{N\Delta _0^2}{ct}\left| \widetilde{f}\Big (\frac{ct\gamma z}{\Delta _0 N^{1/4}}\Big )-f\Big (\frac{ct\gamma z}{\Delta _0 N^{1/4}}\Big )\right| \prec \frac{N\Delta _0^2}{t}\frac{t}{\Delta _0 N^{1/4}} N^{-2\epsilon } \left| z\right| \lesssim N^{-\epsilon /2}.\nonumber \\ \end{aligned}$$

Next, from (5.23) we have

5.30b $$\begin{aligned} \frac{N\Delta _0^2}{ct}\left| f\Big (\frac{ct\gamma z}{\Delta _0 N^{1/4}}\Big )-\widetilde{g}\Big (\frac{ct\gamma z}{\Delta _0 N^{1/4}}\Big )\right| \lesssim t^{1/3} \left| \frac{ct\gamma z}{\Delta _0 N^{1/4}}\right| ^2 \frac{N\Delta _0^2}{ct} + t^{1/3} \frac{N\Delta _0^2}{ct} \lesssim N^{-1/6+7\epsilon /3}.\nonumber \\ \end{aligned}$$

Finally, we have to estimate the error from replacing $\widetilde{g}(z)$ by its Taylor expansion with (5.24) and find

5.30c $$\begin{aligned} \frac{N\Delta _0^2}{ct} \left| \widetilde{g}\Big (\frac{ct\gamma z}{\Delta _0 N^{1/4}}\Big )-\frac{\pm t^\rho }{2ct}\Big (\frac{ct\gamma z}{\Delta _0 N^{1/4}}\Big )^2+\frac{4}{27} \Big (\frac{ct\gamma z}{\Delta _0 N^{1/4}}\Big )^4\right| \lesssim N^{-\epsilon /2}.\quad \end{aligned}$$

Finally, from (5.21a) and the definition of $\alpha$ from (2.6) we obtain that

5.30d $$\begin{aligned} \frac{N\Delta _0^2}{ct}\left[ \frac{\pm t^\rho }{2ct}\left( \frac{ct\gamma z}{\Delta _0 N^{1/4}}\right) ^2-\frac{4}{27}\left( \frac{ct\gamma z}{\Delta _0 N^{1/4}}\right) ^4\right] =\left( \alpha \frac{z^2}{2}-\frac{z^4}{4}\right) [1+{\mathcal {O}}\,\left( [\right) 0]{t^{1/3}}].\nonumber \\ \end{aligned}$$

From (5.30) and the integrability of $1/\left| z-w\right|$ for small *z*, *w* along the contours we can thus conclude

5.31 $$\begin{aligned} \widetilde{K}_N^t(x,y)= (1+{\mathcal {O}}\,\left( N^{-c}\right) )\frac{1}{(2\pi \mathrm {i})^2} \int _{\widehat{\Upsilon }'}\mathrm{d}z\int _{\widetilde{\Gamma }'} \mathrm{d}w \frac{e^{xz-yw + z^4/4 - \alpha z^2/2-w^4/4+\alpha w^2/2}}{w-z}.\nonumber \\ \end{aligned}$$

Furthermore, it follows from (5.26) that, as $N\rightarrow \infty$, the contours $\widehat{\Upsilon }',\widehat{\Gamma }'$ are those depicted in Fig. 2b, i.e.

$$\begin{aligned} \widehat{\Upsilon }'= (- e^{\mathrm {i}\pi /4}\infty ,e^{\mathrm {i}\pi /4}\infty ) \cup (- e^{-\mathrm {i}\pi /4}\infty ,e^{-\mathrm {i}\pi /4}\infty ),\qquad \widehat{\Gamma }':=(-\mathrm {i}\infty ,\mathrm {i}\infty ). \end{aligned}$$

We recognize (5.31) as the extended Pearcey kernel from (2.5).

It is easy to see that all error terms along the contour integration are uniform in *x*, *y* running over any fixed compact set. This proves that $\widetilde{K}_N^t(x,y)$ converges to $K_\alpha (x,y)$ uniformly in *x*, *y* in a compact set. This completes the proof of Proposition 5.3. $\square$

### Green function comparison

We will now complete the proof of Theorem 2.3 by demonstrating that the local *k*-point correlation function at the common physical cusp location $\tau _0$ of the matrices $\widetilde{H}_t$ does not change along the flow (5.1). Together with Proposition 5.3 this completes the proof of Theorem 2.3. A version of this *continuity of the matrix Ornstein-Uhlenbeck process* with respect to the local correlation functions that is valid in the bulk or at regular edges is the third step in the well known three step approach to universality [38]. We will present this argument in the more general setup of correlated random matrices, i.e. in the setting of [34]. In particular, we assume that the cumulants of the matrix elements $w_{ab}$ satisfy the decay conditions [34, Assumptions (C,D)], an assumption that is obviously fulfilled for deformed Wigner-type matrices.

We claim that the *k*-point correlation function $p_k^{(N)}$ of $H=\widetilde{H}_0$ and the corresponding *k*-point correlation function $\widetilde{p}_{k,t}^{(N)}$ of $\widetilde{H}_t$ stay close along the OU-flow in the sense that

5.32 $$\begin{aligned} \left| \int _{\mathbb {R}^k} F(\mathbf {x})\left[ N^{k/4} p_k^{(N)}\left( \mathfrak {b}+ \frac{\mathbf {x}}{\gamma N^{3/4}}\right) - \widetilde{p}_{k,t}^{(N)}\left( \mathfrak {b}+ \frac{\mathbf {x}}{\gamma N^{3/4}}\right) \right] \mathrm{d}x_1\ldots \mathrm{d}x_k\right| = {\mathcal {O}}\,\left( N^{-c}\right) ,\nonumber \\ \end{aligned}$$

for $\epsilon >0$, $t \le N^{-1/4-\epsilon }$, smooth functions *F* and some constant $c=c(k,\epsilon )$, where $\mathfrak {b}$ is the physical cusp point. The proof of (5.32) follows the standard arguments of computing *t*-derivatives of products of traces of resolvents $\widetilde{G}^{(t)}=(\widetilde{H}_t-z)$ at spectral parameters *z* just below the fluctuation scale of eigenvalues, i.e. for $\mathfrak {I}z\ge N^{-{\zeta }}\eta _f(\mathfrak {R}z)$. Since the procedure detailed e.g. in [38, Chapter 15] is well established and not specific to the cusp scaling, we keep our explanations brief.

The only cusp-specific part of the argument is estimating products of random variables

$$\begin{aligned} X_t=X_t(x):=N^{1/4} \left\langle \mathfrak {I}\widetilde{G}^{(t)}(\mathfrak {b}+\gamma ^{-1}N^{-3/4}x + \mathrm {i}N^{-3/4-\zeta })\right\rangle \end{aligned}$$

and we claim that

5.33 $$\begin{aligned} {{\,\mathrm{\mathbf {E}}\,}}\biggl [\prod _{j=1}^k X_t(x_j)-\prod _{j=1}^k X_0(x_j)\biggr ] \lesssim N^{-c} \end{aligned}$$

as long as $t \le N^{-1/4-\epsilon }$ for some $c=c(k,\epsilon ,\zeta )$. For simplicity we first consider $k=1$ and find from Itô’s Lemma that

5.34 $$\begin{aligned} {{\,\mathrm{\mathbf {E}}\,}}\frac{\mathrm{d}X_t}{\mathrm{d}t} = {{\,\mathrm{\mathbf {E}}\,}}\biggl [ -\frac{1}{2}\sum _\alpha w_\alpha \partial _\alpha X_t + \frac{1}{2}\sum _{\alpha ,\beta }\kappa (\alpha ,\beta )\partial _\alpha \partial _\beta X_t \biggr ], \end{aligned}$$

which we further compute using a standard cumulant expansion, as already done in the bulk regime in [34, Proof of Corollary 2.6] and in the edge regime in [11, Section 4.2]. We recall that $\kappa (\alpha ,\beta )$, and more generally $\kappa (\alpha ,\beta _1,\ldots ,\beta _k)$ denote the joint cumulants of the random variables $w_\alpha ,w_\beta$ and $w_\alpha ,w_{\beta _1},\ldots ,w_{\beta _k}$, respectively, which accordingly scale like $N^{-1}$ and $N^{-(k+1)/2}$. Here greek letters $\alpha ,\beta \in [N]^2$ are double indices. After cumulant expansion, the leading term in (5.34) cancels, and the next order contribution is

$$\begin{aligned} \sum _{\alpha ,\beta _1,\beta _2} \kappa (\alpha ,\beta _1,\beta _2) {{\,\mathrm{\mathbf {E}}\,}}\bigl [ \partial _\alpha \partial _{\beta _1}\partial _{\beta _2} X_t \bigr ], \end{aligned}$$

with $N^{-3/2}$ being the size of the cumulant $\kappa (\alpha ,\beta _1,\beta _2)$. With $\alpha =(a,b)$ and $\beta _i=(a_i,b_i)$ we then estimate

$$\begin{aligned} \begin{aligned}&N^{-3/4}\sum _{a,b,c}\sum _{a_1,b_1,a_2,b_2} \left| \kappa (ab,a_1b_1,a_2b_2)\right| {{\,\mathrm{\mathbf {E}}\,}}\left| \widetilde{G}_{ca}^{(t)}\widetilde{G}^{(t)}_{ba_1}\widetilde{G}^{(t)}_{b_1a_2}\widetilde{G}^{(t)}_{b_2c}\right| \\&\quad \le N^{-3/4-3/2+2+3/4+{\zeta }} \Vert \mathfrak {I}\widetilde{G}^{(t)}\Vert _3 \Vert \widetilde{G}^{(t)}\Vert _3^2, \end{aligned} \end{aligned}$$

where we used the Ward-identity and that $\max _\alpha \sum _{\beta _1,\beta _2}\kappa (\alpha ,\beta _1,\beta _2)\lesssim N^{-3/2}$. We now use that according to [34, Proof of Prop. 5.5], $\eta \mapsto \eta \Vert \widetilde{G}^{(t)}\Vert _p$ and similarly $\eta \mapsto \eta \Vert \mathfrak {I}\widetilde{G}^{(t)}\Vert _p$ are monotonically increasing with $\eta '=N^{-3/4+{\zeta }}$ to find $\Vert \mathfrak {I}\widetilde{G}^{(t)}\Vert _p\le _p N^{3{\zeta }-1/4}$ and $\Vert \widetilde{G}^{(t)}\Vert _p\le _p N^{3{\zeta }}$ from the local law from Theorem 2.5 and the scaling of $\rho$ at $\eta '$. Since all other error terms can be handled similarly and give an even smaller contribution it follows that

5.35 $$\begin{aligned} \left| {{\,\mathrm{\mathbf {E}}\,}}\frac{\mathrm{d}X_t}{\mathrm{d}t}\right| \lesssim N^{1/4+{C\zeta }}\quad \text {and similarly, but more generally,}\quad \left| {{\,\mathrm{\mathbf {E}}\,}}\frac{\mathrm{d}}{\mathrm{d}t} \prod _{j=1}^k X_t(x_j)\right| \lesssim N^{1/4+{Ck \zeta }},\nonumber \\ \end{aligned}$$

for some constant $C>0$. Now (5.33) and therefore (5.32) follow from (5.35) as in [38, Theorem 15.3] using the choice $t=N^{-1/2+\epsilon } \le N^{-1/4-\epsilon }$ and choosing $\zeta$ sufficiently small.

## Appendix A. Technical lemmata

### Lemma A.1

Let $\mathbb {C}^{N \times N}$ be equipped with a norm $\Vert \cdot \Vert$. Let ${\mathcal {A}}:\mathbb {C}^{N \times N} \times \mathbb {C}^{N \times N}\rightarrow \mathbb {C}^{N \times N}$ be a bilinear form and let ${\mathcal {B}}:\mathbb {C}^{N \times N} \rightarrow \mathbb {C}^{N \times N}$ a linear operator with a non-degenerate isolated eigenvalue $\beta$. Denote the spectral projection corresponding to $\beta$ by ${\mathcal {P}}$ and by ${\mathcal {Q}}$ the one corresponding to the spectral complement of $\beta$, i.e.

$$\begin{aligned} {\mathcal {P}}:=-\lim _{\epsilon \searrow 0}\frac{1}{2\pi \mathrm {i}}\oint _{\partial B_\epsilon (\beta )}\frac{\mathrm {d}\omega }{{\mathcal {B}}-\omega }= \left\langle V_\mathrm {l}, \cdot \right\rangle V_\mathrm {r},\qquad {\mathcal {Q}}:=1-{\mathcal {P}}, \end{aligned}$$

where $V_\mathrm {r}$ is the eigenmatrix corresponding to $\beta$ and $\left\langle V_\mathrm {l}, \cdot \right\rangle$ a linear functional. Assume that for some positive constant $\lambda >1$ the bounds

A.1 $$\begin{aligned} \Vert {\mathcal {A}}\Vert + \Vert {\mathcal {B}}^{-1}{\mathcal {Q}}\Vert + \Vert \left\langle V_\mathrm {l}, \cdot \right\rangle \Vert + \Vert V_\mathrm {r}\Vert \le \lambda , \end{aligned}$$

are satisfied, where we denote the induced norms on linear operators, linear functionals and bilinear forms on $\mathbb {C}^{N \times N}$ by the same symbol $\Vert \cdot \Vert$. Then there exists a universal constant $c>0$ such that for any $\delta \in (0,1)$ and any ${Y}, {X} \in \mathbb {C}^{N \times N}$ with $\Vert Y\Vert + \Vert {X}\Vert \le c\lambda ^{-4}$ that satisfies the quadratic equation

A.2 $$\begin{aligned} {\mathcal {B}}[{Y}] - {\mathcal {A}}[{Y},{Y}]+ {X}= 0, \end{aligned}$$

the following holds: The scalar quantity

$$\begin{aligned} \Theta :=\left\langle V_\mathrm {l},Y\right\rangle , \end{aligned}$$

fulfils the cubic equation

A.3 $$\begin{aligned} \mu _3 \Theta ^3+\mu _2 \Theta ^2 + \mu _1 \Theta + \mu _0= \lambda ^{12}{\mathcal {O}}\,\left( \delta \left| \Theta \right| ^3+\left| \Theta \right| ^4 + \delta ^{-2}\Vert {X}\Vert ^3\right) , \end{aligned}$$

with coefficients

A.4 $$\begin{aligned} \begin{aligned} \mu _3&=\left\langle V_\mathrm {l},{\mathcal {A}}[V_\mathrm {r},{\mathcal {B}}^{-1}{\mathcal {Q}}{\mathcal {A}}[V_\mathrm {r},V_\mathrm {r}]]+{\mathcal {A}}[{\mathcal {B}}^{-1}{\mathcal {Q}}{\mathcal {A}}[V_\mathrm {r},V_\mathrm {r}],V_\mathrm {r}]\right\rangle \\ \mu _2&=\left\langle V_\mathrm {l},{\mathcal {A}}[V_\mathrm {r},V_\mathrm {r}]\right\rangle \\ \mu _1&=-\left\langle V_\mathrm {l},{\mathcal {A}}[{\mathcal {B}}^{-1}{\mathcal {Q}}[{X}],V_\mathrm {r}]+{\mathcal {A}}[V_\mathrm {r},{\mathcal {B}}^{-1}{\mathcal {Q}}[{X}]]\right\rangle -\beta \\ \mu _0&=\left\langle V_\mathrm {l},{\mathcal {A}}[{\mathcal {B}}^{-1}{\mathcal {Q}}[{X}],{\mathcal {B}}^{-1}{\mathcal {Q}}[{X}]]-{X}\right\rangle . \end{aligned} \end{aligned}$$

Furthermore,

A.5 $$\begin{aligned} {Y}= \Theta V_\mathrm {r}- {\mathcal {B}}^{-1}{\mathcal {Q}}[{X}] +\Theta ^2{\mathcal {B}}^{-1}{\mathcal {Q}}{\mathcal {A}}[V_\mathrm {r},V_\mathrm {r}] +\lambda ^7{\mathcal {O}}\,\left( \left| \Theta \right| ^3+\left| \Theta \right| \Vert X\Vert +\Vert {X}\Vert ^2\right) .\qquad \end{aligned}$$

Here, the constants implicit in the $\mathcal {O}$-notation depend on *c* only.

### Proof

We decompose *Y* as

$$\begin{aligned} {Y}= {Y}_1+{Y}_2,\qquad {Y}_1 = \Theta V_\mathrm {r}- {\mathcal {B}}^{-1}{\mathcal {Q}}[{X}],\qquad {Y}_2= {\mathcal {Q}}[{Y}]+ {\mathcal {B}}^{-1}{\mathcal {Q}}[{X}]. \end{aligned}$$

Then (A.2) takes the form

A.6 $$\begin{aligned} \Theta \beta V_\mathrm {r} + {\mathcal {P}}[{X}]+{\mathcal {B}}{\mathcal {Q}}[{Y}_2]= {\mathcal {A}}[{Y},{Y}]. \end{aligned}$$

We project both sides with ${\mathcal {Q}}$, invert ${\mathcal {B}}$ and take the norm to conclude

$$\begin{aligned} \Vert {Y}_2\Vert = \lambda ^2\mathcal {O}(\Vert {Y}_1\Vert ^2 + \Vert {Y}_2\Vert ^2), \end{aligned}$$

Then we use the smallness of ${Y}_2$ by properly choosing $\delta$ and the definition of ${Y}_1$ to infer ${Y}_2= \lambda ^4 \mathcal {O}_2$, where we introduced the notation

$$\begin{aligned} \mathcal {O}_k = \mathcal {O}(\left| \Theta \right| ^k + \Vert {X}\Vert ^k). \end{aligned}$$

Inserting this information back into (A.6) and using $\left| \Theta \right| +\Vert X\Vert =\mathcal {O}(\lambda ^{-3})$ reveals

A.7 $$\begin{aligned} {Y}_2= {\mathcal {B}}^{-1}{\mathcal {Q}}{\mathcal {A}}[{Y}_1,{Y}_1] + \lambda ^7\mathcal {O}_3. \end{aligned}$$

In particular, (A.5) follows. Plugging (A.7) into (A.6) and applying the projection ${\mathcal {P}}$ yields

$$\begin{aligned} \begin{aligned} \Theta \beta V_\mathrm {r} + {\mathcal {P}}[{X}]&= {\mathcal {P}}\Big [ {\mathcal {A}}[{Y}_1,{Y}_1]+ {\mathcal {A}}[{Y}_1,{Y}_2]+ {\mathcal {A}}[{Y}_2,{Y}_1] \Big ] + \lambda ^{11}\mathcal {O}_4 \\&= {\mathcal {P}}\Big [ {\mathcal {A}}[{Y}_1,{Y}_1]+ {\mathcal {A}}[{Y}_1,{\mathcal {B}}^{-1}{\mathcal {Q}}{\mathcal {A}}[{Y}_1,{Y}_1]]+ {\mathcal {A}}[{\mathcal {B}}^{-1}{\mathcal {Q}}{\mathcal {A}}[{Y}_1,{Y}_1],{Y}_1] \Big ] + \lambda ^{11}\mathcal {O}_4. \end{aligned} \end{aligned}$$

For a linear operator ${\mathcal {K}}_1$ and a bilinear form ${\mathcal {K}}_2$ with $\Vert {\mathcal {K}}_1\Vert +\Vert {\mathcal {K}}_2\Vert \le 1$ we use the general bounds

$$\begin{aligned} \Theta {\mathcal {K}}_2[{R},{R}]\le \delta \Theta ^3 +\delta ^{-1/2} \Vert {R}\Vert ^{3},\qquad \Theta ^2{\mathcal {K}}_1[{R}] \le \delta \Theta ^3 + \delta ^{-2}\Vert {R}\Vert ^3, \end{aligned}$$

for any $R \in \mathbb {C}^{N \times N}$ and $\delta >0$ to find

$$\begin{aligned} \begin{aligned} \Theta \beta V_\mathrm {r} + {\mathcal {P}}[{X}]&= {\mathcal {P}}\Big [ {\mathcal {A}}[\Theta V_\mathrm {r}- {\mathcal {B}}^{-1}{\mathcal {Q}}[{X}],\Theta V_\mathrm {r}- {\mathcal {B}}^{-1}{\mathcal {Q}}[{X}]]+ \Theta ^3{\mathcal {A}}[V_\mathrm {r},{\mathcal {B}}^{-1}{\mathcal {Q}}{\mathcal {A}}[V_\mathrm {r},V_\mathrm {r}]]\\&\qquad \qquad + \Theta ^3{\mathcal {A}}[{\mathcal {B}}^{-1}{\mathcal {Q}}{\mathcal {A}}[V_\mathrm {r},V_\mathrm {r}],V_\mathrm {r}] \Big ] \\&\qquad +\lambda ^8 \mathcal {O}\big (\delta \left| \Theta \right| ^3+\lambda ^3 \left| \Theta \right| ^4+ \delta ^{-2}\Vert {X}\Vert ^3\big ), \end{aligned} \end{aligned}$$

which proves (A.3). $$\square $$

### Proof of Lemma 3.3

Due to the asymptotics $\Psi _{\mathrm {edge}}\sim \min \{\lambda ^{1/2},\lambda ^{1/3}\}$ and $\Psi _{\mathrm {min}}\sim \min \{\lambda ^2,\left| \lambda \right| ^{1/3}\}$ and the classification of singularities in (2.4), we can infer the following behaviour of the self-consistent fluctuation scale from Definition 2.4. There exists a constant $c>0$ only depending on the model parameters such that we have the following asymptotics. First of all, in the spectral bulk we trivially have that $\eta _\mathrm {f}(\tau )\sim N^{-1}$ as long as $\tau$ is at least a distance of $c>0$ away from local minima of $\rho$. In the remaining cases we use the explicit shape formulae from (2.4) to compute $\eta _\mathrm {f}$ directly from Definition 2.4.

- *Non-zero local minimum or cusp.* Let $\tau$ be the location of a non-zero local minimum $\rho (\tau )=\rho _0>0$ or a cusp $\rho (\tau )=\rho _0=0$. Then
  A.8a $$\begin{aligned} \eta _{\mathrm {f}}(\tau +\omega )\sim {\left\{ \begin{array}{ll}1/(N\max \{\rho _0, \left| \omega \right| ^{1/3}\}), &{} \max \{\rho _0, \left| \omega \right| ^{1/3}\} > N^{-1/4},\\ N^{-3/4}, &{} \max \{\rho _0, \left| \omega \right| ^{1/3}\} \le N^{-1/4}, \end{array}\right. } \end{aligned}$$
  for $$\omega \in (-c,c)$$.
- *Edge.* Let $\tau =\mathfrak {e}_\pm$ be the position of a left/right edge at a gap in ${{\,\mathrm{supp}\,}}\rho \cap (\mathfrak {e}_\pm -\kappa ,\mathfrak {e}_\pm + \kappa )$ of size $\Delta \in (0, \kappa ]$ (cf. (2.4b)). Then
  A.8b $$\begin{aligned} \eta _{\mathrm {f}}(\mathfrak {e}_\pm \pm \omega )\sim {\left\{ \begin{array}{ll} N^{-3/4}, &{} \omega \le \Delta \le N^{-3/4}, \\ \Delta ^{1/6}/\omega ^{1/2}N, &{}\Delta ^{1/9}/N^{2/3}< \omega \le \Delta , \\ \Delta ^{1/9}/N^{2/3}, &{} \omega \le \Delta ^{1/9}/N^{2/3},\; \Delta> N^{-3/4}, \\ N^{-3/4}, &{} \Delta < \omega \le N^{-3/4}, \\ 1/\omega ^{1/3}N, &{} \omega \ge N^{-3/4},\; \omega > \Delta , \end{array}\right. } \end{aligned}$$
  for $$\omega \in [0,c)$$.

The claimed bounds in Lemma 3.3 now follow directly from (3.7e) and (A.8) by distinguishing the respective regimes. $\square$

### Proof of Lemma 4.8

We start from (4.7) and estimate all vertex weights ${\varvec{w}}^{(v)}$, interaction matrices $R^{(e)}$ and weight matrices $K^{(e)}$ trivially by

to obtain

$$\begin{aligned} \left| {{\,\mathrm{Val}\,}}(\Gamma )\right| \le C^{\left| V\right| +\left| {{\,\mathrm{IE}\,}}\right| +\left| {{\,\mathrm{WE}\,}}\right| } N^{n(\Gamma )-\left| V\right| } \left\| \bigg (\prod _{v\in V} \sum _{a_v\in J}\bigg ) \prod _{e\in {{\,\mathrm{GE}\,}}} G_e \right\| _1. \end{aligned}$$

We now choose the vertex ordering $V=\{v_1,\ldots ,v_m\}$ as in Lemma 4.5. In the first step we partition the set of *G*-edges into three parts ${{\,\mathrm{GE}\,}}=E_1\cup E_2\cup E_3$: the edges not adjacent to $v_m$, $E_1={{\,\mathrm{GE}\,}}{\setminus } N(v_m)$, the non-Wardable edges adjacent to $v_m$, $E_2={{\,\mathrm{GE}\,}}\cap N(v_m){\setminus }{{\,\mathrm{{{\,\mathrm{GE}\,}}_{\text {W}}}\,}}$ and the Wardable edges adjacent to $v_m$, $E_3={{\,\mathrm{{{\,\mathrm{GE}\,}}_{\text {W}}}\,}}\cap N(v_m)$. By the choice of ordering it holds that $\left| E_3\right| \le 2$. We introduce the shorthand notation $G_{E_i}=\prod _{e\in E_i} G_e$ and use the general Hölder inequality for any collection of random variables $\{ X_A\}$ and $\{Y_A\}$ indexed by some arbitrary index set ${\mathcal {A}}$

$$\begin{aligned} \left\| \sum _{A\in {\mathcal {A}}} \left| X_A Y_A\right| \right\| _q \le \left\| \sum _{A\in {\mathcal {A}}}\left| X_A\right| \right\| _{q_1} \left| {\mathcal {A}}\right| ^{1/q_2} \max _{A\in {\mathcal {A}}} \Vert Y_A\Vert _{q_2}, \qquad \frac{1}{q}=\frac{1}{q_1}+\frac{1}{q_2} \end{aligned}$$

to compute

$$\begin{aligned} \begin{aligned}&\left\| \sum _{a_{v_1},\ldots ,a_{v_{m-1}}} \left| G_{E_1}\right| \sum _{a_{v_m}} \left| G_{E_2} G_{E_3}\right| \right\| _q \\&\qquad \le N^{(m-1)/q_2} \left\| \sum _{a_{v_1},\ldots ,a_{v_{m-1}}} \left| G_{E_1}\right| \right\| _{q_1}\max _{a_1,\ldots ,a_{v_{m-1}}} \Bigg ( \left\| \sum _{a_{v_m}}\left| G_{E_3}\right| \right\| _{2q_2} N^{1/2q_2} \max _{a_{v_m}} \Vert G_{E_2}\Vert _{2q_2} \Bigg ), \end{aligned} \end{aligned}$$

where we choose $1/q=1/q_1 + 1/q_2$ in such a way that $q_2\ge p/c\epsilon$. Since $\left| E_3\right| \le 2$ we can use (4.14a) to estimate

$$\begin{aligned} \left\| \sum _{a_{v_m}} \left| G_{E_3}\right| \right\| _{2q_2}\le N (\psi _{2q_2}')^{\left| E_3\right| }\le N (\psi +\psi _{2q_2}')^{\left| E_3\right| } \end{aligned}$$

and it thus follows from

$$\begin{aligned} \Vert G_{E_2}\Vert _{2q_2} \le \prod _{e\in E_2} \Vert G_e\Vert _{2\left| E_2\right| q_2} = \Vert G-M\Vert _{2\left| E_2\right| q_2}^{\left| E_2\cap {{\,\mathrm{GE}\,}}_{g-m}\right| } \Vert G\Vert _{2\left| E_2\right| q_2}^{\left| E_2{\setminus }{{\,\mathrm{GE}\,}}_{g-m}\right| } \end{aligned}$$

that

A.9 $$\begin{aligned}&\left\| \sum _{a_{v_1},\ldots ,a_{v_{m-1}}} \left| G_{E_1}\right| \sum _{a_{v_m}} \left| G_{E_2} G_{E_3}\right| \right\| _q \nonumber \\&\quad \le N^{\epsilon /c} \left\| \sum _{a_{v_1},\ldots ,a_{v_{m-1}}} \left| G_{E_1}\right| \right\| _{q_1} N(\psi +\psi _{q'}')^{\left| E_3\right| } (\psi +\psi _{q'}'+\psi _{q'}'')^{\left| E_2\cap {{\,\mathrm{GE}\,}}_{g-m}\right| }(1+\Vert G\Vert _{q'})^{\left| E_2\right| }\nonumber \\ \end{aligned}$$

for $q'\ge 2q_2\left| {{\,\mathrm{GE}\,}}\right|$. By using (A.9) inductively $m=\left| V\right| \le cp$ times it thus follows that

$$\begin{aligned} \left\| \bigg (\prod _{v\in V} \sum _{a_v\in J}\bigg ) \prod _{e\in {{\,\mathrm{GE}\,}}} G_e \right\| _1 \le N^{p\epsilon } N^{\left| V\right| } (\psi +\psi _{q'}')^{\left| {{\,\mathrm{{{\,\mathrm{GE}\,}}_{\text {W}}}\,}}\right| } (\psi +\psi _{q'}'+\psi _{q'}'')^{\left| {{\,\mathrm{GE}\,}}_{g-m}\right| } \big (1+\Vert G\Vert _{q'}\big )^{\left| {{\,\mathrm{GE}\,}}\right| }, \end{aligned}$$

proving the lemma. $\square$

### Lemma A.2

For the coefficient in (4.42) we have the expansion

A.10 $$\begin{aligned} \frac{\left\langle {\mathbf {b}}^{(B)}{\mathbf {p}}{\mathbf {f}}(R{\mathbf {b}}^{(B')})\right\rangle \left\langle {\mathbf {l}}^{(B')},\overline{{\mathbf {l}}^{(B)}}\right\rangle }{\left\langle \overline{{\mathbf {b}}^{(B)}},\overline{{\mathbf {l}}^{(B)}}\right\rangle \left\langle {\mathbf {l}}^{(B')},{\mathbf {b}}^{(B')}\right\rangle }= c \sigma \Vert F\Vert \left\langle \left| {\mathbf {m}}\right| ^{-2}{\mathbf {f}}^2\right\rangle + \mathcal {O}(\rho +{\eta /\rho }), \end{aligned}$$

for some $\left| c\right| \sim 1$, provided $\Vert B^{-1}\Vert _{\infty \rightarrow \infty } \ge C$ for some large enough constant $C>0$.

### Proof

Recall from the explanation after (4.42) that $R'=S,T,T^t$ if $R=S,T^t,T$, respectively. As we saw in the proof of Lemma 4.14, in the case $R=T,T^t$ in the complex Hermitian symmetry class, the operator *B* as well as $B'$ has a bounded inverse. Since we assume that $\Vert B^{-1}\Vert _{\infty \rightarrow \infty }$ is large, we have $R=R'=S$, which also includes the real symmetric symmetry class. In particular, we also have $\Vert (B')^{-1}\Vert _{\infty \rightarrow \infty }\ge C$ and all subsequent statements hold simultaneously for *B* and $B'$. We call ${\mathbf {f}}^{(S)}$ the normalised eigenvector corresponding to the eigenvalue with largest modulus of $F^{(S)}:=\left| M\right| S\left| M\right|$, recalling $M={{\,\mathrm{diag}\,}}({\mathbf {m}})$. Since $B=\left| M\right| (1-F^{(S)} + \mathcal {O}(\rho ))\left| M\right| ^{-1}$ we can use perturbation theory of $F^{(S)}$ to analyse spectral properties of *B*. In particular, we find

A.11 $$\begin{aligned} \begin{aligned} {\mathbf {b}}^{(B)}&= \left| M\right| {\mathbf {f}}^{(S)} + \mathcal {O}(\rho ),\qquad {\mathbf {l}}^{(B)}= \left| M\right| ^{-1}{\mathbf {f}}^{(S)}+ \mathcal {O}(\rho ), \\ B^{-1}Q_B&=\left| M\right| \big (1-F^{(S)} \big )^{-1}(1-P_{{\mathbf {f}}^{(S)}})\left| M\right| ^{-1}+ \mathcal {O}(\rho ), \end{aligned} \end{aligned}$$

where $P_{{\mathbf {f}}^{(S)}}$ is the orthogonal projection onto the ${\mathbf {f}}^{(S)}$ direction. The error terms are measured in $\Vert \cdot \Vert _\infty$-norm. For the expansions (A.11) we used that *F* has a spectral gap in the sense that

$$\begin{aligned} {{\,\mathrm{Spec}\,}}(F^{(S)} / \Vert F^{(S)}\Vert ) \subseteq [-1+c,1-c] \cup \{1\}, \end{aligned}$$

for some constant $c>0$, depending only on model parameters. By using (A.11) we see that the lhs. of (A.10) becomes $\pm \left\langle ({\mathbf {f}}^{(S)})^2 {\mathbf {p}}{\mathbf {f}}\right\rangle \Vert F^{(S)}\Vert \left\langle \left| {\mathbf {m}}\right| ^{-2}({\mathbf {f}}^{(S)})^2\right\rangle +{\mathcal {O}}\,\left( \rho \right)$. To complete the proof of the Lemma we note that ${\mathbf {f}}^{(S)}={\mathbf {f}}/\Vert {\mathbf {f}}\Vert +{\mathcal {O}}\,\left( \eta /\rho \right)$ according to [10, Eq. (5.10)].

$$\square $$

## Footnotes

1 See Appendix B of arXiv:1809.03971v2 for details.

2 This equivalent property is commonly known as having a *colouring number* of at most $k+1$, see e.g.  [39].

3 We have $c_1=\pi /\psi$, $c_2=2\sigma /\psi$ with the notations $\psi , \sigma$ in [10], where $\psi \sim 1$ and $\left| \sigma \right| \ll 1$ near the almost cusp, but we refrain from using these letters in the present context to avoid confusions.

4 See [10, Lemma 5.5] for the 1/3-Hölder continuity of quantities $\psi ,\sigma$ in the definition of $c_2$.

## References

[1] Adlam, B., Che, Z.: Spectral statistics of sparse random graphs with a general degree distribution. Preprint (2015). arXiv:1509.03368

[2] Adler M, Cafasso M, van Moerbeke P (2011). From the Pearcey to the Airy process. Electron. J. Probab..

[3] Adler M, Ferrari PL, van Moerbeke P (2010). Airy processes with wanderers and new universality classes. Ann. Probab..

[4] Adler M, van Moerbeke P (2007). PDEs for the Gaussian ensemble with external source and the Pearcey distribution. Commun. Pure Appl. Math..

[5] Ajanki, O.H., Erdős, L., Krüger, T.: Quadratic vector equations on complex upperhalf-plane. Mem. Amer. Math. Soc. **261**(1261), v+133 (2019)

[6] Ajanki OH, Erdős L, Krüger T (2017). Singularities of solutions to quadratic vector equations on the complex upper half-plane. Commun. Pure Appl. Math..

[7] Ajanki OH, Erdős L, Krüger T (2019). Stability of the matrix Dyson equation and random matrices with correlations. Probab. Theory Relat. Fields.

[8] Ajanki OH, Erdős L, Krüger T (2017). Universality for general Wigner-type matrices. Probab. Theory Relat. Fields.

[9] Alt, J., Erdős, L., Krüger, T.: Spectral radius of random matrices with independent entries. Preprint (2019). arXiv:1907.13631

[10] Alt, J., Erdős, L., Krüger, T.: The Dyson equation with linear self-energy: spectral bands, edges and cusps. Preprint (2018). arXiv:1804.07752

[11] Alt, J., Erdős, L., Krüger, T., Schröder, D.: Correlated random matrices: Band rigidity and edge universality. Ann. Probab. (2018). arXiv:1804.07744 (to appear)

[12] Anderson PW (1958). Absence of diffusion in certain random lattices. Phys. Rev..

[13] Baik, J., Kriecherbauer, T., McLaughlin, K.T.-R., Miller, P.D.: Discrete Orthogonal Polynomials, vol. 64. Annals of Mathematics Studies, Asymptotics and Applications, pp . viii+170. Princeton University Press, Princeton, NJ (2007)

[14] Bauerschmidt R, Huang J, Knowles A, Yau H-T (2017). Bulk eigenvalue statistics for random regular graphs. Ann. Probab..

[15] Bekerman F, Figalli A, Guionnet A (2015). Transport maps for $$\beta $$-matrix models and universality. Commun. Math. Phys..

[16] Borodin A, Okounkov A, Olshanski G (2000). Asymptotics of Plancherel measures for symmetric groups. J. Am. Math. Soc..

[17] Bourgade P, Erdős L, Yau H-T (2014). Edge universality of beta ensembles. Commun. Math. Phys..

[18] Bourgade P, Erdős L, Yau H-T (2014). Universality of general $$\beta $$-ensembles. Duke Math. J..

[19] Bourgade P, Erdős L, Yau H-T, Yin J (2017). Universality for a class of random band matrices. Adv. Theor. Math. Phys..

[20] Bourgade, P., Yau, H.-T., Yin, J.: Random band matrices in the delocalized phase, I: quantum unique ergodicity and universality. Preprint (2018). arXiv:1807.01559

[21] Brézin E, Hikami S (1998). Level spacing of random matrices in an external source. Phys. Rev. E.

[22] Brézin E, Hikami S (1998). Universal singularity at the closure of a gap in a random matrix theory. Phys. Rev. E.

[23] Capitaine M, Péché S (2016). Fluctuations at the edges of the spectrum of the full rank deformed GUE. Probab. Theory Relat. Fields.

[24] Cipolloni G, Erdős L, Krüger T, Schröder D (2019). Cusp universality for random matrices II: the real symmetric case. Pure Appl. Anal..

[25] Cipolloni, G., Erdős, L., Schröder, D.: Edge universality for non-Hermitian random matrices. Preprint (2019). arXiv:1908.00969 10.1007/s00440-020-01003-7 PMC7906960 33707804

[26] Claeys T, Kuijlaars ABJ, Liechty K, Wang D (2018). Propagation of singular behavior for Gaussian perturbations of random matrices. Commun. Math. Phys..

[27] Claeys T, Neuschel T, Venker M (2019). Boundaries of sine kernel universality for Gaussian perturbations of Hermitian matrices. Random Matrices Theory Appl..

[28] Deift P, Kriecherbauer T, McLaughlin KT-R (1998). New results on the equilibrium measure for logarithmic potentials in the presence of an external field. J. Approx. Theory.

[29] Deift P, Kriecherbauer T, McLaughlin KT-R, Venakides S, Zhou X (1999). Uniform asymptotics for polynomials orthogonal with respect to varying exponential weights and applications to universality questions in random matrix theory. Commun. Pure Appl. Math..

[30] Deift P, Gioev D (2007). Universality at the edge of the spectrum for unitary, orthogonal, and symplectic ensembles of random matrices. Commun. Pure Appl. Math..

[31] Duse E, Johansson K, Metcalfe A (2016). The cusp-Airy process. Electron. J. Probab..

[32] Erdős L, Knowles A, Yau H-T, Yin J (2012). Spectral statistics of Erdős–Renyi graphs II: eigenvalue spacing and the extreme eigenvalues. Commun. Math. Phys..

[33] Erdős L, Knowles A, Yau H-T, Yin J (2013). The local semicircle law for a general class of random matrices. Electron. J. Probab..

[34] Erdős L, Krüger T, Schröder D (2019). Random matrices with slow correlation decay. Forum Math. Sigma.

[35] Erdős L, Péché S, Ramírez JA, Schlein B, Yau H-T (2010). Bulk universality for Wigner matrices. Commun. Pure Appl. Math..

[36] Erdős L, Schlein B, Yau H-T (2011). Universality of random matrices and local relaxation flow. Invent. Math..

[37] Erdős L, Schnelli K (2017). Universality for random matrix flows with time-dependent density. Ann. Inst. Henri Poincaré Probab. Stat..

[38] Erdős, L., Yau, H.-T.: A Dynamical Approach to Random Matrix Theory, Vol. 28, Courant Lecture Notes in Mathematics, Courant Institute of Mathematical Sciences, pp. ix+226. American Mathematical Society, Providence, RI (2017)

[39] Erdős P, Hajnal A (1966). On chromatic number of graphs and set-systems. Acta Math. Acad. Sci. Hung..

[40] Geudens, D., Zhang, L.: Transitions between critical kernels: from the tacnode kernel and critical kernel in the two-matrix model to the Pearcey kernel. International Mathematics Research Notices IMRN 5733–5782 (2015)

[41] Guionnet A, Huang J (2019). Rigidity and edge universality of discrete $$\beta $$-ensembles. Comm. Pure Appl. Math.

[42] Hachem, W., Hardy, A., Najim, J.: A survey on the eigenvalues local behavior of large complex correlated Wishart matrices. In: Modelisation Aleatoire et Statistique—Journées MAS 2014, vol. 51, ESAIM Proceedings Surveys, EDP Sciences, Les Ulis, pp. 150–174 (2015)

[43] Hachem W, Hardy A, Najim J (2016). Large complex correlated Wishart matrices: fluctuations and asymptotic independence at the edges. Ann. Probab..

[44] Hachem W, Hardy A, Najim J (2016). Large complex correlated Wishart matrices: the Vearcey kernel and expansion at the hard edge. Electron. J. Probab..

[45] He Y, Knowles A (2017). Mesoscopic eigenvalue statistics of Wigner matrices. Ann. Appl. Probab..

[46] Helton, J. W., Rashidi Far, R., Speicher, R.: Operator-valued semicircular elements: solving a quadratic matrix equation with positivity constraints. International Mathematics Research Notices IMRN, Art. ID rnm086, 15 (2007)

[47] Huang J, Landon B, Yau H-T (2015). Bulk universality of sparse random matrices. J. Math. Phys..

[48] Johansson K (2001). Discrete orthogonal polynomial ensembles and the Plancherel measure. Ann. Math. (2).

[49] Johansson K (2001). Universality of the local spacing distribution in certain ensembles of Hermitian Wigner matrices. Commun. Math. Phys..

[50] Khorunzhy AM, Khoruzhenko BA, Pastur LA (1996). Asymptotic properties of large random matrices with independent entries. J. Math. Phys..

[51] Knowles A, Yin J (2017). Anisotropic local laws for random matrices. Probab. Theory Relat. Fields.

[52] Krishnapur M, Rider B, Virág B (2016). Universality of the stochastic Airy operator. Commun. Pure Appl. Math..

[53] Landon B, Yau H-T (2017). Convergence of local statistics of Dyson Brownian motion. Commun. Math. Phys..

[54] Landon, B., Yau, H.-T.: Edge statistics of Dyson Brownian motion. Preprint (2017). arXiv:1712.03881

[55] Lee JO, Schnelli K (2015). Edge universality for deformed Wigner matrices. Rev. Math. Phys..

[56] Lee JO, Schnelli K (2018). Local law and Tracy-Widom limit for sparse random matrices. Probab. Theory Relat. Fields.

[57] Lee JO, Schnelli K, Stetler B, Yau H-T (2016). Bulk universality for deformed Wigner matrices. Ann. Probab..

[58] Lick DR, White AT (1970). k-degenerate graphs. Can. J. Math..

[59] Mehta ML (1967). Random Matrices and the Statistical Theory of Energy Levels.

[60] Okounkov A, Reshetikhin N (2007). Random skew plane partitions and the Vearcey process. Commun. Math. Phys..

[61] Pastur L, Shcherbina M (2008). Bulk universality and related properties of Hermitian matrix models. J. Stat. Phys..

[62] Pastur L, Shcherbina M (2003). On the edge universality of the local eigenvalue statistics of matrix models. Mat. Fiz. Anal. Geom..

[63] Pearcey T (1946). The structure of an electromagnetic field in the neighbourhood of a cusp of a caustic. Philos. Mag..

[64] Shcherbina M (2014). Change of variables as a method to study general $$\beta $$-models: bulk universality. J. Math. Phys..

[65] Shcherbina M (2009). Edge universality for orthogonal ensembles of random matrices. J. Stat. Phys..

[66] Sodin S (2010). The spectral edge of some random band matrices. Ann. Math. (2).

[67] Soshnikov A (1999). Universality at the edge of the spectrum in Wigner random matrices. Commun. Math. Phys..

[68] Tao T, Vu V (2011). Random matrices: universality of local eigenvalue statistics. Acta Math..

[69] Tao T, Vu V (2010). Random matrices: universality of local eigenvalue statistics up to the edge. Commun. Math. Phys..

[70] Tracy CA, Widom H (1994). Level-spacing distributions and the Airy kernel. Commun. Math. Phys..

[71] Tracy CA, Widom H (1996). On orthogonal and symplectic matrix ensembles. Commun. Math. Phys..

[72] Tracy CA, Widom H (2006). The Pearcey process. Commun. Math. Phys..

[73] Valkó B, Virág B (2009). Continuum limits of random matrices and the Brownian carousel. Invent. Math..

